# In Silico Study of the Potential Inhibitory Effects on *Escherichia coli* DNA Gyrase of Some Hypothetical Fluoroquinolone–Tetracycline Hybrids

**DOI:** 10.3390/ph17111540

**Published:** 2024-11-16

**Authors:** Ioana-Andreea Lungu, Octavia-Laura Oancea, Aura Rusu

**Affiliations:** 1 Medicine and Pharmacy Doctoral School, George Emil Palade University of Medicine, Pharmacy, Science, and Technology of Targu Mures, 540142 Targu Mures, Romania; 2 Organic Chemistry Department, Faculty of Pharmacy, George Emil Palade University of Medicine, Pharmacy, Science, and Technology of Targu Mures, 540142 Targu Mures, Romania; octavia.moldovan@umfst.ro; 3 Pharmaceutical and Therapeutic Chemistry Department, Faculty of Pharmacy, George Emil Palade University of Medicine, Pharmacy, Science, and Technology of Targu Mures, 540142 Targu Mures, Romania; aura.rusu@umfst.ro

**Keywords:** fluoroquinolone, tetracycline, hybrid, albicidin, molecular docking, DNA gyrase

## Abstract

Background/Objectives: Despite the discovery of antibiotics, bacterial infections persist globally, exacerbated by rising antimicrobial resistance that results in millions of cases, increased healthcare costs, and more extended hospital stays. The urgent need for new antibacterial drugs continues as resistance evolves. Fluoroquinolones and tetracyclines are versatile antibiotics that are effective against various bacterial infections. A hybrid antibiotic combines two or more molecules to enhance antimicrobial effectiveness and combat resistance better than monotherapy. Fluoroquinolones are ideal candidates for hybridization due to their potent bactericidal effects, ease of synthesis, and ability to form combinations with other molecules. Methods: This study explored the mechanisms of action for 40 hypothetical fluoroquinolone–tetracycline hybrids, all of which could be obtained using a simple, eco-friendly synthesis method. Their interaction with *Escherichia coli* DNA Gyrase and similarity to albicidin were evaluated using the FORECASTER platform. Results: Hybrids such as Do-Ba, Mi-Fi, and Te-Ba closely resembled albicidin in physicochemical properties and FITTED Scores, while Te-De surpassed it with a better score. Similar to fluoroquinolones, these hybrids likely inhibit DNA synthesis by binding to enzyme–DNA complexes. Conclusions: These hybrids could offer broad-spectrum activity and help mitigate bacterial resistance, though further in vitro and in vivo studies are needed to validate their potential.

## 1. Introduction

Bacterial infections still affect hundreds of millions of patients worldwide despite the revolutionary discovery of antibiotics [1]. These conditions, especially infections associated with surgical interventions, represent a global problem, especially but not exclusively in low- and middle-income countries [2]. A recent Centers for Disease Control and Prevention (CDC) report underlines a worrisome statistic. The number of people with antimicrobial-resistant infections in the United States went from more than 2 million (in 2013) to more than 2.8 million (in 2019) [3]. The impact is both social and financial, imposing substantial healthcare costs (billions of dollars) and causing patients to spend more than 400,000 additional days in the hospital [2,4]. Moreover, antimicrobial resistance constantly keeps pace with new drugs introduced into therapy, being, unfortunately, a constantly evolving phenomenon [5,6,7,8,9].

The need to discover new effective antimicrobial drugs to combat infectious diseases is evident from the arguments illustrated above.

Regarding spectrum, route of administration, and tissue distribution, fluoroquinolones (FQNs) are a valuable class of antimicrobial agents. The mechanism of action causes a bactericidal effect following interaction with the target enzymes (DNA gyrase (also known as topoisomerase II) and topoisomerase IV), inhibiting DNA replication and transcription [10,11,12,13]. Moreover, a significant advantage of this class is represented by their molecular versatility, which allows improvement of their pharmacokinetic and pharmacodynamic properties [14,15,16,17].

Currently, these molecules (classical and modern representatives of the new generations) are prescribed to treat infections in various locations, such as urinary tract infections, respiratory tract infections, and several types of gastrointestinal tract infection caused by Gram-negative and Gram-positive bacteria [18,19]. The widespread use of FQNs in humans and animals raises, as with other antibiotics, reasons for concern about the emergence of resistance [7,20]. Consequently, there is a pressing need to continuously discover new derivatives to keep pace with bacterial adaptation.

In addition to their use as antibacterial agents, certain FQN-derived compounds have various other therapeutic uses, with applicability in multiple pathologies, such as tuberculosis, malaria, viral infections, fungal infections, cancer, immunosuppression, and neurodegenerative diseases. Moreover, the chemical properties of FQNs, especially their chemical reactivity and structure, are still keeping researchers’ interest high [21].

The history of tetracyclines (TCs) goes back to the late 1940s, when they were first isolated from *Streptomyces* spp. [22,23]. TCs are broad-spectrum antibacterial agents, active against Gram-positive and Gram-negative bacteria and pathogens such as chlamydiae, mycoplasmas, rickettsiae, and protozoan parasites [23]. Their mechanism of action involves the inhibition of protein synthesis through interaction with the ribosomes (more specifically, the 16S RNA in the small (30S) ribosomal subunit) [24]. However, TCs exhibit some biological activities (apart from the antimicrobial effect) that cannot be explained by this mechanism, such as antiapoptotic [25,26], anti-inflammatory [26,27,28], antiviral [25,29,30], and neuroprotective [26,31] activities. The mechanisms involved in these effects are still under study [24].

TCs have been extensively used to treat infections in humans and animals, primarily for indications such as infections of the respiratory tract, urinary tract, genitals, lymph nodes, skin, intestines, and other body systems. Moreover, they have been administered to prevent malaria caused by *Plasmodium falciparum* resistant to mefloquine [22,32]. Due to their extensive use, resistance emerged soon after the early antibiotic era in *Streptococcus* spp. Currently, the most common mechanisms for resistance are ribosomal protection proteins and efflux pumps in both Gram-positive (e.g., *Staphylococcus* spp., *Streptococcus* spp.) and Gram-negative (e.g., *Klebsiella* spp., *Escherichia* spp.) pathogens [33]. Still, TCs remain an essential class of antimicrobials, offering a valuable therapeutic option for numerous bacterial infections [33].

Combination therapy has been a key approach among the various strategies employed to treat resistant bacteria and counteract their resistance mechanisms. This aspect involves using antimicrobial agents with multiple mechanisms of action within the same treatment [34]. For example, the concept of combination therapy proved successful in slowing the emergence of resistance in the treatment of *Mycobacterium tuberculosis* infections. However, some reports indicate that synergistic drug combinations may accelerate the development of resistance instead of inhibiting it. Another disadvantage of combination therapy is that the in vitro effects of a particular drug combination do not always translate to clear in vivo outcomes due to differing pharmacokinetic properties of the combined drugs. Additionally, this approach fails to solve the issue of multidrug-resistant strains that have developed resistance to both drug classes used together, necessitating the inclusion of other drug families. The limited number of potent antibacterial drug families further restricts this strategy’s effectiveness.

The hybrid antibiotic approach, on the other hand, by covalently linking two pharmacophores that act on different targets within a bacterial cell into a single molecule, may overcome the existing resistance to either or both drugs. The studies focused on hybrid antibiotics address their effectiveness against drug-resistant bacteria, their expanded range of activity, their prolonged effectiveness after the emergence of resistance, and the decreased likelihood of promoting further bacterial resistance [34,35,36]. Based on the existing literature, we hypothesized that the hybrids presented in this study may contribute to reducing bacterial resistance mainly by addressing more than one target (a dual mechanism of action granted by the two components).

Regarding the inclusion in hybrid compounds, FQNs are promising candidates for this strategy to combat antimicrobial resistance (and implicitly infections) due to a series of advantages: the mechanism of action that confers a bactericidal effect; their efficacy and potency; and the slower development of antimicrobial resistance, especially for newer agents, due to their dual activity against both target enzymes [37,38,39].

In addition to the advantages related to the antibacterial activity, FQNs also present a benefit from a chemical point of view. Their structures are relatively easy to synthesize, thus allowing the development of numerous potential derivatives with various advantageous features [14,37,40,41]. Their chemical structure shows excellent complexation properties with metal ions and the ability to form combinations with other active molecules [42,43]. The structural properties of FQNs have led to their use as a base for numerous attempts to hybridize and develop new antibacterial agents over time while underlining the promising role of hybridization in addressing antimicrobial resistance on a global scale [44,45]. To date, numerous studies have been carried out in which FQNs have been included in various hybrids with other molecules (either other antibiotics, such as tetracyclines, aminoglycosides, oxazolidinones, etc., or non-antibiotics, such as benzofuroxanes, benzimidazole, triazoles, etc.) [46,47,48,49,50,51,52,53].

We chose to study in more detail possible hybrids between FQNs and TCs that could be obtained through the method described by Sriram et al. [46] since the synthesis method seems relatively simple and is suitable for green chemistry, using microwave radiation and relatively non-toxic solvents (ethanol).

According to our knowledge, no cryo-electron microscopy or X-ray crystallography structures of a complex with these hybrids and DNA gyrase have been published. Thus, we have computationally designed 40 TC-FQN hybrid molecules. We proposed exploring the mechanisms of action of our hypothetical hybrids related to the FQN component based on literature data and how FQNs bind to DNA gyrase. We studied in silico the binding modes of the hypothetical hybrids to the DNA gyrase enzyme.

Apart from FQNs, there are other molecules that bind to DNA gyrase; recently, albicidin, a substance produced by *Xanthomonas albilineans* (a sugarcane pathogen), has been discovered. This compound has been shown to inhibit DNA gyrase similarly to quinolones, mainly by impeding the rejoining of cleaved DNA intermediates in the gyrase catalytic sequence [54].

The primary aim of this paper is to study the mechanism of action of the hypothetical TC-FQN hybrids using molecular docking, more specifically, their ability to bind to and inhibit DNA gyrase. This objective derives from our hypothesis that the TC-FQN hybrids will bind to DNA gyrase similarly to FQNs. Another objective of this study is to compare the hybrids with the known antibiotic albicidin in terms of molecular similarity and physicochemical properties.

## 2. Results

### 2.1. Designing the Hybrids

The makeup of the structures of the 40 hypothetical TC-FQN hybrids is presented in Table 1. The general structure of the hybrids is TC component–linker (–CH_2_– from formaldehyde)–FQN component. Each hybrid is identified by the TC component and the FQN component and was assigned a code derived from the first two letters of the TC component and the first two letters of the FQN component. Therefore, the codes are Do-Ba, Do-Be, Do-Ci, Do-De, Do-Fi, Do-Mo, Do-Ne, Do-No, Do-Si, Do-Za, Mi-Ba, Mi-Be, Mi-Ci, Mi-De, Mi-Fi, Mi-Mo, Mi-Ne, Mi-No, Mi-Si, Mi-Za, Te-Ba, Te-Be, Te-Ci, Te-De, Te-Fi, Te-Mo, Te-Ne, Te-No, Te-Si, Te-Za, Ti-Ba, Ti-Be, Ti-Ci, Ti-De, Ti-Fi, Ti-Mo, Ti-Ne, Ti-No, Ti-Si, and Ti-Za.

### 2.2. Determining the Similarity Between the TC-FQN Hybrids and the Co-Crystallized Ligand

The molecular similarity calculated between the hybrids and albicidin (the co-crystallized ligand) using the MACCS fingerprint scheme and the Tanimoto coefficient as the similarity measure with the “Chemical library cleaning” SELECT (Selection and Extraction of Libraries Employing Clustering Techniques) function of FORECASTER is presented in Table 2.

### 2.3. Comparing the Hybrids with the Co-Crystallized Ligand Regarding Physicochemical Properties

Selected physicochemical parameters of the designed structures and the co-crystallized ligand (albicidin), calculated using the SMART (Small Molecule Atom typing and Rotatable Torsions assignment) function of FORECASTER, are presented in Table 3. All the parameters calculated by the software can be found in Table S1.

### 2.4. Molecular Docking

#### 2.4.1. Self-Docking of Albicidin

**Figure 1:**
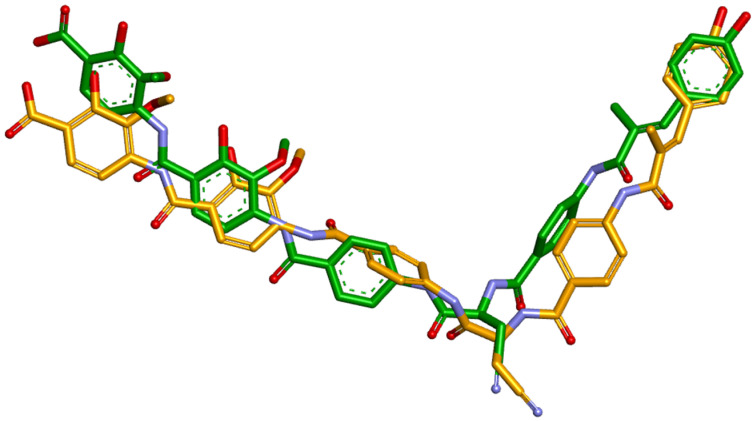
Superposition of the ligand (albicidin) in the original (crystallized structure) conformation (the structure with the green carbon atoms) and the ligand (albicidin) in the self-docked conformation (the structure with the orange carbon atoms).

The self-docking step was performed to evaluate the software’s ability to assess the interaction between the hybrids and the target enzyme. The docking of albicidin to *E. coli* gyrase holocomplex with 217 bp DNA was accomplished using the “Docking small molecule(s) to protein(s)” function of FORECASTER and the file “7z9c.pdb”, downloaded from PDB. The results were as follows: energy of −171.899 kcal/mol; root mean square deviation (RMSD) of 2.36 Å; Rank Score of −26.846; Match Score of 39.746; FITTED Score of −33.2054 (the values represent the best out of five runs ranked by docking score). The binding site amino acid residues were as follows: LYS64, LYS65, SER66, ALA67, ARG68, VAL69, VAL70, GLY71, ASP72, VAL73, ILE74, GLY75, LYS76, PRO79, HISD80, GLY81, ASP82, SER83, ALA84, MET120, ARG121, TYR149, GLY425, ASP426, SER427, LEU446, LYS447, GLY448, LYS449, GLU466, LYS740, GLY741, LEU742, GLY743, GLU744, MET745, LYS65, SER66, ALA67, ARG68, VAL69, VAL70, GLY71, ASP72, VAL73, ILE74, GLY75, LYS76, ASP82, TYR86, MET120, ARG121, PTR122 (O-phosphotyrosine), and LYS740. Figure 1 shows the position of the ligand in the original conformation (from the crystallized structure) and the self-docked conformation.

**Figure 2:**
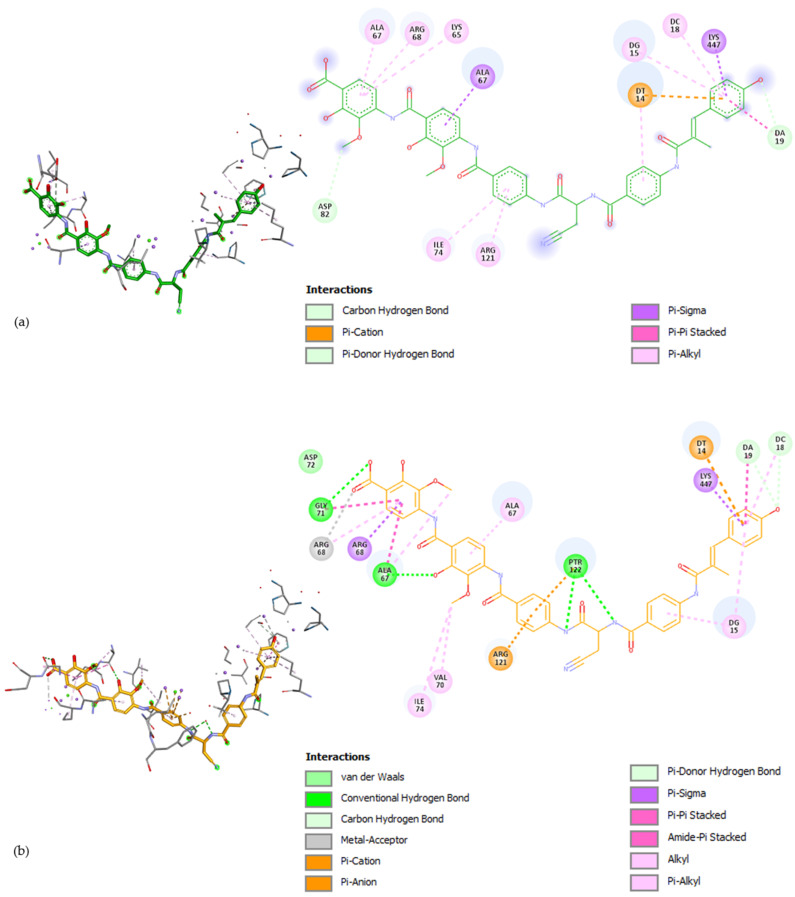
Interactions of albicidin with the binding pocket of *E. coli* gyrase holocomplex with 217 bp DNA obtained in the self-docking phase for albicidin (in 3D (left) and 2D (right)): (**a**) ligand in the original (crystallized structure) conformation (the structure with the green carbon atoms), (**b**) ligand in the self-docked conformation (the structure with the orange carbon atoms).

Figure 2 presents the interactions with the binding pocket of the ligand (albicidin) in the original conformation (from the crystallized structure) and the self-docked conformation.

#### 2.4.2. Docking of the Hybrids

**Figure 3:**
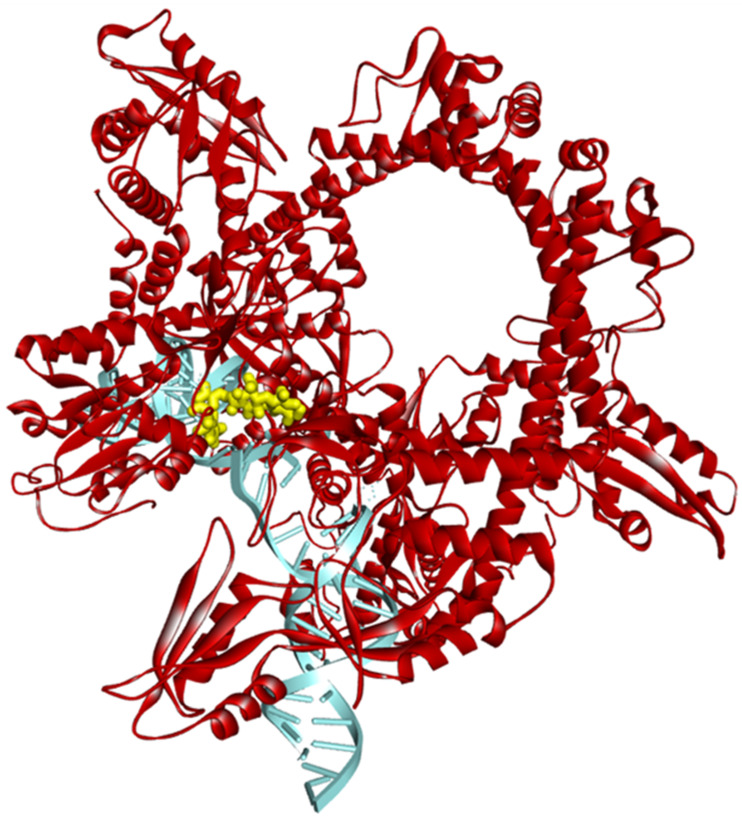
The conformation of the Te-De hybrid (yellow) in the pocket of the *E. coli* DNA gyrase enzyme (red)–DNA (blue) complex.

**Figure 4:**
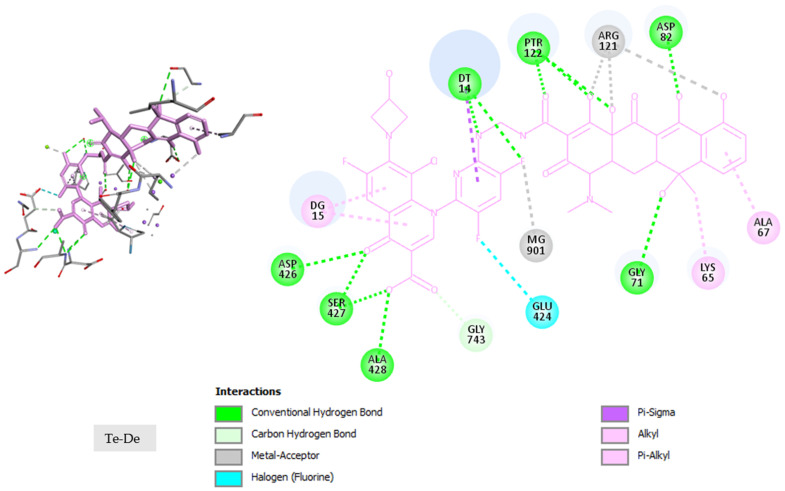
Interactions of the Te-De hybrid with the binding pocket of *E. coli* gyrase holocomplex with 217 bp DNA obtained in the self-docking phase for albicidin (in 3D (left) and 2D (right)).

All 40 hybrids were docked in the binding site of *E. coli* gyrase holocomplex with 217 bp DNA obtained in the self-docking phase for albicidin. The docking results (after five runs) are listed in Table 4. The docking results for albicidin are also included in the last row so that the values can be compared more easily with those obtained for the hybrid molecules. Figure 3 illustrates the conformation of the hybrid with the highest FITTED Score (Te-De) in the binding pocket of the enzyme–DNA complex, and Figure 4 shows the interactions of the Te-De hybrid with the binding site. Similar results for the conformations of all the hybrids in the binding site and their interactions with the binding pocket of the enzyme–DNA complex are presented in Figures S1 and S2.

## 3. Discussion

### 3.1. Designing the Hybrids and Selecting the Target

The design of the hybrids had as its starting point a study by Sriram D. et al. (2007), who synthesized beta-amino-ketones (Mannich bases) of tetracyclines. Their method used microwave irradiation (3 min, 60% intensity) to promote the reaction between tetracycline representatives (tetracycline, oxytetracycline, and minocycline), formaldehyde (as a molecular connector), and fluoroquinolone representatives (norfloxacin, lomefloxacin, ciprofloxacin, and gatifloxacin) that would supply the secondary amino function (piperazine) [46].

We chose to design TC-FQN hybrids because of the simple one-step synthesis method, in line with the third (less hazardous synthesis) and sixth (design for energy efficiency) principles of green chemistry by using microwave irradiation and ethanol, a less toxic and more environmentally friendly solvent (compared to formaldehyde or dioxane, for example) [55,56].

Our search in PDB excluded enzymes and enzyme fragments without ligands, as ligands were necessary for molecular docking validation (self-docking). Similarly, enzymes and enzyme fragments without DNA were excluded since the mechanism of action for FQNs targets the enzyme–DNA complex. Moreover, the affinity appears stronger for these complexes than for the enzymes alone [12,20,57,58].

From the co-crystallized structures available in PDB, we selected a target whose ligand was bound at the same site as FQNs: *E. coli* DNA gyrase with albicidin as a ligand [59]. Considering the FQN component of the hybrids and our hypothesis that they may bind to the enzyme in much the same way FQNs do, albicidin was a good fit for evaluating our premise.

### 3.2. Determining the Similarity Between the TC-FQN Hybrids and the Co-Crystallized Ligand

Virtual screening is a computational method widely employed as a cost-effective alternative to traditional high-throughput screening (HTS) for identifying initial hits in searching for drugs with specific biological activities [60,61]. Similarity-based virtual screening is founded on the structure–activity relationship (SAR), which suggests that molecules with similar structures tend to have similar biological activities. Therefore, quantifying the structural similarity of molecules is a crucial step in such virtual screening applications [62,63].

The Tanimoto coefficient is an association coefficient recognized as one of the most commonly employed similarity measures in molecular structure studies for similarity searching [64]. Association coefficients are frequently utilized with binary data, where variables indicate the presence or absence of descriptors for an object. They are commonly normalized to range from zero (indicating no similarity) to one (representing identical sets of descriptors) [65]. The Tanimoto coefficient applies to fingerprint features based on the molecules’ two-dimensional (2D) structures. These 2D fingerprints represent each molecule as a binary vector, indicating the absence or presence of specific properties in its 2D structure. Despite its simplicity, this feature representation has been reported to be superior regarding efficiency compared to those using more complex features, such as 3D structural patterns [66].

Libraries of predefined 2D chemical fingerprint dictionaries are available to represent molecules as binary vectors [67]. One of the most commonly used fingerprint schemes for similarity quantification is the Molecular ACCess System (MACCS) [68,69], which has been reported to encompass many useful 2D features for virtual screening [70].

The similarity threshold for the Tanimoto coefficient is not a fixed value and depends on the specific application and context; however, in some fields, there might be accepted thresholds. In cheminformatics, a Tanimoto coefficient of 0.85 or higher might indicate that two chemical compounds are similar enough to have similar biological properties [71,72].

For the tested hybrids, the Tanimoto coefficient was situated in the range of 0.59–0.68 (with an average value of 0.65), which is below the 0.85 threshold. However, for toxicological read-across, a cut-off of 0.7 was used by some authors [70,73]. Moreover, a similar physicochemical profile between the hybrids and albicidin (such as the numbers of hydrogen bond donors and acceptors, molecular weight, logP, logS, rotatable bonds, topological polar surface area (tPSA), and span) might compensate for the difference.

Very close values (differences within 0.01, except for delafloxacin, where the difference was within 0.02) were obtained for the hybrids with the same FQN, as can be seen in Table 2 (Tanimoto coefficients of 0.67 and 0.68 for balofloxacin hybrids, 0.65 and 0.66 for besifloxacin hybrids, 0.66 and 0.67 for ciprofloxacin hybrids, 0.64 to 0.66 for delafloxacin hybrids, 0.63 and 0.64 for finafloxacin hybrids, 0.64 and 0.65 for moxifloxacin hybrids, 0.67 and 0.68 for nemonoxacin hybrids, 0.66 and 0.67 for norfloxacin hybrids, 0.64 and 0.65 for sitafloxacin hybrids, and 0.59 and 0.60 for zabofloxacin hybrids). Thus, the FQN part apparently influenced the differences across the results. This aspect is not surprising, given that more structural differences exist among the FQNs selected for the hybrids than among the selected TCs (tigecycline being the most different).

Very similar results (differences within 0.01) were also obtained for balofloxacin and nemonoxacin hybrids (Tanimoto coefficients of 0.67 and 0.68), as expected, given their very similar structures. The same amount of close similarity (Tanimoto coefficients of 0.66 and 0.67) can be observed for the hybrids with ciprofloxacin and norfloxacin, whose structures are also much alike.

The highest values (between 0.66–0.68) were obtained by balofloxacin, nemonoxacin, norfloxacin, and ciprofloxacin hybrids (best Tanimoto coefficient of 0.68 for Te-Ba, Ti-Ba, and Ti-Ne). In contrast, the lowest values were obtained for the zabofloxacin hybrids (a minimum Tanimoto coefficient value of 0.59 for Mi-Za and values of 0.60 for Do-Za, Te-Za, and Ti-Za).

The four FQNs contained by the hybrids with the highest scores are the only ones with a six-atom heterocycle at position 7, the others having heterocycles with fewer or more atoms (as illustrated in Table 1); this aspect is consistent with the fact that albicidin has only six-atom aromatic rings in its structure.

Other similarities between the structure of albicidin and the structures of the hybrids are the presence of the carboxamide moiety (from position 2 of TCs and additionally from position 9 in the case of Tigecycline), carbonylic oxygens, a carboxyl moiety at one end of the molecule (from position 3 of FQNs), and a phenolic hydroxyl group at the other end (from position 10 of doxycycline, minocycline, and tetracycline).

### 3.3. Comparing the Hybrids with the Co-Crystallized Ligand Regarding the Physicochemical Properties

We characterized the hybrids and albicidin regarding structural and physicochemical properties, intending to see how similar the structures are concerning these features. The obtained values of the parameters discussed below are presented in Table 3.

Lipinski’s “Rule of 5” suggests that poor absorption is more likely if a compound has more than five hydrogen bond donors, more than 10 hydrogen bond acceptors, a molecular weight exceeding 500, and a calculated logP greater than five [74].

The tested molecules had several hydrogen bond donors (between eight and twelve) and hydrogen bond acceptors (between ten and fifteen). Albicidin had nine hydrogen bond donors and twelve hydrogen bond acceptors, as did Do-Ba, Te-Ba, Do-Fi, Te-Fi, Do-Mo, Te-Mo, and Mi-De.

In terms of molecular weight, the closest hybrids to albicidin (842.818 g/mol) were Mi-Ne, Do-Ba, and Te-Ba, at 842.947 g/mol, 847.894 g/mol, and 847.894, respectively. Although all the hybrids, as well as albicidin, exceed Lipinski’s suggested limit of 500, there are several molecules currently used in therapy that also break this rule (such as erythromycin, azithromycin, and cefiderocol, just to give a few examples).

The logarithmic partition coefficient (logP), a measure of lipophilicity, reflects how a compound distributes between lipid and aqueous phases. Hydrophilic compounds have a negative logP, indicating a greater preference for the aqueous phase. In contrast, lipophilic compounds exhibit a positive logP, showing a stronger affinity for the lipid or organic phase. According to Lipinski’s guidelines for assessing the drug-likeness of new synthetic compounds, an oral drug should ideally have a logP of <5, with the optimal range for absorption being 1.35 to 1.8 [75,76,77]. The highest logP value was obtained for the Ti-Be hybrid (8.9158), followed by albicidin (8.70223). The lowest values were obtained for the zabofloxacin hybrids (4.18875 for Te-Za, 5.08481 for Mi-Za, 5.31606 for Do-Za, and 5.37239 for Ti-Za). The only hybrid that fulfilled Lipinski’s rule regarding logP was Te-Za. However, several hybrids had results similar to that of albicidin (8.67628 for Ti-Si, 8.64016 for Do-Be, 8.43635 for Mi-Be, 8.42228 for Do-Si, and 8.41774 for Ti-Mo).

A compound’s solubility is typically expressed as logS, where S represents the compound’s concentration (in mol/L) in a saturated aqueous solution at equilibrium with its most stable crystalline form. In practice, around 85% of drugs have log S values ranging from −1 to −5, with almost none falling below −6. Empirically, the log S range of −1 to −5 for most drugs suggests a balance between the polarity required for adequate aqueous solubility and the hydrophobicity necessary for effective membrane permeability [78].

For the hybrids, the values of logS were between −6.4443 (for Do-De) and 0.497277 (for Ti-Za), while albicidin had a logS of −7.27913.

The number of rotatable bonds the hybrids possessed ranged from nine to fourteen, with albicidin having nine rotatable bonds, the same as Mi-No, Mi-Ci, Mi-Fi, and Mi-De.

The tPSA indicates a drug’s polarity, providing insights into its lipid solubility. This value increases with more polar groups in the drug’s structure. Functional groups containing nitrogen and oxygen atoms contribute to polarity, raising the tPSA value. Drugs with higher tPSA values tend to be less lipid-soluble and, as a result, are generally absorbed less efficiently and at a slower rate, with a more limited distribution than drugs with lower tPSA values [79]. Lower tPSA values (usually below 90 Å^2^) are indicators of blood–brain barrier crossing, while values greater than 140 Å^2^ generally indicate poor cell membrane permeability [80,81]. Albicidin had the highest tPSA value (285.74 Å^2^), with the hybrids’ values ranging from 208.25 Å^2^ to 282.5 Å^2^ for Ti-De. As expected, neither the hybrids nor albicidin was deemed a blood–brain barrier penetrator.

The span is a size descriptor representing the radius of the smallest sphere, centered at the molecule’s center of mass, fully enclosing all of its atoms [82]. The range for this descriptor was 13.6502 to 19.7615, with albicidin having 16.647. The hybrids with the closest spans to albicidin were Mi-Za and Mi-Fi (15.126 and 15.4229).

Considering these parameters, choosing the most similar hybrid to albicidin is difficult. However, the Do-Ba, Mi-Fi, and Te-Ba hybrids could be good candidates.

### 3.4. Molecular Docking

#### 3.4.1. Self-Docking of Albicidin

A self-docking step should be performed initially as a validation check to ensure that the software can accurately model and select the correct conformation of a ligand bound to the protein target. Self-docking involves using the native ligand from the crystal structure and attempting to dock it into the protein accurately. This step is essential whenever a crystal structure with a co-crystallized ligand is available.

The best results out of five runs were as follows: energy of −171.899 kcal/mol; RMSD of 2.36 Å; Rank Score of −26.846; Match Score of 39.746; FITTED Score of −33.2054. The energy measures the binding affinity, a lower energy indicating greater affinity of the ligand for the target. The Rank Score consists of a set of scoring functions that account for energy terms and other factors, all scaled to more accurately reflect observed binding free energies, with lower Rank Scores indicating better results. The Match Score evaluates how well the ligand fits within the active site, relying on the interaction sites generated by ProCESS. Higher scores indicate a better fit between the ligand and protein interaction sites. The FITTED Score combines the Rank Score and Match Score, offering an assessment of the ligand’s interaction with the active site from both an energetic and geometric perspective. It is regarded as the primary metric for ranking compounds.

The RMSD is the most widely used metric to quantify the similarity between two superimposed atomic coordinates [83]. The usual standard for the RMSD is below 2 Å. However, this value can be higher for very flexible molecules. Given that albicidin has nine rotatable bonds, a higher value for RMSD was expected. Some authors consider an RMSD between two and three acceptable [84]. Figure 1 illustrates that although the position of the docked ligand shows some deviation from the reference position, it maintains the correct orientation. Moreover, the amino acid and nucleotide residues interactions are similar between the docked and the crystallized albicidin, common interactions being with ALA67, ARG68, LYS447, ARG121, ILE74, DT14, DG15, DC18, and DA19 through either the same or different types of bonds. Due to the obtained value of 2.36 Å falling in the acceptable interval and the docked ligand interacting similarly with the binding pocket, we concluded that the program was suited for performing the docking for the hybrids.

#### 3.4.2. Docking of the Hybrids

Each of the 40 hybrids was docked using the binding site generated in the self-docking phase. The best FITTED Score by far was that of the Te-De hybrid (−47.7125), with the rest of the hybrids having scores ranging from −38.1989 (Te-Be) to −25.9024 (Te-Ne).

For Te-De, the interactions with the binding site were similar to those of albicidin, with the same Pi-Alkyl bonds with ALA67 and DG15 and other bonds with LYS65 (Pi-Alkyl for albicidin, Alkyl for Te-De), DT14 (Pi-Cation and Pi-Alkyl for albicidin, Conventional Hydrogen Bonds and Pi-Sigma for Te-De), ARG121 (Pi-Alkyl for albicidin, Metal–Acceptor for Te-De), and ASP82 (Carbon–Hydrogen Bond for albicidin, Conventional Hydrogen Bond for Te-De), as can be observed in Figure 2 and Figure 4. Additionally, Te-De interacted with Mg^2+^ through a fluorine atom, an indicator of a mechanism of action comparable to that of FQNs [13]. Interestingly, none of the other hybrids showed interactions with Mg^2+^ except for Do-Za, whose interaction was through the carbonylic oxygen atom in position 3 of doxycycline, not a structure of the FQN part (Figure S2 (part 5 of 20)). Moreover, it is worth mentioning that one of the two hybrids that interacted with Mg^2+^ was the one with the highest FITTED Score.

Between the scores predicted by the platform, the FITTED Score serves as the key indicator of optimal interaction with the active site. It incorporates the Match Score (reflecting how well the ligand fits within the active site) and the Rank Score (associated with the binding free energy). A lower FITTED Score suggests stronger binding affinity. However, it should be noted that this score does not necessarily correlate directly with the enzyme-inhibitory activity. Binding affinity refers to the change in free energy during the binding process; it measures the strength of the interaction between a ligand and a protein, often linked to the ligand’s potency [85]. It is essential to highlight that the mechanism of FQNs involves blocking DNA strand re-ligation by binding to the cleavage complexes at the enzyme–DNA interface. This process halts the catalytic cycle of DNA gyrase following DNA cleavage, increasing the accumulation of cleavage complexes [13]. Thus, stronger interactions with the enzyme could potentially lead to enhanced activity.

This study focused on designing TC-FQN hybrids and studying their mechanisms of action correlated with the interaction with DNA gyrase and the similarity to albicidin. Regarding the physicochemical profile, Do-Ba, Mi-Fi, and Te-Ba are likely the most similar to albicidin, as is also indicated by the fact that these three hybrids had FITTED Scores very similar to that of albicidin (−32.4995, −33.2309, and −34.9896 for the hybrids and −33.2054 for albicidin, respectively). Nevertheless, some hybrids had better FITTED Scores than albicidin, with Te-De being the clear winner at −47.7125. Five other hybrids had better scores than albicidin by a difference greater than 3 (Te-Be: −38.1989, Ti-De: −36.768, Do-Ci: −36.667, Do-De: −36.3935, and Ti-Ne: −36.3477).

The obtained results indicate a mechanism of action similar to that of FQNs, namely, the inhibition of DNA synthesis by binding to the cleavage complexes at the enzyme–DNA interface, preventing the re-ligation of DNA strands.

Sriram D. et al. (2007) evaluated the antimycobacterial activity of the hybrids synthesized by them. They hypothesized that an explanation for the enhanced activity observed for three compounds (compounds 4, 10, and 12) could be a dual mechanism of action [46].

Future studies could evaluate the inhibitory activity on the 30S ribosomal subunit to assess whether the mechanism of action of the hybrids could be a dual one. Targeting more than one bacterial enzyme/substructure could bring the benefit of a lower susceptibility to resistance development [86].

The ability to bind to DNA gyrase evaluated in this study could indicate a spectrum of activity similar to that of FQNs. Additionally, the possibility of an additional mechanism of action similar to that of TCs (considering the TC part of the hybrids) could support the hypothesis of a broad spectrum of activity for these hybrids. However, more in vitro and in vivo studies are needed to confirm this aspect.

Considering that profiles of all the hybrids were similar to that of albicidin, synthesizing them and evaluating their in vitro activity could prove valuable, especially since the synthesis method is quite simple and fast. The approach of antibiotic hybrids and the TC-FQN hybrids presented in this paper can potentially add significant value to the pool of active molecules used to combat antimicrobial resistance, a persistent global challenge.

## 4. Materials and Methods

The platforms and software we used were Biovia DRAW 2021, version 21.1.0.2363 [87] (used to design and process the hybrids’ structures), the Protein Data Bank (PDB) (https://www.rcsb.org/, accessed on 24 March 2024) [88,89] (to explore available co-crystallized structures of DNA gyrase with ligands), Biovia Discovery Studio 2024, version 24.1.0 [90] (to visualize and process the structures), and FORECASTER, version 6690 [91,92,93,94,95,96,97] (to compare the structures, assess the physicochemical profiles, and perform the molecular docking).

### 4.1. Designing the Hybrids and Selecting the Target

We designed a series of hybrids based on the experiments of Sriram D. et al. (2007) [46]. The structures were drawn with Biovia DRAW [87]. We selected FQNs approved for human use and only those with a nitrogen-containing moiety in position 7 (except for delafloxacin, which has the desired nitrogen-containing moiety at position 1). The presence of this type of moiety is a necessary condition for the Mannich reaction. The selected FQNs were balofloxacin, besifloxacin, ciprofloxacin, delafloxacin, finafloxacin, moxifloxacin, nemonoxacin, norfloxacin, sitafloxacin, and zabofloxacin, while the selected TCs were doxycycline, minocycline, tetracycline, and tigecycline. The two antibiotic components are linked at the amide group at C2 (for the TC) and the secondary or primary amine of the moiety in position 7 (primary amine in position 1 for delafloxacin) of the FQN through a methylene connector offered by formaldehyde.

We searched the Protein Data Bank (PDB) (https://www.rcsb.org/, accessed on 24 March 2024) [88,89] for structures of the DNA gyrase enzyme co-crystallized with ligands obtained by electron microscopy (EM) or X-ray diffraction (XRD). We chose to study the possible interaction of the hybrids with the DNA Gyrase of *E. Coli* (PDB-ID: 7Z9C; resolution: 3.06 Å) and to compare the hybrids with the co-crystallized ligand of this structure (albicidin). This molecule binds to DNA gyrase in a similar spot to FQNs [59].

### 4.2. Determining the Similarity Between the TC-FQN Hybrids and the Co-Crystallized Ligand

Using the “Chemical library cleaning” SELECT function of FORECASTER [92], we calculated the molecular similarity between the hybrids and albicidin using the MACCS fingerprint scheme and the Tanimoto coefficient as the similarity measure.

### 4.3. Comparing the Hybrids with the Co-Crystallized Ligand Regarding the Physicochemical Properties

To calculate the physicochemical parameters of the designed structures and the co-crystallized ligand (albicidin), we used the SMART function of FORECASTER [92,98].

### 4.4. Molecular Docking

#### 4.4.1. Self-Docking of Albicidin

This step was accomplished using the “Docking small molecule(s) to protein(s)” function of FORECASTER and the file 7z9c.pdb, downloaded from PDB. The PREPARE (Protein Rotamers Elaboration and Protonation based on Approximate Residue Energy), SMART, ProCESS (Protein Conformational Ensemble System Setup), and FITTED (Flexibility Induced Through Targeted Evolutionary Description) modules were selected [92,93,97,98,99].

First, the PREPARE function was used. This cleans up .pdb files (e.g., reconstructs missing side chains), then adds hydrogen atoms and optimizes their positions. Next, it produces protein and ligand files that are ready for use with the ProCESS molecular modelling program. The ProCESS program takes the protein files and prepares the necessary files for docking with FITTED. We selected the “Prepare for—Docking to rigid protein” option. The FITTED module docks small molecules to proteins, considering displaceable water molecules. The FITTED module was set to evaluate the RMSD and run.

Since the molecules have at least nine rotatable bonds, we set the Maximum Generations and Population to 500 for the FITTED parameters and set the number of runs to five.

#### 4.4.2. Docking of the Hybrids

For this step, we used the “Docking small molecule(s) to protein(s)” function of the FORECASTER platform and the binding pocket created in the self-dock phase. The functions used in this stage were CONVERT, SMART, and FITTED. CONVERT transforms 2D structures into 3D and adds hydrogens. SMART prepares the molecules for docking with FITTED. Biovia Discovery Studio v24.1.0.23298 [90] was used to visualize the structures for docking and self-docking.

## 5. Conclusions

This study designed and explored the mechanisms of action of the hypothetical TC-FQN hybrids, focusing on their interaction with DNA Gyrase and their similarity to albicidin. Do-Ba, Mi-Fi, and Te-Ba hybrids showed the closest physicochemical resemblance to albicidin and similar FITTED Scores. However, some hybrids, particularly Te-De, outperformed albicidin with better scores. The studied hybrids likely inhibit DNA synthesis by binding to enzyme–DNA complexes, similar to fluoroquinolones (FQNs). Although the 30S ribosomal subunit was not identified as a target, future studies could examine its role in a possible dual mechanism of action. These hybrids may offer broad-spectrum activity and reduce bacterial resistance development. Further in vitro and in vivo research is needed to confirm these findings. TC-FQN hybrids have the potential to contribute to addressing the fight against antibacterial resistance.

## Figures and Tables

**Table 1:** The makeup of the structures of the designed TC-FQN hybrids. The black dot represents the overlapping point where the two components assemble (the –CH_2_– connector).

TC Component	FQN Component	
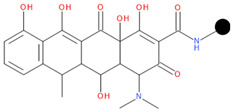	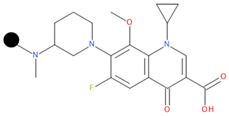	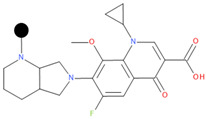
Doxycycline–CH_2_–	–CH_2_–Balofloxacin	–CH_2_–Moxifloxacin
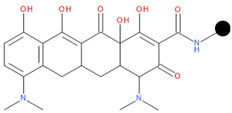	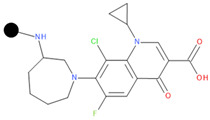	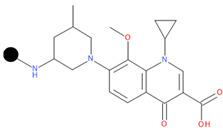
Minocycline–CH_2_–	–CH_2_–Besifloxacin	–CH_2_–Nemonoxacin
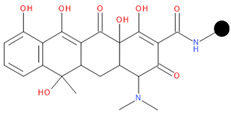	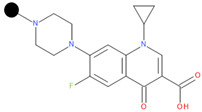	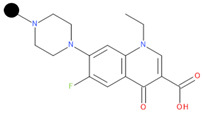
Tetracycline–CH_2_–	–CH_2_–Ciprofloxacin	–CH_2_–Norfloxacin
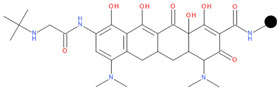	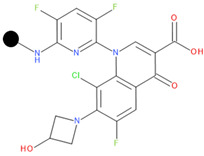	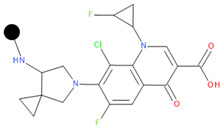
Tigecycline–CH_2_–	–CH_2_–Delafloxacin	–CH_2_–Sitafloxacin
	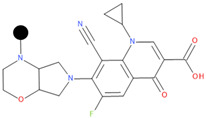	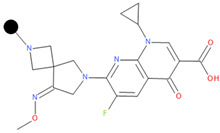
	–CH_2_–Finafloxacin	–CH_2_–Zabofloxacin

**Table 2:** Tanimoto coefficients obtained by comparing the hybrids with albicidin using the FORECASTER platform.

Hybrid Code	Tanimoto Coefficient	Hybrid Code	Tanimoto Coefficient	Hybrid Code	Tanimoto Coefficient	Hybrid Code	Tanimoto Coefficient
Do-Ba	0.67	Mi-Ba	0.67	Te-Ba	0.68	Ti-Ba	0.68
Do-Be	0.66	Mi-Be	0.65	Te-Be	0.66	Ti-Be	0.66
Do-Ci	0.66	Mi-Ci	0.66	Te-Ci	0.67	Ti-Ci	0.67
Do-De	0.66	Mi-De	0.65	Te-De	0.66	Ti-De	0.64
Do-Fi	0.63	Mi-Fi	0.63	Te-Fi	0.64	Ti-Fi	0.64
Do-Mo	0.64	Mi-Mo	0.64	Te-Mo	0.64	Ti-Mo	0.65
Do-Ne	0.67	Mi-Ne	0.67	Te-Ne	0.67	Ti-Ne	0.68
Do-No	0.67	Mi-No	0.66	Te-No	0.67	Ti-No	0.67
Do-Si	0.65	Mi-Si	0.64	Te-Si	0.65	Ti-Si	0.65
Do-Za	0.60	Mi-Za	0.59	Te-Za	0.60	Ti-Za	0.60

**Table 3:** Selection of obtained parameters of the designed hybrids and albicidin using the SMART function of the FORECASTER platform.

Hybrid Code	HBD	HBA	MW	logP	logS	RB	tPSA	BBB	Span
Do-Ba	9	12	847.894	7.96004	−4.32137	12	234.47	0	14.761
Do-Be	10	11	852.31	8.64016	−5.93672	11	225.24	0	13.6502
Do-Ci	9	11	789.814	6.92408	−4.25757	10	225.24	0	14.2762
Do-De	10	13	898.222	7.14079	−6.4443	10	270.39	0	13.9193
Do-Fi	9	12	856.861	6.78255	−4.02152	10	258.26	0	14.5525
Do-Mo	9	12	859.905	8.18711	−4.8601	11	234.47	0	14.1119
Do-Ne	10	12	829.904	7.71927	−3.79623	12	234.47	0	14.7355
Do-No	9	11	777.803	6.65226	−4.23877	10	225.24	0	14.5137
Do-Si	10	11	868.285	8.42228	−5.82351	11	225.24	0	14.1988
Do-Za	9	14	859.865	5.31606	−3.01163	11	247.36	0	14.4449
Mi-Ba	8	11	860.937	7.75452	−3.94298	11	217.48	0	14.9754
Mi-Be	9	10	865.353	8.43635	−5.61406	10	208.25	0	13.9213
Mi-Ci	8	10	802.857	6.7219	−3.91432	9	208.25	0	14.5806
Mi-De	9	12	911.265	6.94443	−6.16854	9	253.4	0	14.078
Mi-Fi	8	11	869.904	6.56169	−3.57005	9	241.27	0	15.4229
Mi-Mo	8	11	872.948	7.97204	−4.46624	10	217.48	0	14.85
Mi-Ne	9	11	842.947	7.50761	−3.40136	11	217.48	0	15.0102
Mi-No	8	10	790.846	6.45784	−3.90934	9	208.25	0	14.8218
Mi-Si	9	10	881.328	8.21306	−5.48178	10	208.25	0	14.3282
Mi-Za	8	13	872.908	5.08481	−2.37858	10	230.37	0	15.126
Te-Ba	9	12	847.894	6.7395	−3.49544	12	234.47	0	14.698
Te-Be	10	11	852.31	7.38854	−5.08594	11	225.24	0	13.6847
Te-Ci	9	11	789.814	5.76569	−3.49409	10	225.24	0	14.3242
Te-De	10	13	898.222	5.9824	−5.66162	10	270.39	0	13.9825
Te-Fi	9	12	856.861	5.59308	−3.28442	10	258.26	0	14.51
Te-Mo	9	12	859.905	6.93548	−4.1755	11	234.47	0	13.8736
Te-Ne	10	12	829.904	6.49873	−2.97309	12	234.47	0	14.7124
Te-No	9	11	777.803	5.52495	−3.49583	10	225.24	0	14.5488
Te-Si	10	11	868.285	7.17066	−4.97454	11	225.24	0	14.1618
Te-Za	9	14	859.865	4.18875	−2.24149	11	247.36	0	14.4696
Ti-Ba	10	13	989.112	8.20142	−0.609453	14	249.82	0	19.7149
Ti-Be	11	12	993.528	8.9158	−2.68174	13	240.59	0	18.6122
Ti-Ci	10	12	931.032	7.12409	−1.04944	12	240.59	0	18.8034
Ti-De	12	13	1040.45	7.36406	−3.02325	12	282.5	0	19.1446
Ti-Fi	10	13	998.079	6.9352	−0.559961	12	273.61	0	19.7615
Ti-Mo	10	13	1001.12	8.41774	−1.60355	13	249.82	0	18.7474
Ti-Ne	11	13	971.122	7.93612	−0.0193502	14	249.82	0	19.7203
Ti-No	10	12	919.021	6.85594	−1.10865	12	240.59	0	18.5602
Ti-Si	11	12	1009.50	8.67628	−2.50717	13	240.59	0	19.2606
Ti-Za	10	15	1001.08	5.37239	0.497277	13	262.71	0	19.2322
Albicidin	9	12	842.818	8.70223	−7.27913	9	285.74	0	16.647

HBD: number of hydrogen bond donors; HBA: number of hydrogen bond acceptors; MW: molecular weight (in g/mol); logP: computed logP developed specifically in FORECASTER; logS: logarithm of solubility; RB: number of rotatable bonds; tPSA: topological polar surface area (in angstroms^2^); BBB: blood–brain barrier permeator.

**Table 4:** Docking results of the hybrids in the binding site of *E. coli* gyrase holocomplex with 217 bp DNA obtained in the self-docking phase for albicidin.

No.	Hybrid Code	Energy (kcal/mol)	Rank Score	Match Score	FITTED Score
1.	Do-Ba	−46.8805	−25.9619	40.8598	−32.4995
2.	Do-Be	−55.3341	−29.0247	27.6213	−33.4442
3.	Do-Ci	−52.5709	−26.8659	61.2568	−36.667
4.	Do-De	−41.1101	−31.9846	27.5557	−36.3935
5.	Do-Fi	−59.1246	−29.9974	38.7332	−36.1947
6.	Do-Mo	−44.0383	−21.2354	42.2432	−27.9944
7.	Do-Ne	−52.2205	−21.4368	44.3933	−28.5397
8.	Do-No	−65.3521	−22.5224	39.6634	−28.8685
9.	Do-Si	−43.6101	−21.8714	44.1344	−28.9329
10.	Do-Za	−27.7438	−28.3422	28.6443	−32.9253
11.	Mi-Ba	−47.7369	−24.8899	33.7495	−30.2898
12.	Mi-Be	−50.6453	−23.5048	44.205	−30.5776
13.	Mi-Ci	−54.9941	−20.2415	37.5034	−26.2421
14.	Mi-De	−44.5589	−28.5292	31.7109	−33.6029
15.	Mi-Fi	−56.69	−27.7948	33.9761	−33.2309
16.	Mi-Mo	−55.2164	−27.4651	29.489	−32.1834
17.	Mi-Ne	−56.1481	−28.0855	34.6462	−33.6289
18.	Mi-No	−60.445	−21.1604	36.1175	−26.9392
19.	Mi-Si	−41.4239	−27.8282	52.2429	−36.187
20.	Mi-Za	−33.6735	−27.0834	28.1093	−31.5809
21.	Te-Ba	−52.1768	−29.2146	36.094	−34.9896
22.	Te-Be	−58.9201	−30.1819	50.1065	−38.1989
23.	Te-Ci	−58.1497	−22.665	36.0531	−28.4335
24.	Te-De	−55.2032	−41.5255	38.6688	−47.7125
25.	Te-Fi	−51.9233	−23.225	41.78	−29.9098
26.	Te-Mo	−45.6765	−21.5231	37.5876	−27.5371
27.	Te-Ne	−56.0786	−19.223	41.7466	−25.9024
28.	Te-No	−61.5205	−20.0941	38.623	−26.2737
29.	Te-Si	−44.2699	−23.2726	36.39	−29.095
30.	Te-Za	−25.535	−23.5952	31.9301	−28.704
31.	Ti-Ba	−77.9709	−21.1048	37.5492	−27.1127
32.	Ti-Be	−78.3624	−25.9144	37.413	−31.9005
33.	Ti-Ci	−93.1157	−26.1696	38	−32.2496
34.	Ti-De	−58.2974	−30.7778	37.439	−36.768
35.	Ti-Fi	−81.4413	−25.177	41.3032	−31.7855
36.	Ti-Mo	−68.6103	−27.8297	36.9597	−33.7432
37.	Ti-Ne	−98.4678	−30.1133	38.9649	−36.3477
38.	Ti-No	−97.8969	−27.2614	37.6273	−33.2818
39.	Ti-Si	−82.2259	−26.0071	38.5462	−32.1745
40.	Ti-Za	−63.2206	−28.8194	37.9827	−34.8967
-	Albicidin	−171.899	−26.846	39.746	−33.2054

## Data Availability

Data is contained within the article and Supplementary Material.

## Supplementary Materials

The following supporting information can be downloaded at https://www.mdpi.com/article/10.3390/ph17111540/s1, Table S1: Obtained parameters of the designed hybrids and albicidin using the SMART function of the FORECASTER platform, Figure S1: The conformation of the hybrids (yellow) in the pocket of the *E. coli* DNA gyrase enzyme (red)–DNA (blue) complex, Figure S2: Interactions of the hybrids with the binding pocket of *E. coli* gyrase holocomplex with 217 bp DNA obtained in the self-docking phase for albicidin (in 3D (left) and 2D (right)).
