# Supplementary material for: In Silico Study of the Potential Inhibitory Effects on Escherichia coli DNA Gyrase of Some Hypothetical Fluoroquinolone–Tetracycline Hybrids
Source: Pharmaceuticals (Basel). 2024 Nov 16;17(11):1540. doi: 10.3390/ph17111540 (PMC11597511; doi:10.3390/ph17111540)
Supplement: Supplementary file 1 [file pharmaceuticals-17-01540-s001.zip › pharmaceuticals-3287336-supplementary.pdf]

pharmaceuticals

Table S1. (part 1 of 5). Obtained parameters of the designed hybrids and albicidin using the SMART function of the FORECASTER platform.

Hybrid code	2D or 3D	MW	NC	HBD	HBA	RB	Rgs	IC	#O	#N	#S	Het	Hvy
Do-Ba	2D	847.894	2	9	12	12	8	0	12	5	0	17	61
Do-Be	2D	852.31	2	10	11	11	8	0	11	5	0	16	60
Do-Ci	2D	789.814	2	9	11	10	8	0	11	5	0	16	57
Do-De	2D	898.222	1	10	13	10	8	1	12	6	0	18	63
Do-Fi	2D	856.861	2	9	12	10	9	0	12	6	0	18	62
Do-Mo	2D	859.905	2	9	12	11	10	0	12	5	0	17	62
Do-Ne	2D	829.904	2	10	12	12	8	0	12	5	0	17	60
Do-No	2D	777.803	2	9	11	10	7	0	11	5	0	16	56
Do-Si	2D	868.285	2	10	11	11	9	0	11	5	0	16	61
Do-Za	2D	859.865	2	9	14	11	9	1	12	7	0	19	62
Mi-Ba	2D	860.937	2	8	11	11	9	0	11	6	0	17	62
Mi-Be	2D	865.353	2	9	10	10	8	0	10	6	0	16	61
Mi-Ci	2D	802.857	2	8	10	9	8	0	10	6	0	16	58
Mi-De	2D	911.265	1	9	12	9	8	1	11	7	0	18	64
Mi-Fi	2D	869.904	2	8	11	9	9	0	11	7	0	18	63
Mi-Mo	2D	872.948	2	8	11	10	10	0	11	6	0	17	63
Mi-Ne	2D	842.947	2	9	11	11	8	0	11	6	0	17	61
Mi-No	2D	790.846	2	8	10	9	7	0	10	6	0	16	57
Mi-Si	2D	881.328	2	9	10	10	9	0	10	6	0	16	62
Mi-Za	2D	872.908	2	8	13	10	9	1	11	8	0	19	63
Te-Ba	2D	847.894	2	9	12	12	9	0	12	5	0	17	61
Te-Be	2D	852.31	2	10	11	11	8	0	11	5	0	16	60
Te-Ci	2D	789.814	2	9	11	10	9	0	11	5	0	16	57
Te-De	2D	898.222	1	10	13	10	8	1	12	6	0	18	63
Te-Fi	2D	856.861	2	9	12	10	10	0	12	6	0	18	62
Te-Mo	2D	859.905	2	9	12	11	10	0	12	5	0	17	62
Te-Ne	2D	829.904	2	10	12	12	9	0	12	5	0	17	60
Te-No	2D	777.803	2	9	11	10	8	0	11	5	0	16	56
Te-Si	2D	868.285	2	10	11	11	9	0	11	5	0	16	61
Te-Za	2D	859.865	2	9	14	11	9	1	12	7	0	19	62
Ti-Ba	2D	989.112	2	10	13	14	9	0	12	8	0	20	71
Ti-Be	2D	993.528	2	11	12	13	8	0	11	8	0	19	70
Ti-Ci	2D	931.032	2	10	12	12	8	0	11	8	0	19	67
Ti-De	2D	1040.45	2	12	13	12	8	1	12	9	0	21	73
Ti-Fi	2D	998.079	2	10	13	12	9	0	12	9	0	21	72
Ti-Mo	2D	1001.12	2	10	13	13	9	0	12	8	0	20	72
Ti-Ne	2D	971.122	2	11	13	14	9	0	12	8	0	20	70
Ti-No	2D	919.021	2	10	12	12	7	0	11	8	0	19	66
Ti-Si	2D	1009.50	2	11	12	13	9	0	11	8	0	19	71
Ti-Za	2D	1001.08	2	10	15	13	9	1	12	10	0	22	72
Albicidin	2D	842.818	0	9	12	9	5	0	12	6	0	18	62

MW: molecular weight (in g/mol); NC: net molecular charge; HBD: number of hydrogen bond donors; HBA: number of hydrogen bond acceptors; RB: number of rotatable bonds; Rgs: number of rings; IC: number of ionizable centers; #O, #N, #S: number of oxygen, nitrogen, and sulphur atoms; Het: number of heteroatoms (atoms other than C, H); Hvy: number of heavy atoms (atoms other than H).

Table S1. (part 2 of 5). Obtained parameters of the designed hybrids and albicidin using the SMART function of the FORECASTER platform.

Hybrid code	Acc	tPSA	logP	Fsp3	SCr	logS	SC	AR	AP	#X
Do-Ba	0	234.47	7.96004	0.465116	0.162791	-4.32137	7	1	0.108108	1
Do-Be	0	225.24	8.64016	0.452381	0.166667	-5.93672	7	2	0.11215	2
Do-Ci	0	225.24	6.92408	0.425	0.15	-4.25757	6	2	0.118812	1
Do-De	0	270.39	7.14079	0.317073	0.146341	-6.4443	6	3	0.18	4
Do-Fi	0	258.26	6.78255	0.44186	0.186047	-4.02152	8	2	0.11215	1
Do-Mo	0	234.47	8.18711	0.477273	0.181818	-4.8601	8	3	0.107143	1
Do-Ne	0	234.47	7.71927	0.465116	0.186047	-3.79623	8	1	0.108108	0
Do-No	0	225.24	6.65226	0.410256	0.153846	-4.23877	6	2	0.12	1
Do-Si	0	225.24	8.42228	0.452381	0.214286	-5.82351	8	2	0.114286	3
Do-Za	0	247.36	5.31606	0.452381	0.142857	-3.01163	6	2	0.111111	1
Mi-Ba	0	217.48	7.75452	0.477273	0.113636	-3.94298	5	1	0.104348	1
Mi-Be	0	208.25	8.43635	0.465116	0.116279	-5.61406	5	2	0.108108	2
Mi-Ci	0	208.25	6.7219	0.439024	0.097561	-3.91432	4	2	0.114286	1
Mi-De	0	253.4	6.94443	0.333333	0.0952381	-6.16854	4	3	0.173077	4
Mi-Fi	0	241.27	6.56169	0.454545	0.136364	-3.57005	6	2	0.108108	1
Mi-Mo	0	217.48	7.97204	0.488889	0.133333	-4.46624	6	3	0.103448	1
Mi-Ne	0	217.48	7.50761	0.477273	0.136364	-3.40136	6	1	0.104348	0
Mi-No	0	208.25	6.45784	0.425	0.1	-3.90934	4	2	0.115385	1
Mi-Si	0	208.25	8.21306	0.465116	0.162791	-5.48178	6	2	0.110092	3
Mi-Za	0	230.37	5.08481	0.465116	0.0930233	-2.37858	4	2	0.107143	1
Te-Ba	0	234.47	6.7395	0.465116	0.139535	-3.49544	6	1	0.108108	1
Te-Be	0	225.24	7.38854	0.452381	0.142857	-5.08594	6	2	0.11215	2
Te-Ci	0	225.24	5.76569	0.425	0.125	-3.49409	5	1	0.118812	1
Te-De	0	270.39	5.9824	0.317073	0.121951	-5.66162	5	3	0.18	4
Te-Fi	0	258.26	5.59308	0.44186	0.162791	-3.28442	7	2	0.11215	1
Te-Mo	0	234.47	6.93548	0.477273	0.159091	-4.1755	7	2	0.107143	1
Te-Ne	0	234.47	6.49873	0.465116	0.162791	-2.97309	7	1	0.108108	0
Te-No	0	225.24	5.52495	0.410256	0.128205	-3.49583	5	1	0.12	1
Te-Si	0	225.24	7.17066	0.452381	0.190476	-4.97454	7	2	0.114286	3
Te-Za	0	247.36	4.18875	0.452381	0.119048	-2.24149	5	2	0.111111	1
Ti-Ba	0	249.82	8.20142	0.52	0.1	-0.609453	5	1	0.0882353	1
Ti-Be	0	240.59	8.9158	0.510204	0.102041	-2.68174	5	2	0.0909091	2
Ti-Ci	0	240.59	7.12409	0.489362	0.0851064	-1.04944	4	1	0.0952381	1
Ti-De	0	282.5	7.36406	0.395833	0.0833333	-3.02325	4	3	0.142857	4
Ti-Fi	0	273.61	6.9352	0.5	0.12	-0.559961	6	2	0.0909091	1
Ti-Mo	0	249.82	8.41774	0.529412	0.117647	-1.60355	6	2	0.0875912	1
Ti-Ne	0	249.82	7.93612	0.52	0.12	-0.0193502	6	1	0.0882353	0
Ti-No	0	240.59	6.85594	0.478261	0.0869565	-1.10865	4	1	0.096	1
Ti-Si	0	240.59	8.67628	0.510204	0.142857	-2.50717	6	2	0.0923077	3
Ti-Za	0	262.71	5.37239	0.510204	0.0816327	0.497277	4	2	0.0902256	1
Albicidin	1	285.74	8.70223	0.113636	0.0227273	-7.27913	1	5	0.3	0

Acc: number of Michael acceptor groups; tPSA: topological polar surface area (in angstroms^2); logP: computed logP developed specifically in FORECASTER; Fsp3: fraction of sp3 carbons; SCr: ratio of stereogenic centers over the number of heavy atoms; logS: logarithm of solubility; SC: number of stereogenic centers; AR: number of aromatic rings; AP: aromatic proportion; #X: number of halogen atoms.

Table S1. (part 3 of 5). Obtained parameters of the designed hybrids and albicidin using the SMART function of the FORECASTER platform.

Hybrid code	MGV	mVSA	pSASA	nSASA	molDe	molPo	BBB	MolSf	MolEl
Do-Ba	596.05	1734.12	380.407	750.294	1.42252	0.169117	0	3.27484	2.50477
Do-Be	588.33	1699.12	420.502	637.543	1.44869	0.177732	0	3.45744	2.50883
Do-Ci	547.91	1599.25	389.137	681.563	1.4415	0.172507	0	3.23989	2.51323
Do-De	578.56	1689.94	516.14	599.535	1.55251	0.270956	0	3.30431	2.58024
Do-Fi	586.57	1716.13	428.508	753.385	1.4608	0.261441	0	3.11636	2.52249
Do-Mo	599.28	1750.82	382.572	755.924	1.4349	0.166594	0	3.30804	2.50434
Do-Ne	594.29	1722.41	381.856	758.494	1.39646	0.164082	0	3.24468	2.49005
Do-No	544.68	1582.80	389.582	664.586	1.428	0.173813	0	3.22181	2.51321
Do-Si	579.23	1689.14	456.817	626.273	1.49903	0.183474	0	3.37157	2.52956
Do-Za	586.76	1719.35	391.23	715.873	1.46545	0.172216	0	3.37284	2.52583
Mi-Ba	614.25	1780.64	368.821	806.287	1.40161	0.160797	0	3.25067	2.49262
Mi-Be	606.53	1745.65	407.867	693.296	1.42673	0.169099	0	3.42774	2.49607
Mi-Ci	566.11	1646.03	379.069	736.509	1.4182	0.163231	0	3.21336	2.49961
Mi-De	596.76	1736.47	505.216	656.53	1.52702	0.187417	0	3.27632	2.564
Mi-Fi	604.77	1761.39	418.95	794.266	1.43841	0.252168	0	3.13535	2.509
Mi-Mo	617.48	1796.08	369.734	808.702	1.41373	0.158672	0	3.3152	2.49231
Mi-Ne	612.49	1767.92	371.941	812.758	1.37626	0.155798	0	3.22091	2.47838
Mi-No	562.88	1629.58	379.396	718.588	1.405	0.246698	0	3.19655	2.49944
Mi-Si	597.43	1735.67	444.849	684.601	1.4752	0.174487	0	3.34009	2.51587
Mi-Za	604.96	1764.61	386.739	812.734	1.44292	0.163318	0	3.25216	2.51267
Te-Ba	596.05	1734.12	403.866	728.107	1.42252	0.169099	0	3.27291	2.50465
Te-Be	588.33	1699.12	443.858	617.735	1.44869	0.177582	0	3.45016	2.50872
Te-Ci	547.91	1598.23	411.159	660.216	1.4415	0.172467	0	3.23966	2.51314
Te-De	578.56	1689.94	540.116	578.077	1.55251	0.197702	0	3.30361	2.58019
Te-Fi	586.57	1714.86	449.496	715.134	1.4608	0.172757	0	3.16627	2.52246
Te-Mo	599.28	1749.55	403.211	719.938	1.4349	0.166715	0	3.36751	2.50427
Te-Ne	594.29	1722.41	405.037	735.439	1.39646	0.164088	0	3.24486	2.48995
Te-No	544.68	1581.78	412.923	641.024	1.428	0.173697	0	3.22225	2.5131
Te-Si	579.23	1689.14	482.386	605.893	1.49903	0.18356	0	3.36711	2.5295
Te-Za	586.76	1719.35	413.797	693.219	1.46545	0.172167	0	3.37029	2.52571
Ti-Ba	720.32	2073.32	412.839	968.085	1.37316	0.157851	0	3.21639	2.48265
Ti-Be	712.6	2038.08	451.359	855.635	1.39423	0.164819	0	3.36538	2.48541
Ti-Ci	672.18	1937.44	420.083	899.793	1.38509	0.159875	0	3.18441	2.48771
Ti-De	704.98	2035.90	561.325	812.489	1.47585	0.183611	0	3.2468	2.53881
Ti-Fi	710.84	2055.08	470.936	944.394	1.40408	0.161087	0	3.13877	2.4951
Ti-Mo	723.55	2089.77	411.162	962.977	1.38363	0.156182	0	3.30209	2.48256
Ti-Ne	718.56	2061.62	410.773	977.529	1.35148	0.153878	0	3.19508	2.47063
Ti-No	668.95	1920.99	420.605	880.992	1.37383	0.232837	0	3.17192	2.48744
Ti-Si	703.5	2028.09	490.934	843.964	1.43497	0.169662	0	3.29344	2.5017
Ti-Za	711.03	2058.30	422.171	931.773	1.40793	0.160295	0	3.29812	2.49931
Albicidin	603.64	1719.09	447.812	811.379	1.39623	0.173233	0	3.01626	2.53094

MGV: McGowan molecular volume (in mL/mol); mVSA: molecular van der Waals surface area (in angstroms^2); pSASA: polar solvent accessible surface area (in angstroms^2); nSASA: nonpolar solvent accessible surface area (in angstroms^2); molDe: molecular density (in g/mL); molPo: molecular polarity; BBB: blood-brain barrier permeator; MolSf: molecular softness; MolEl: molecular electronegativity.

Table S1. (part 4 of 5). Obtained parameters of the designed hybrids and albicidin using the SMART function of the FORECASTER platform.

Hybrid code	O-	N+	Hardn	Polz	3DI	Radius	Diam	Shape
Do-Ba	0	2	12.8248	0.866113	587.256	15.24	29.3157	0.923607
Do-Be	0	2	12.7617	0.883951	503.323	14.0175	27.2468	0.943773
Do-Ci	0	2	12.8064	0.870837	507.351	14.3861	28.0651	0.950844
Do-De	0	1	12.8261	0.884497	483.417	13.8523	26.5137	0.914035
Do-Fi	0	2	12.7931	0.87405	551.508	14.7748	28.2803	0.914095
Do-Mo	0	2	12.8089	0.869479	560.241	14.3974	27.4133	0.904045
Do-Ne	0	2	12.7881	0.869387	591.198	15.2851	29.2555	0.913986
Do-No	0	2	12.824	0.867113	495.061	14.4188	28.0885	0.948047
Do-Si	0	2	12.7848	0.884624	519.313	14.6532	28.2693	0.929218
Do-Za	0	2	12.8209	0.867281	536.52	14.7837	28.6414	0.937363
Mi-Ba	0	2	12.81	0.86651	629.37	15.6075	29.5392	0.892626
Mi-Be	0	2	12.7486	0.883721	540.773	14.0344	27.2355	0.940624
Mi-Ci	0	2	12.7909	0.871092	545.724	14.3644	28.0653	0.953808
Mi-De	0	1	12.8097	0.88423	520.687	14.0411	26.8585	0.912849
Mi-Fi	0	2	12.7789	0.874176	610.775	15.7898	30.7004	0.944319
Mi-Mo	0	2	12.7947	0.869758	610.642	15.0254	29.3954	0.956383
Mi-Ne	0	2	12.7746	0.869671	633.567	15.5233	29.4565	0.897564
Mi-No	0	2	12.8077	0.867514	533.005	14.3867	28.0818	0.951931
Mi-Si	0	2	12.7706	0.884364	557.555	14.6264	28.2449	0.931093
Mi-Za	0	2	12.8059	0.867647	602.036	15.7865	29.7433	0.884094
Te-Ba	0	2	12.8248	0.866113	585.891	15.1853	29.2564	0.926628
Te-Be	0	2	12.7617	0.883951	502.22	14.0152	27.2111	0.941543
Te-Ci	0	2	12.8064	0.870837	505.796	14.3648	28.0543	0.952996
Te-De	0	1	12.8261	0.884497	482.076	13.8196	26.4403	0.913251
Te-Fi	0	2	12.7931	0.87405	547.29	14.7452	28.0971	0.905514
Te-Mo	0	2	12.8089	0.869479	547.884	14.2243	26.9892	0.897404
Te-Ne	0	2	12.7881	0.869387	589.506	15.2331	29.2082	0.917411
Te-No	0	2	12.824	0.867113	493.669	14.3944	28.0863	0.951197
Te-Si	0	2	12.7848	0.884624	517.998	14.6266	28.2235	0.929598
Te-Za	0	2	12.8209	0.867281	535.359	14.6862	28.6243	0.949062
Ti-Ba	0	2	12.8265	0.860311	888.924	18.7783	37.135	0.977553
Ti-Be	0	2	12.7754	0.874595	785.371	17.4082	34.7498	0.996173
Ti-Ci	0	2	12.8119	0.863637	788.792	18.2102	35.8587	0.969158
Ti-De	0	2	12.8338	0.87286	761.566	17.4813	34.2613	0.959878
Ti-Fi	0	2	12.8009	0.866569	839.255	18.4557	34.9946	0.89614
Ti-Mo	0	2	12.8135	0.863106	835.586	17.6071	33.525	0.904065
Ti-Ne	0	2	12.7966	0.862984	893.814	18.7538	37.0677	0.976543
Ti-No	0	2	12.8261	0.860601	773.945	18.1917	35.843	0.970294
Ti-Si	0	2	12.7943	0.874995	804.784	18.1453	35.8919	0.978023
Ti-Za	0	2	12.8234	0.861129	824.586	18.4664	36.1965	0.960133
Albicidin	0	0	12.6712	0.897948	590.489	16.9717	30.0634	0.771382

O-: number of negatively charged oxygen-containing groups; N+: number of positively charged nitrogen-containing groups; Hardn: molecular hardness; Polz: molecular polarizability; 3DI: 3D-Wiener index; Radius: geometric radius; Diam: geometric diameter; Shape: geometrical shape coefficient.

Table S1. (part 5 of 5). Obtained parameters of the designed hybrids and albicidin using the SMART function of the FORECASTER platform.

Hybrid code	Span	GyrR	Oval	Glob	Dipole	BA	QED
Do-Ba	14.761	8.87012	137.997	0.00724656	7.79565	211	0.0911969
Do-Be	13.6502	7.9341	135.212	0.0073958	9.35876	211	0.0963261
Do-Ci	14.2762	8.41723	127.264	0.00785768	7.37536	211	0.0886852
Do-De	13.9193	7.84125	134.481	0.00743599	31.0605	211	0.0717028
Do-Fi	14.5525	8.46163	136.565	0.00732251	42.8579	211	0.0885075
Do-Mo	14.1119	8.24081	139.326	0.00717744	17.723	211	0.0656017
Do-Ne	14.7355	8.93355	137.065	0.00729579	6.84651	211	0.0720592
Do-No	14.5137	8.32879	125.955	0.00793934	8.07102	211	0.0916906
Do-Si	14.1988	8.36912	134.418	0.00743951	16.1073	211	0.0965159
Do-Za	14.4449	8.35326	136.821	0.0073088	7.36481	211	0.102661
Mi-Ba	14.9754	9.12751	141.699	0.00705721	10.2522	211	0.100647
Mi-Be	13.9213	8.17482	138.914	0.00719868	11.4289	211	0.104191
Mi-Ci	14.5806	8.66936	130.987	0.00763437	9.26151	211	0.125595
Mi-De	14.078	8.10296	138.184	0.00723675	30.4589	211	0.0781897
Mi-Fi	15.4229	9.19248	140.167	0.00713436	34.2015	211	0.125546
Mi-Mo	14.85	8.78649	142.927	0.00699657	17.5375	211	0.0914155
Mi-Ne	15.0102	9.18851	140.687	0.00710798	9.48812	211	0.0784755
Mi-No	14.8218	8.57872	129.678	0.00771144	10.9123	211	0.130152
Mi-Si	14.3282	8.61314	138.12	0.00724008	19.079	211	0.104542
Mi-Za	15.126	9.00413	140.423	0.00712134	13.9262	211	0.115181
Te-Ba	14.698	8.89476	137.997	0.00724656	8.3965	211	0.099584
Te-Be	13.6847	7.96079	135.212	0.0073958	10.016	211	0.100378
Te-Ci	14.3242	8.44145	127.183	0.00786267	7.54301	211	0.129523
Te-De	13.9825	7.86826	134.481	0.00743599	26.4898	211	0.0829842
Te-Fi	14.51	8.44814	136.464	0.00732792	16.3156	211	0.132563
Te-Mo	13.8736	8.08922	139.225	0.00718263	16.1131	211	0.104672
Te-Ne	14.7124	8.95262	137.065	0.00729579	6.41869	211	0.0803747
Te-No	14.5488	8.35624	125.874	0.00794444	8.01915	211	0.106169
Te-Si	14.1618	8.39089	134.418	0.00743951	15.1333	211	0.101954
Te-Za	14.4696	8.38333	136.821	0.0073088	7.23394	211	0.113702
Ti-Ba	19.7149	10.6384	164.99	0.00606099	19.97	211	0.0790862
Ti-Be	18.6122	9.69502	162.185	0.0061658	23.459	211	0.0843829
Ti-Ci	18.8034	10.1775	154.176	0.00648608	19.048	211	0.0943018
Ti-De	19.1446	9.68801	162.012	0.00617238	39.1602	211	0.0624708
Ti-Fi	19.7615	10.1726	163.538	0.00611478	28.5075	211	0.0967505
Ti-Mo	18.7474	9.7671	166.299	0.00601328	28.0962	211	0.0847929
Ti-Ne	19.7203	10.689	164.058	0.00609539	16.9075	211	0.0626953
Ti-No	18.5602	10.0864	152.867	0.00654163	39.3334	211	0.076488
Ti-Si	19.2606	10.1663	161.391	0.00619615	30.5306	211	0.0845489
Ti-Za	19.2322	10.0969	163.794	0.00610522	20.6419	211	0.089357
Albicidin	16.647	9.67416	136.8	0.00730992	11.3004	0	0.0629953

Span: span; GyrR: radius of gyration; Oval: ovality index; Glob: globularity factor; Dipole: dipole moment; BA: Badapple pscore (PAINS) high: >300; medium >100; QED: Quantitative Estimate of Druglikeness; the higher the score, the more likely that the compound will be a drug-like molecule.

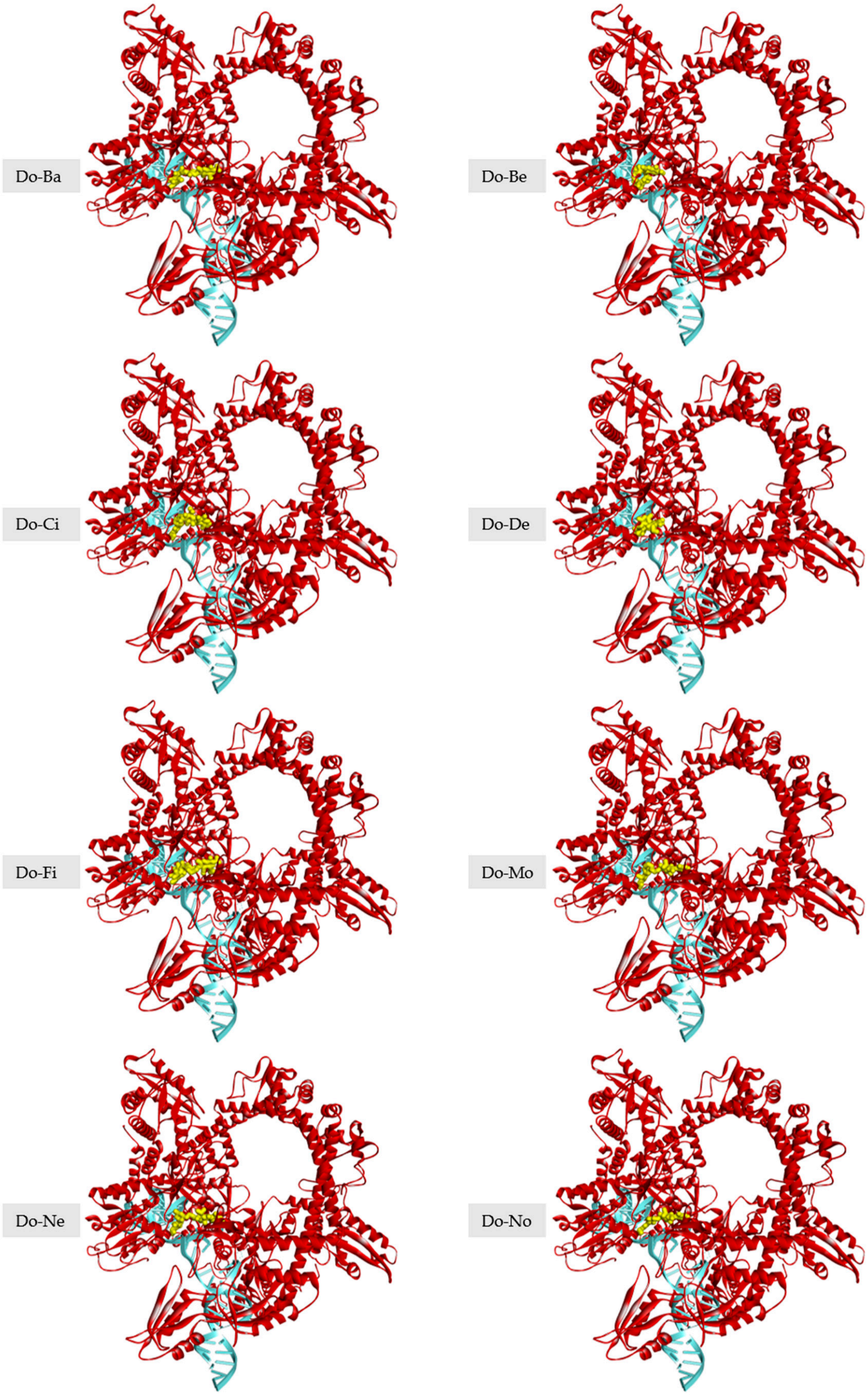

Figure S1. (part 1 of 5). The conformation of the hybrids (yellow) in the pocket of the *E. coli* DNA gyrase enzyme (red)–DNA (blue) complex.

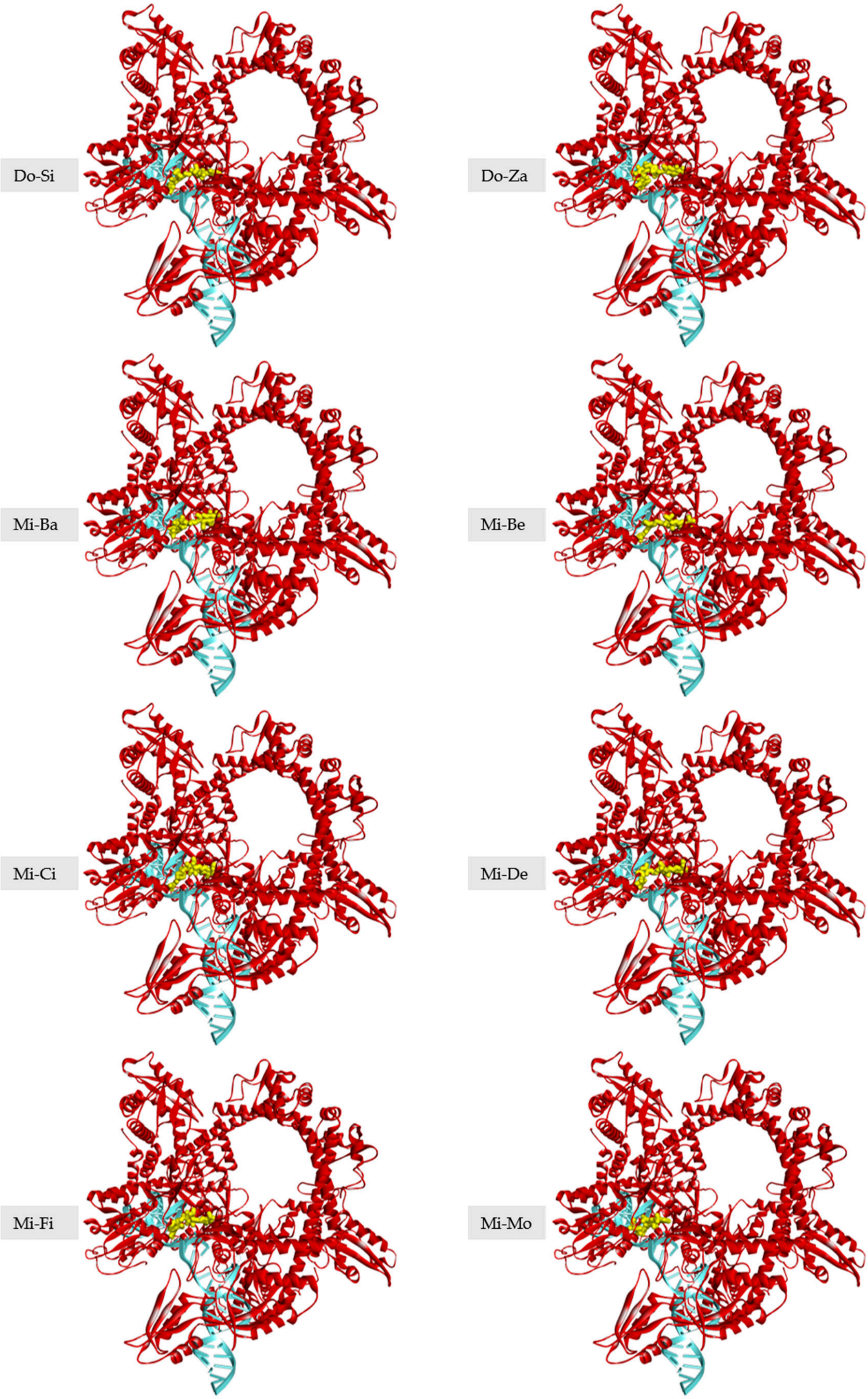

Figure S1. (part 2 of 5). The conformation of the hybrids (yellow) in the pocket of the *E. coli* DNA gyrase enzyme (red)–DNA (blue) complex.

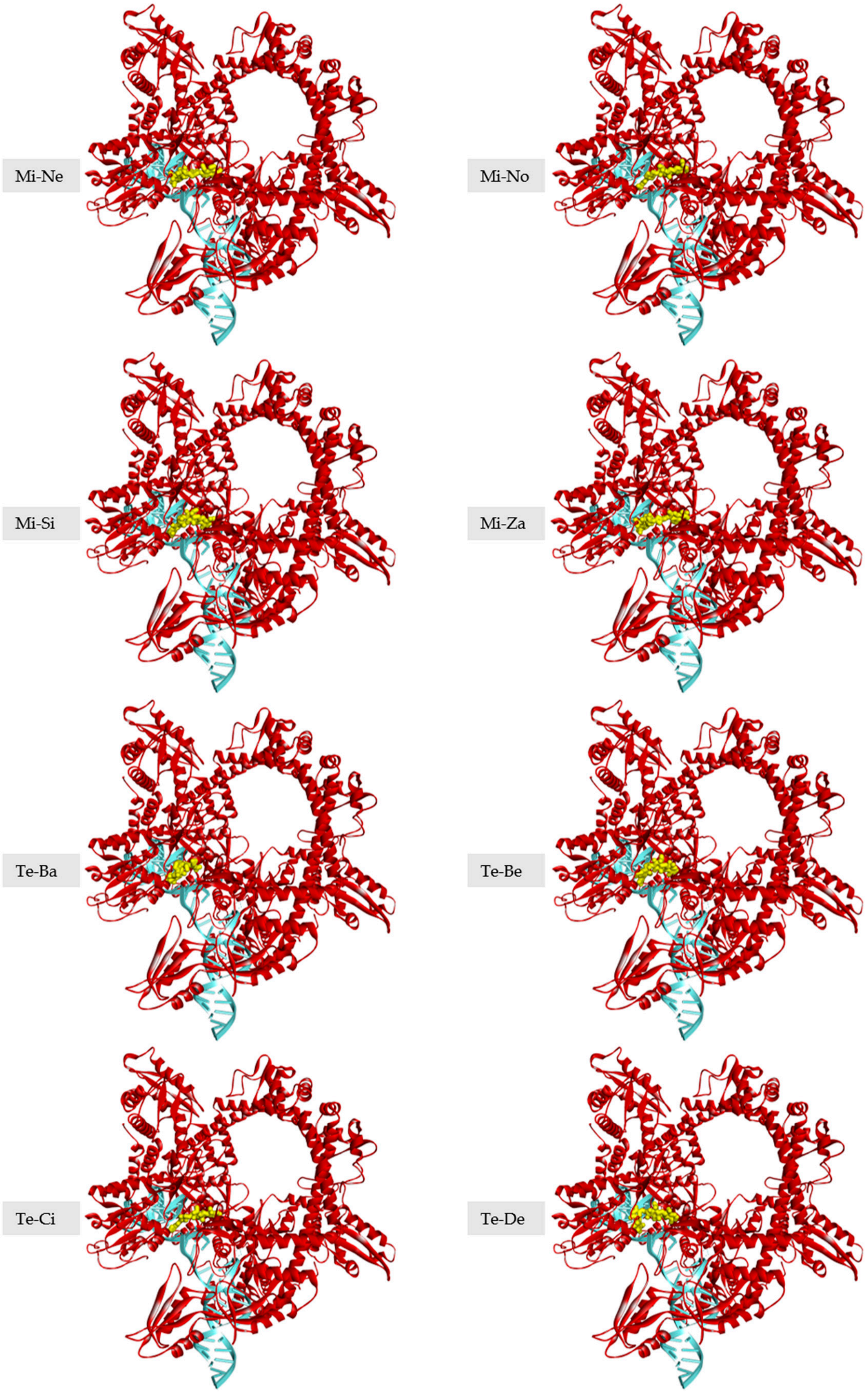

Figure S1. (part 3 of 5). The conformation of the hybrids (yellow) in the pocket of the *E. coli* DNA gyrase enzyme (red)–DNA (blue) complex.

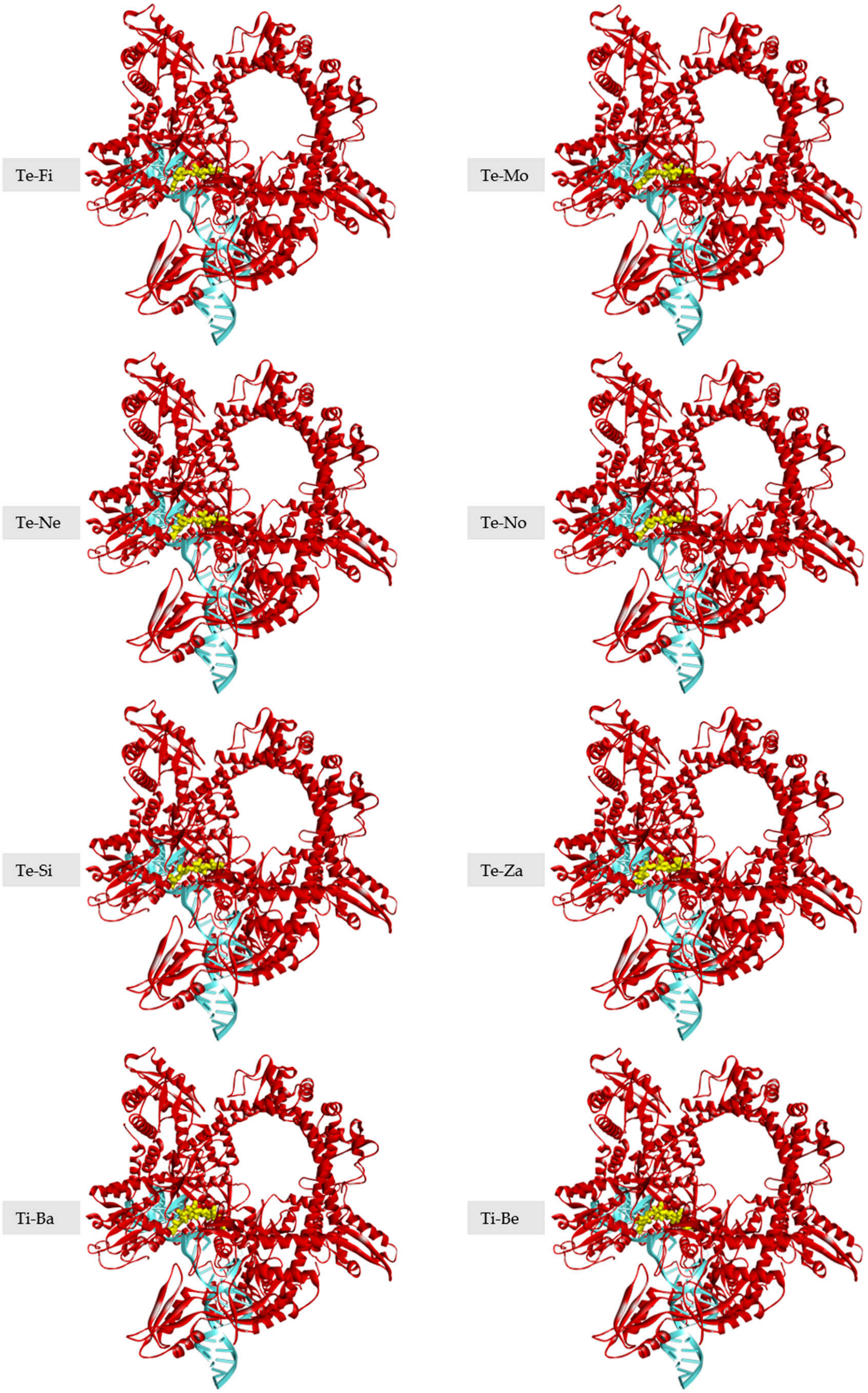

Figure S1. (part 4 of 5). The conformation of the hybrids (yellow) in the pocket of the *E. coli* DNA gyrase enzyme (red)–DNA (blue) complex.

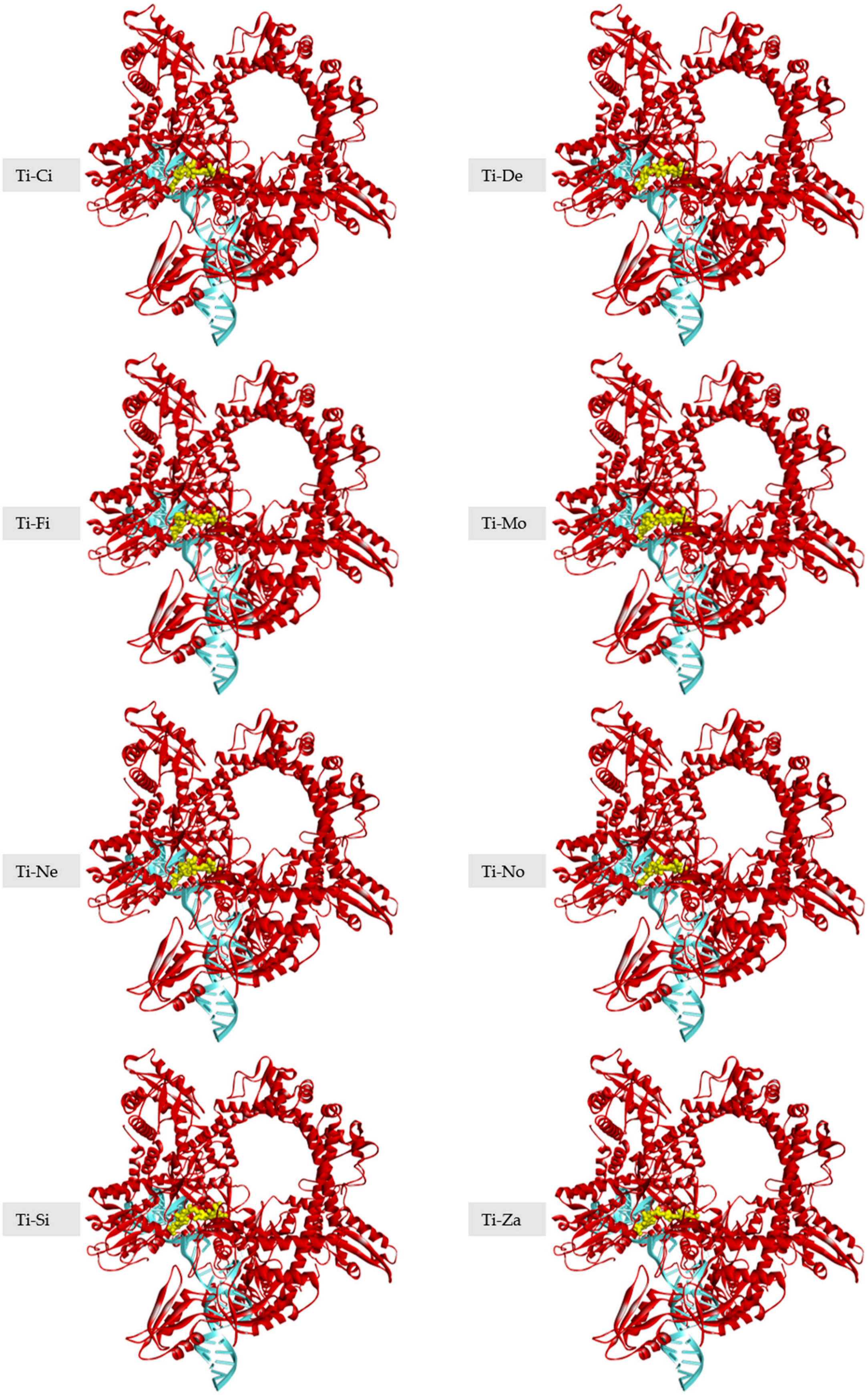

Figure S1. (part 5 of 5). The conformation of the hybrids (yellow) in the pocket of the *E. coli* DNA gyrase enzyme (red)–DNA (blue) complex.

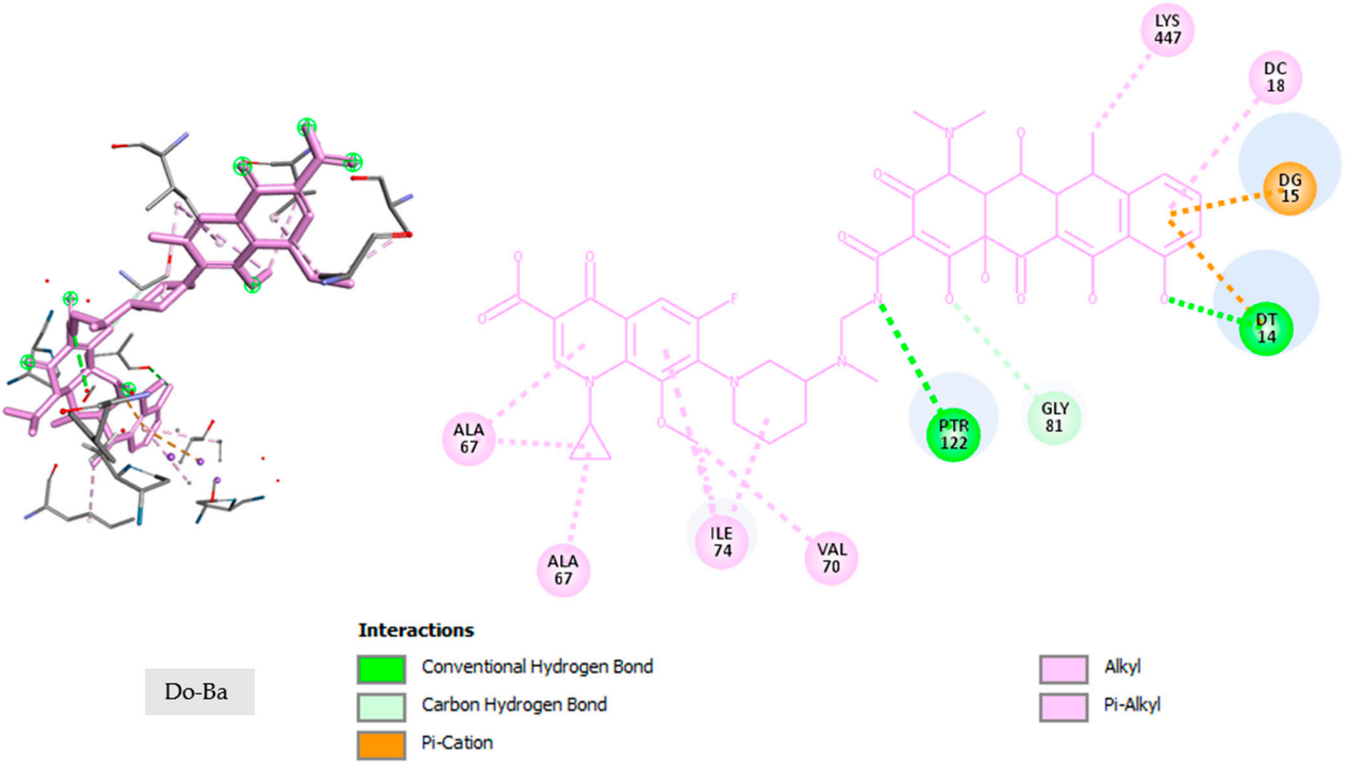

Do-Ba

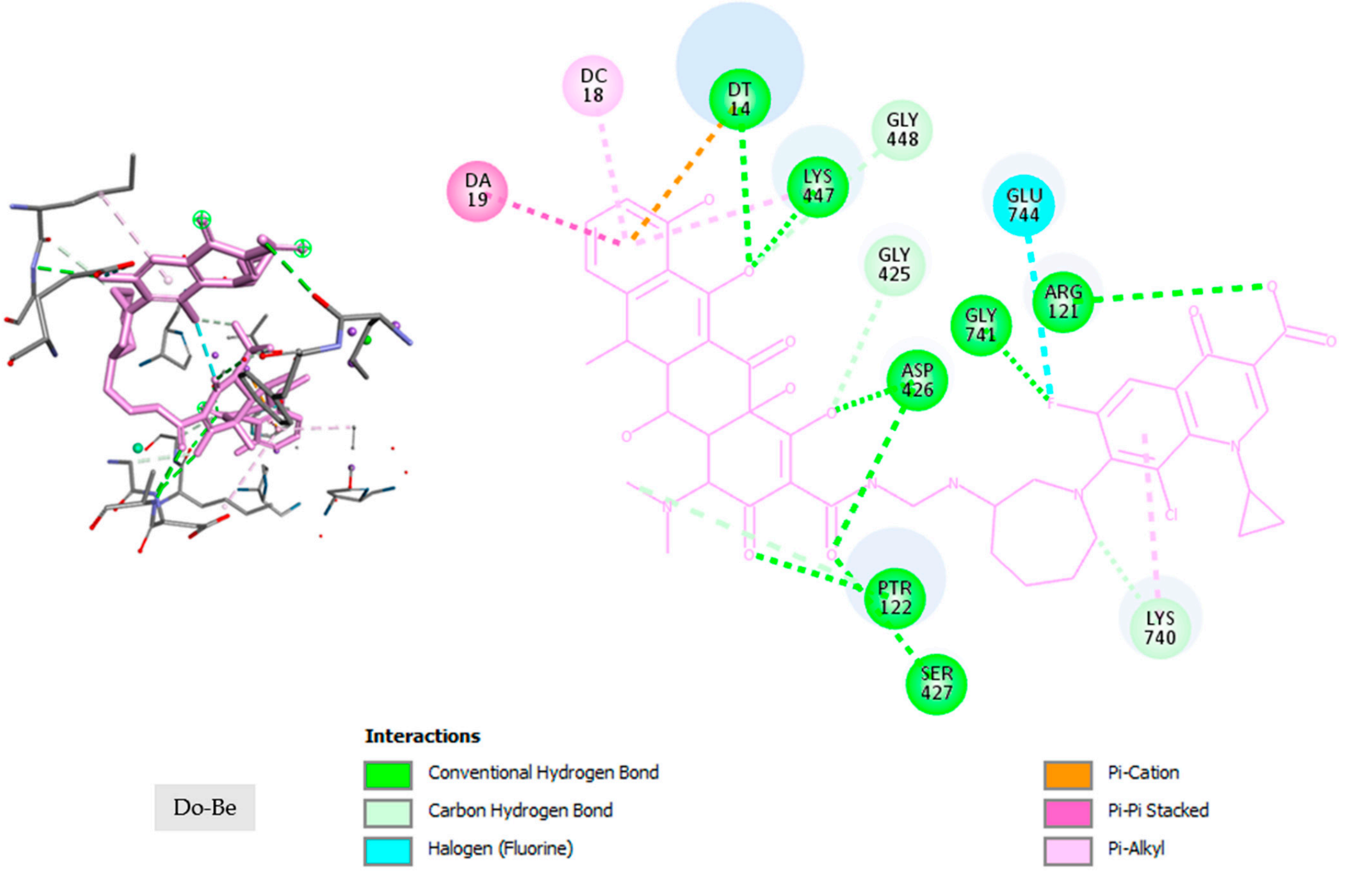

Do-Be

Figure S2. (part 1 of 20). Interactions of the hybrids with the binding pocket of *E. coli* gyrase holo-complex with 217 bp DNA obtained in the self-docking phase for albicidin (in 3D (left) and 2D (right)).

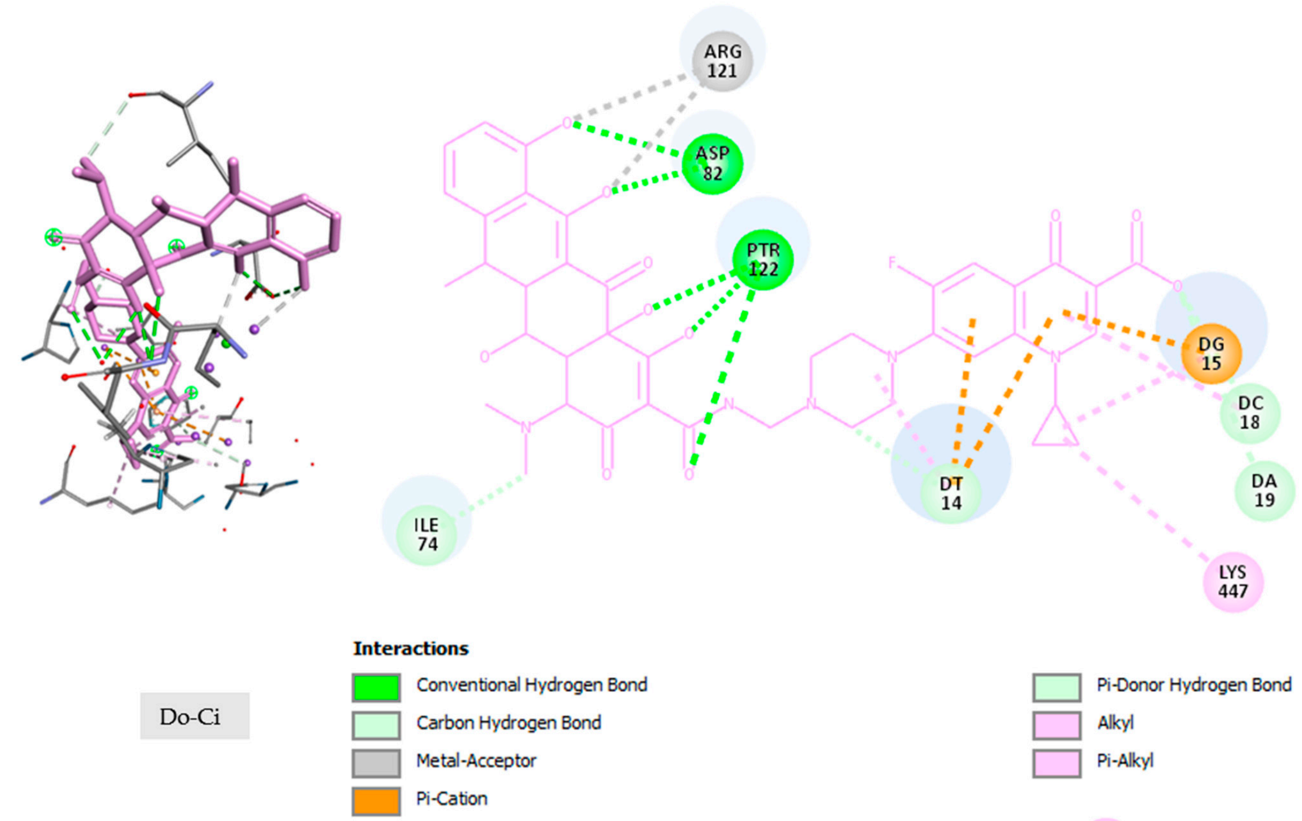

Do-Ci

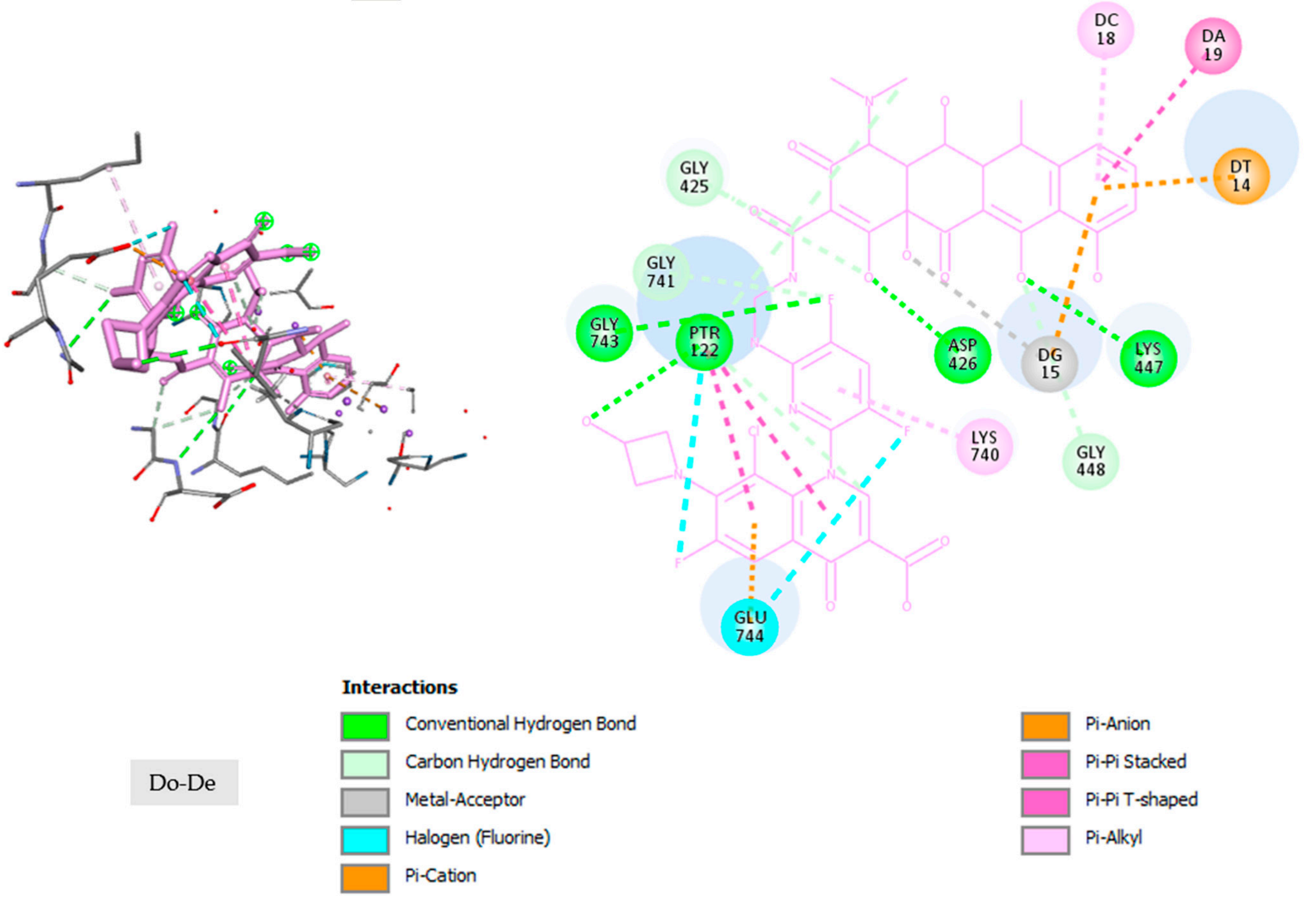

Do-De

Figure S2. (part 2 of 20). Interactions of the hybrids with the binding pocket of *E. coli* gyrase holo-complex with 217 bp DNA obtained in the self-docking phase for albicidin (in 3D (left) and 2D (right)).

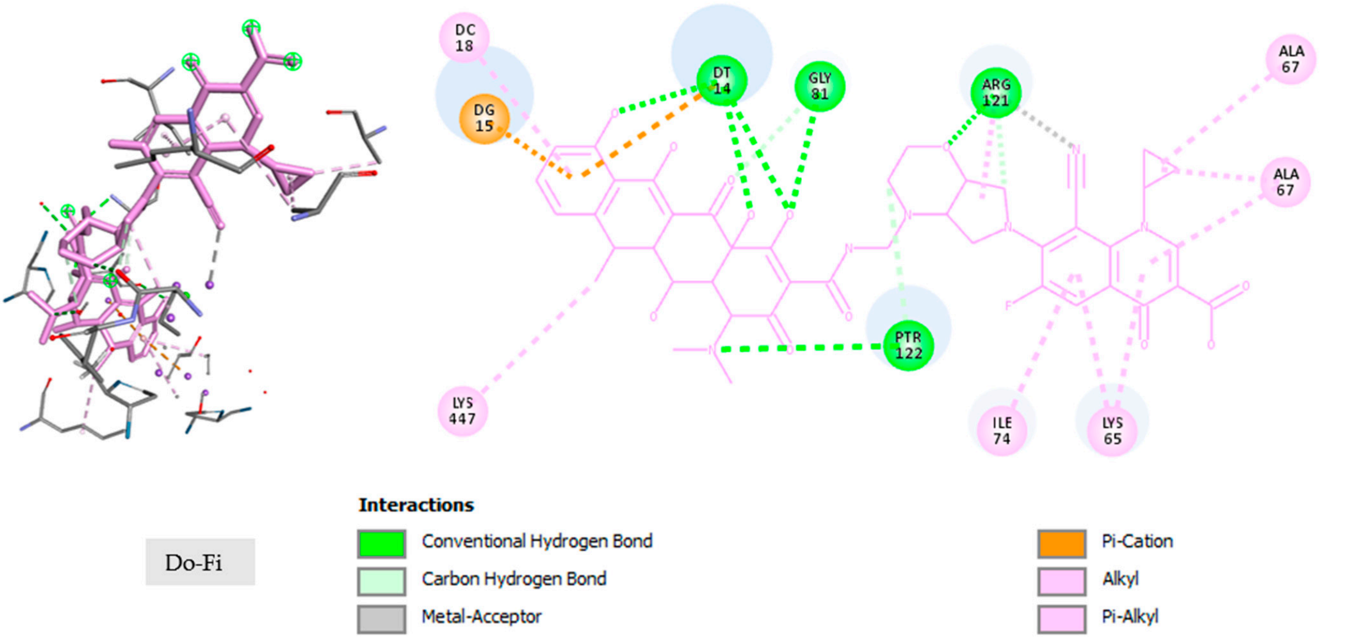

Do-Fi

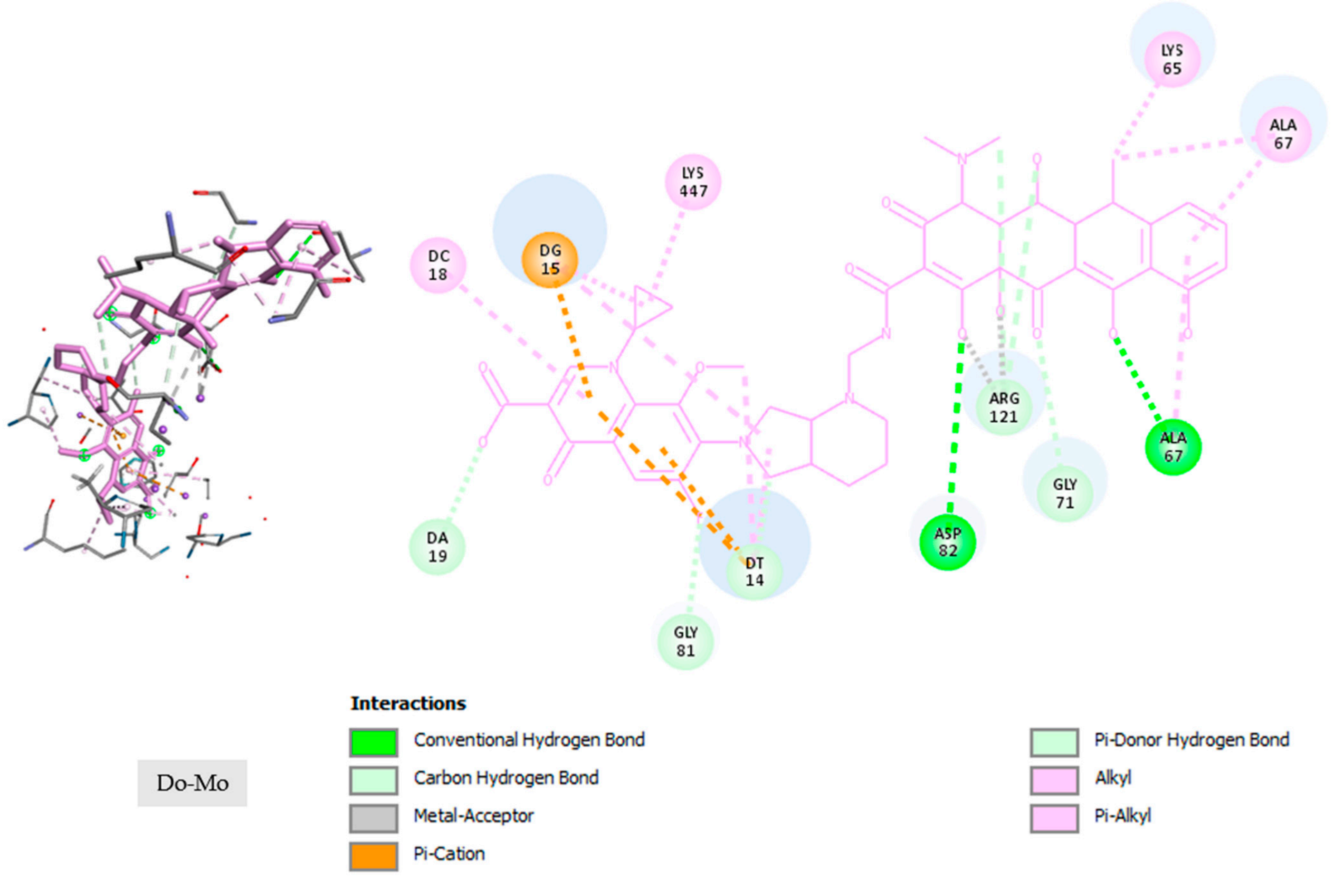

Do-Mo

Figure S2. (part 3 of 20). Interactions of the hybrids with the binding pocket of *E. coli* gyrase holo-complex with 217 bp DNA obtained in the self-docking phase for albicidin (in 3D (left) and 2D (right)).

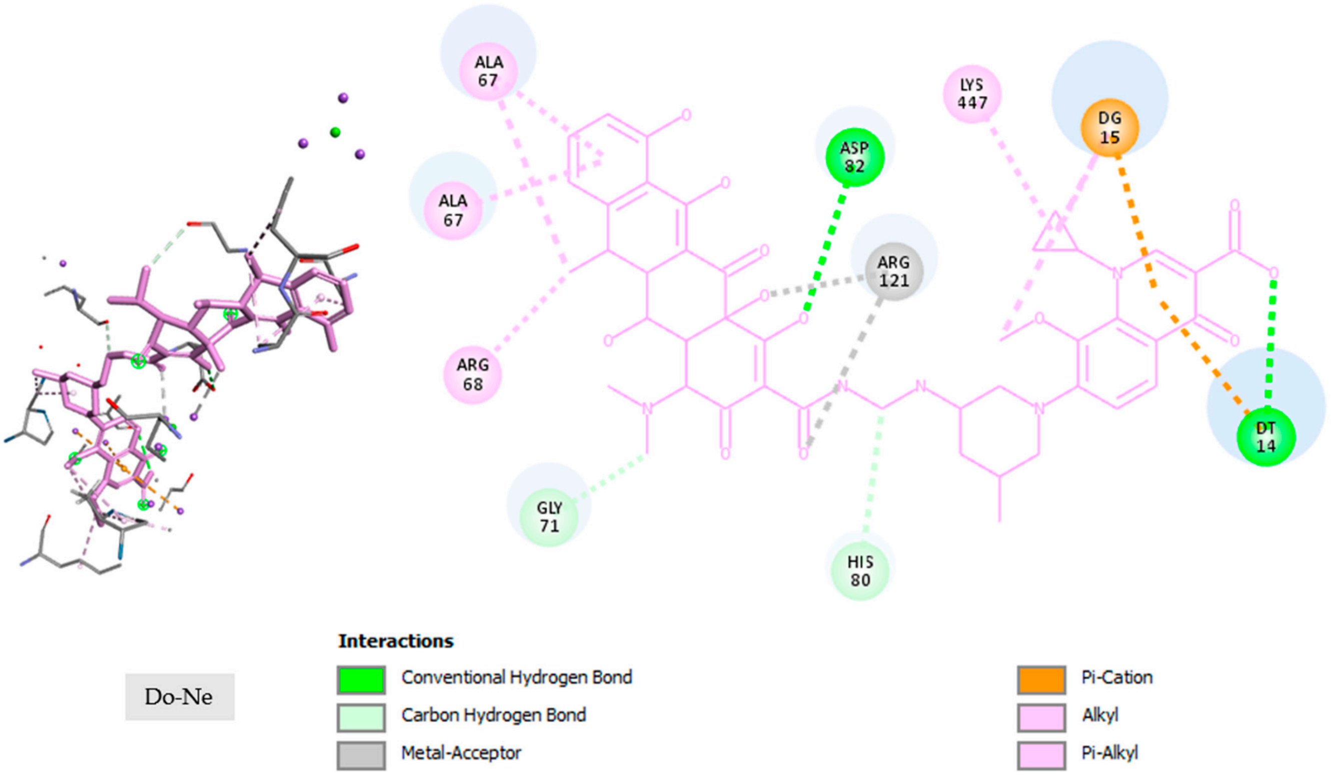

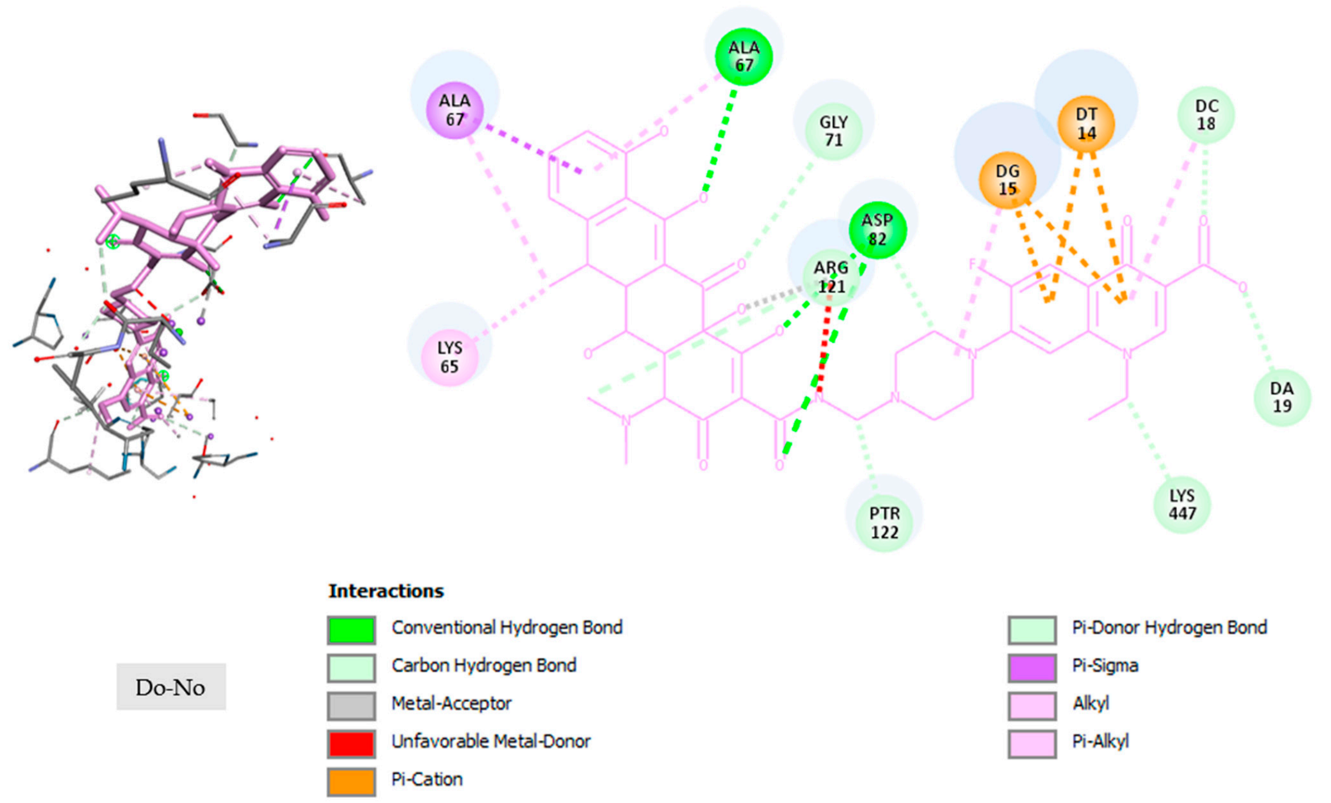

Figure S2. (part 4 of 20). Interactions of the hybrids with the binding pocket of *E. coli* gyrase holo-complex with 217 bp DNA obtained in the self-docking phase for albicidin (in 3D (left) and 2D (right)).

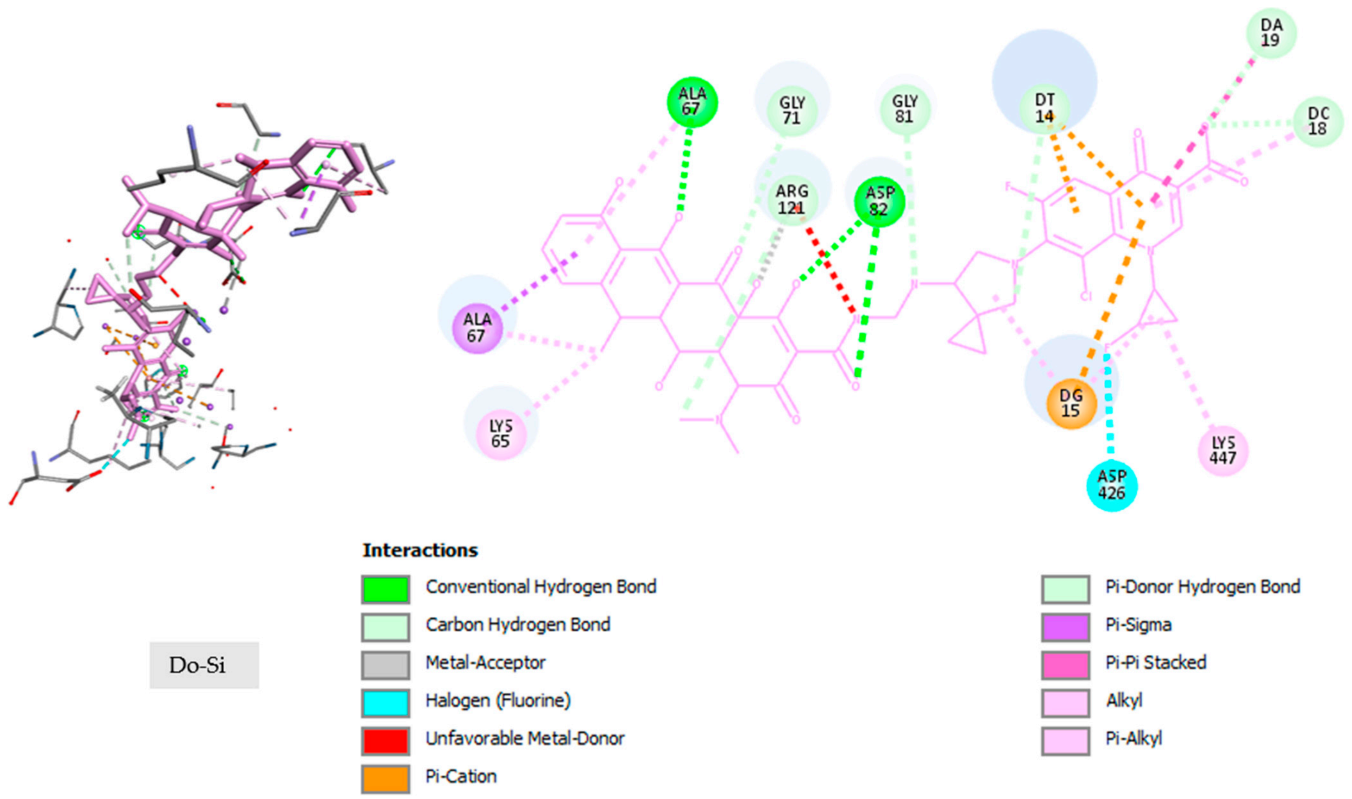

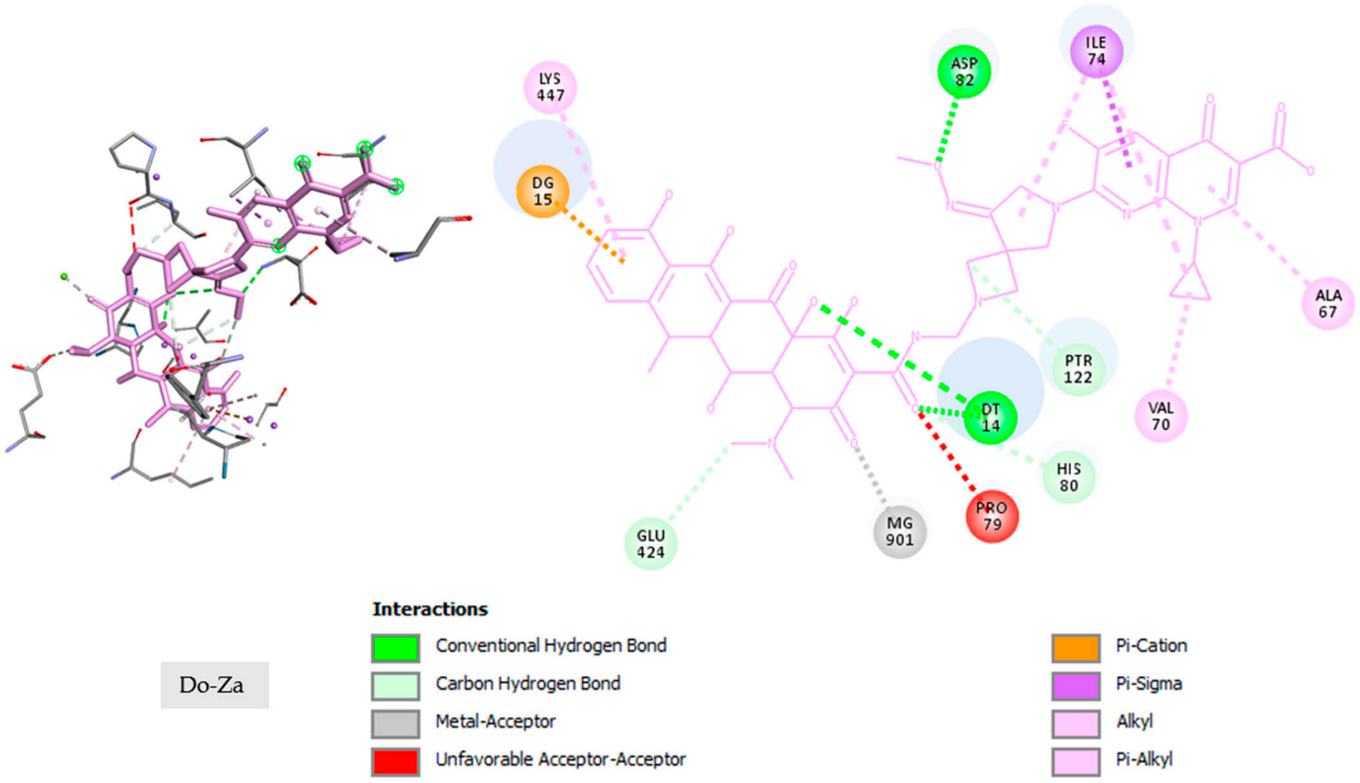

Figure S2. (part 5 of 20). Interactions of the hybrids with the binding pocket of *E. coli* gyrase holo-complex with 217 bp DNA obtained in the self-docking phase for albicidin (in 3D (left) and 2D (right)).

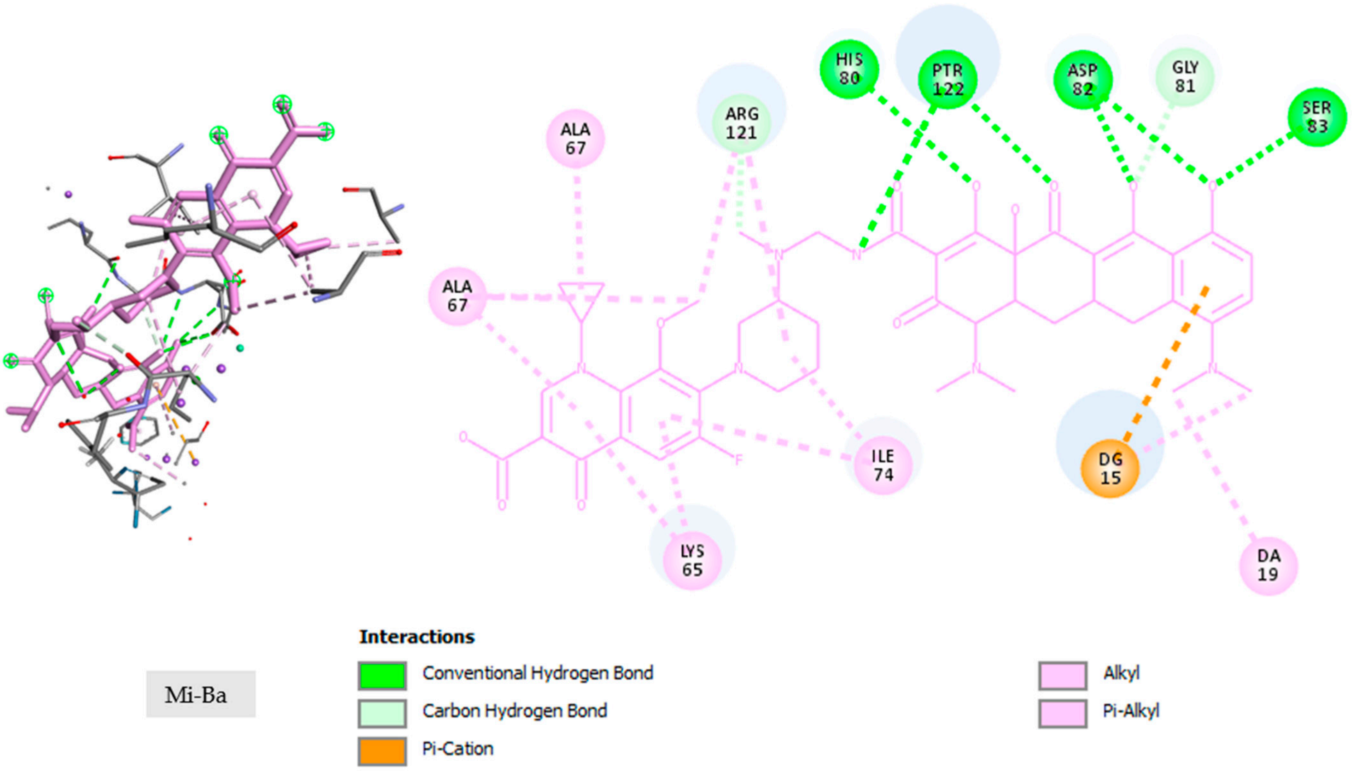

Mi-Ba

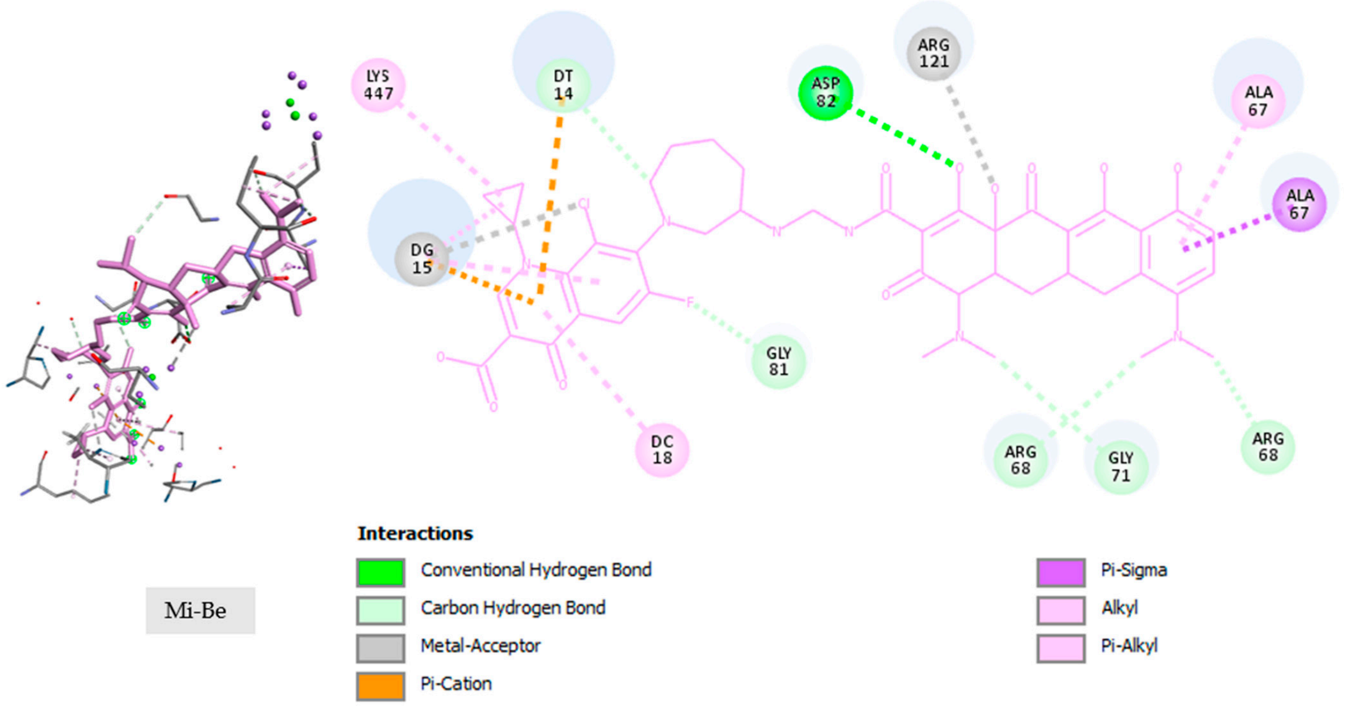

Mi-Be

Figure S2. (part 6 of 20). Interactions of the hybrids with the binding pocket of *E. coli* gyrase holo-complex with 217 bp DNA obtained in the self-docking phase for albicidin (in 3D (left) and 2D (right)).

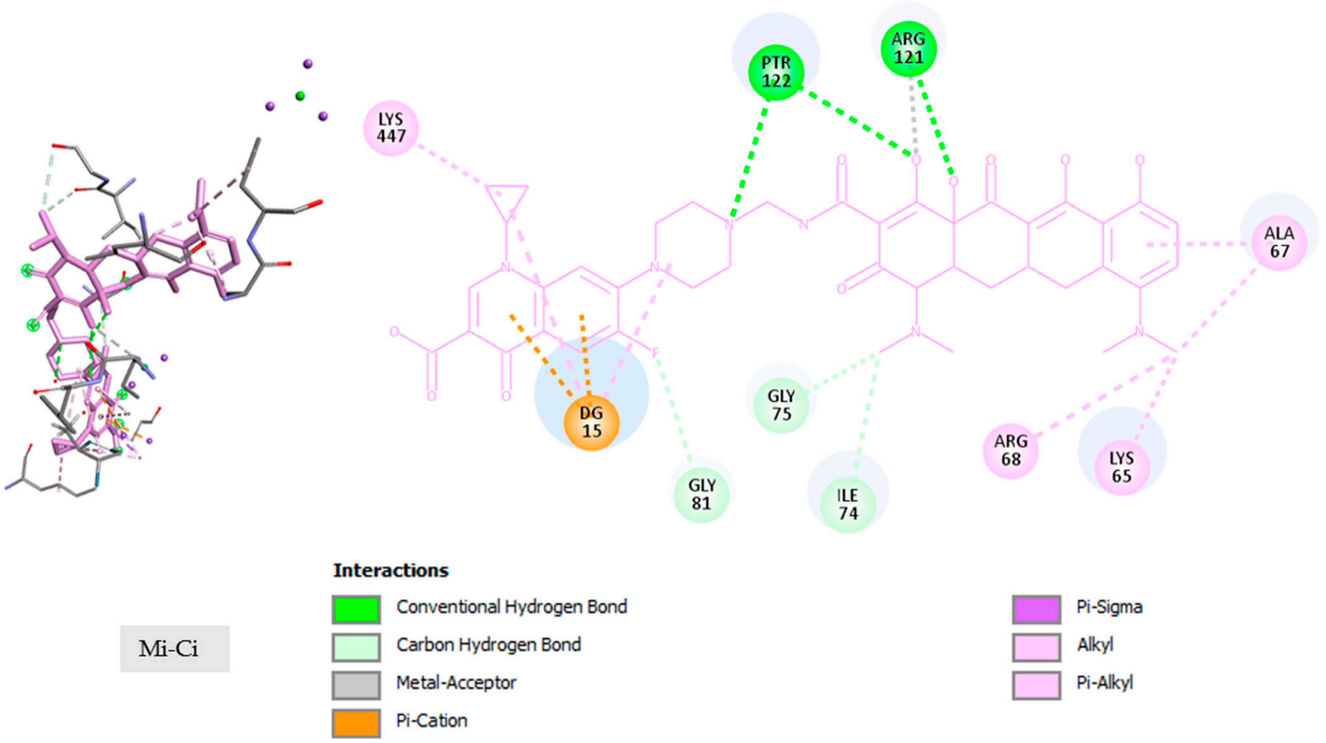

Mi-Ci

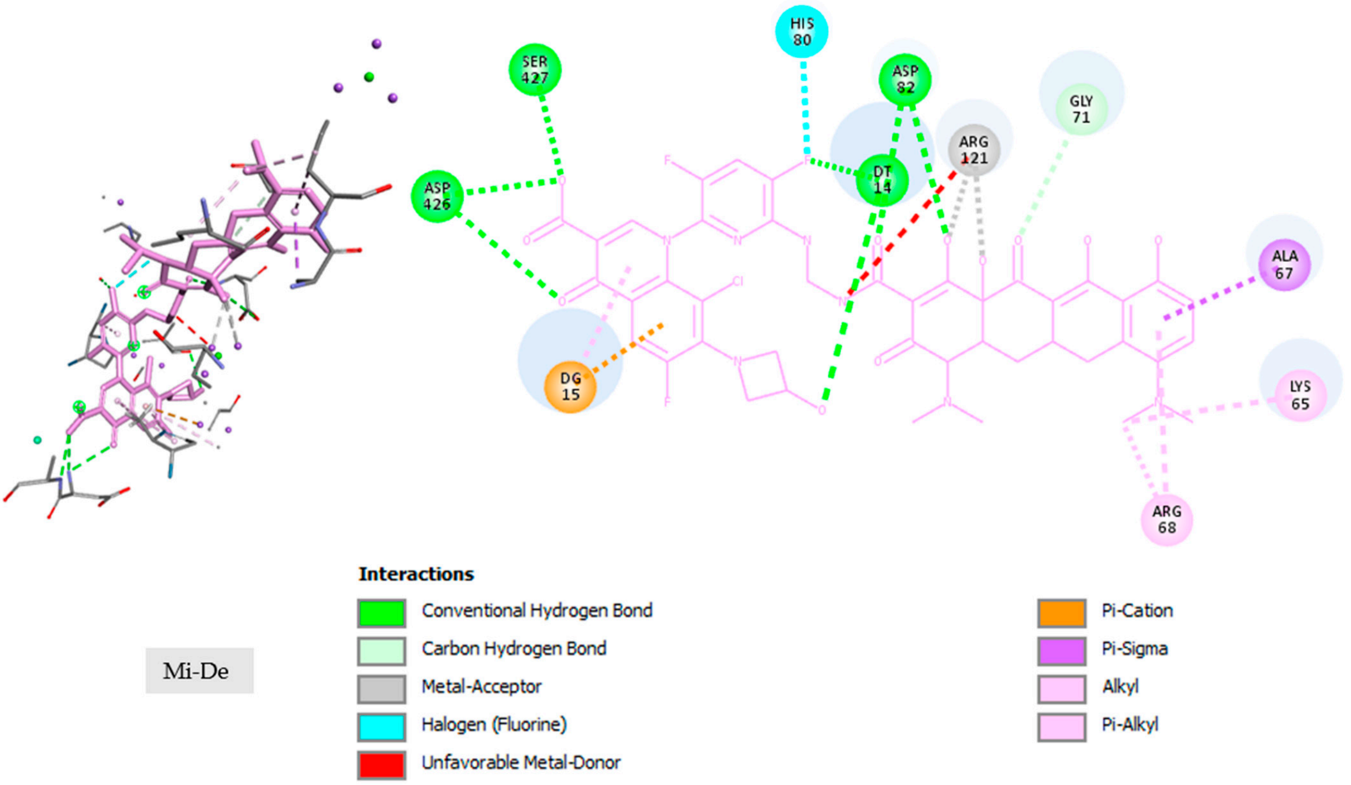

Mi-De

Figure S2. (part 7 of 20). Interactions of the hybrids with the binding pocket of *E. coli* gyrase holo-complex with 217 bp DNA obtained in the self-docking phase for albicidin (in 3D (left) and 2D (right)).

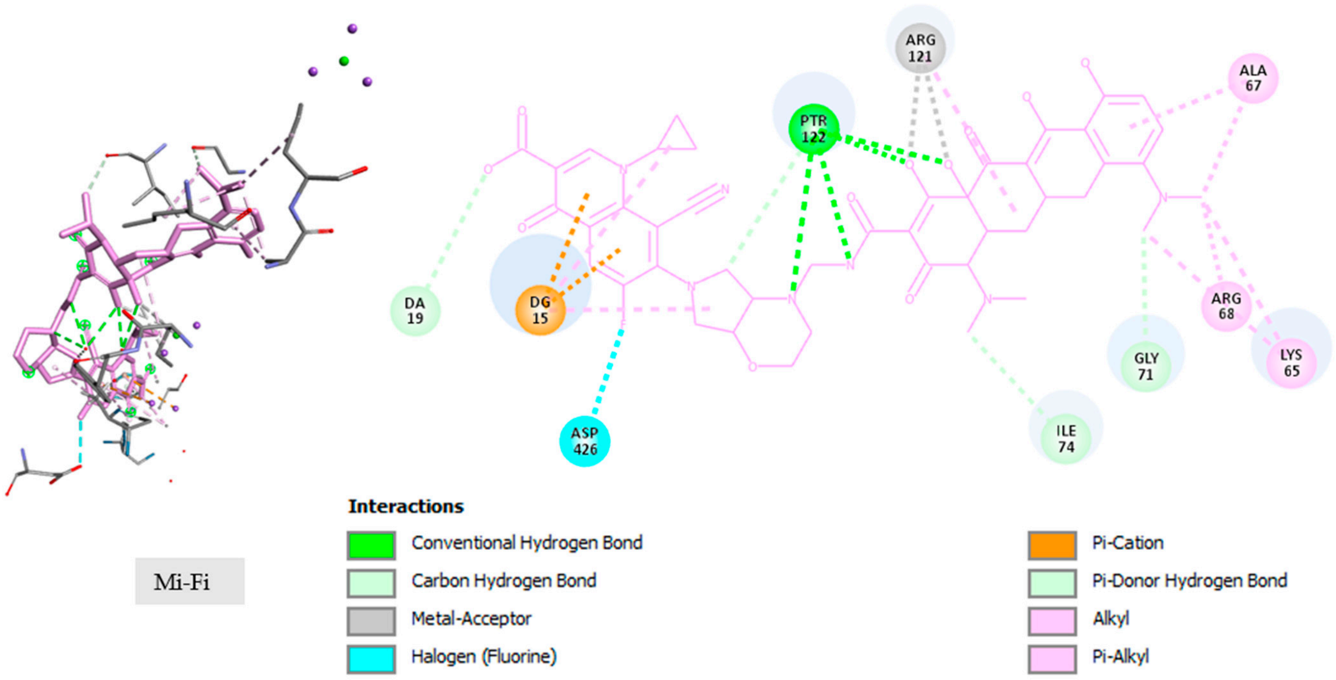

Mi-Fi

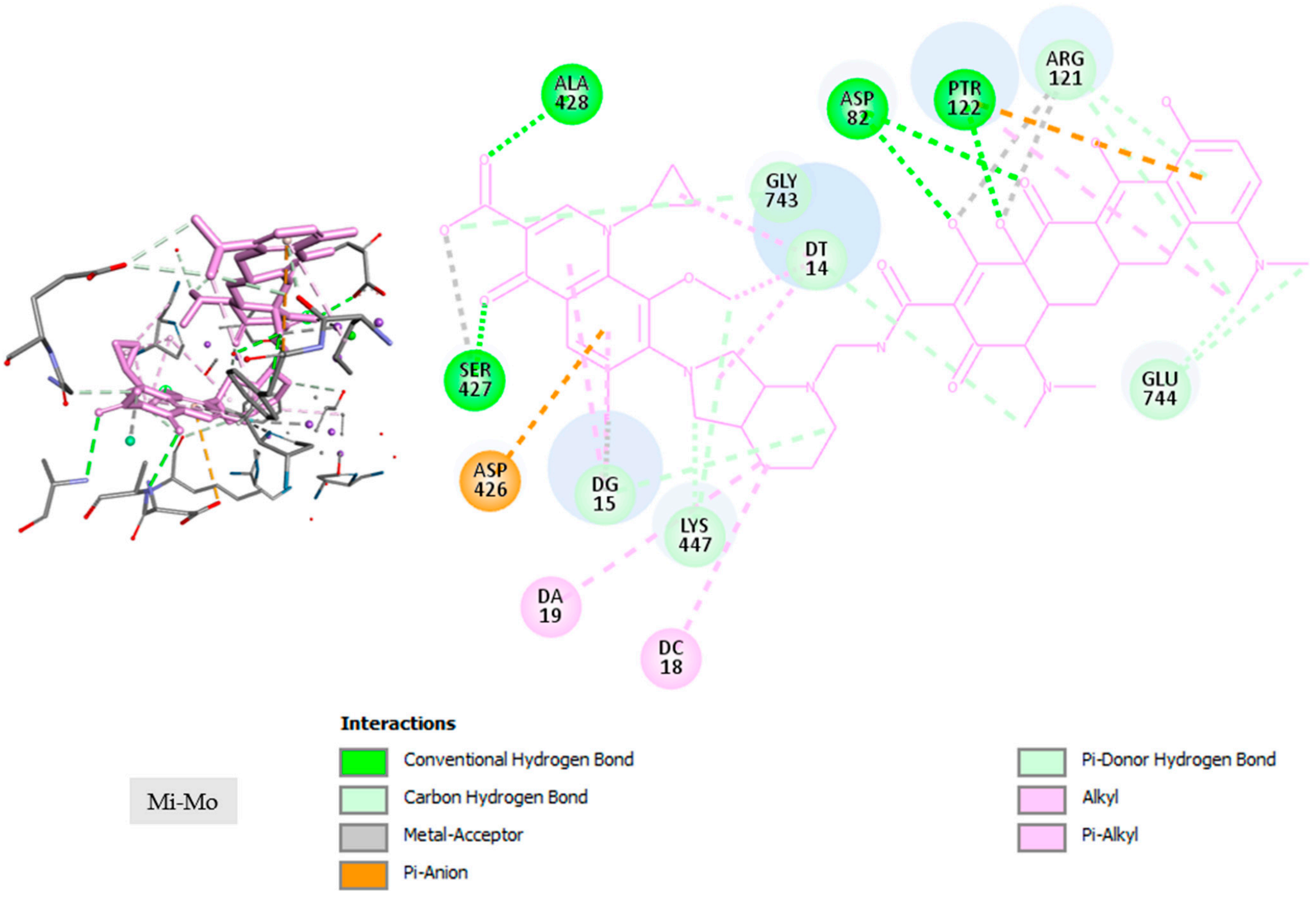

Mi-Mo

Figure S2. (part 8 of 20). Interactions of the hybrids with the binding pocket of *E. coli* gyrase holo-complex with 217 bp DNA obtained in the self-docking phase for albicidin (in 3D (left) and 2D (right)).

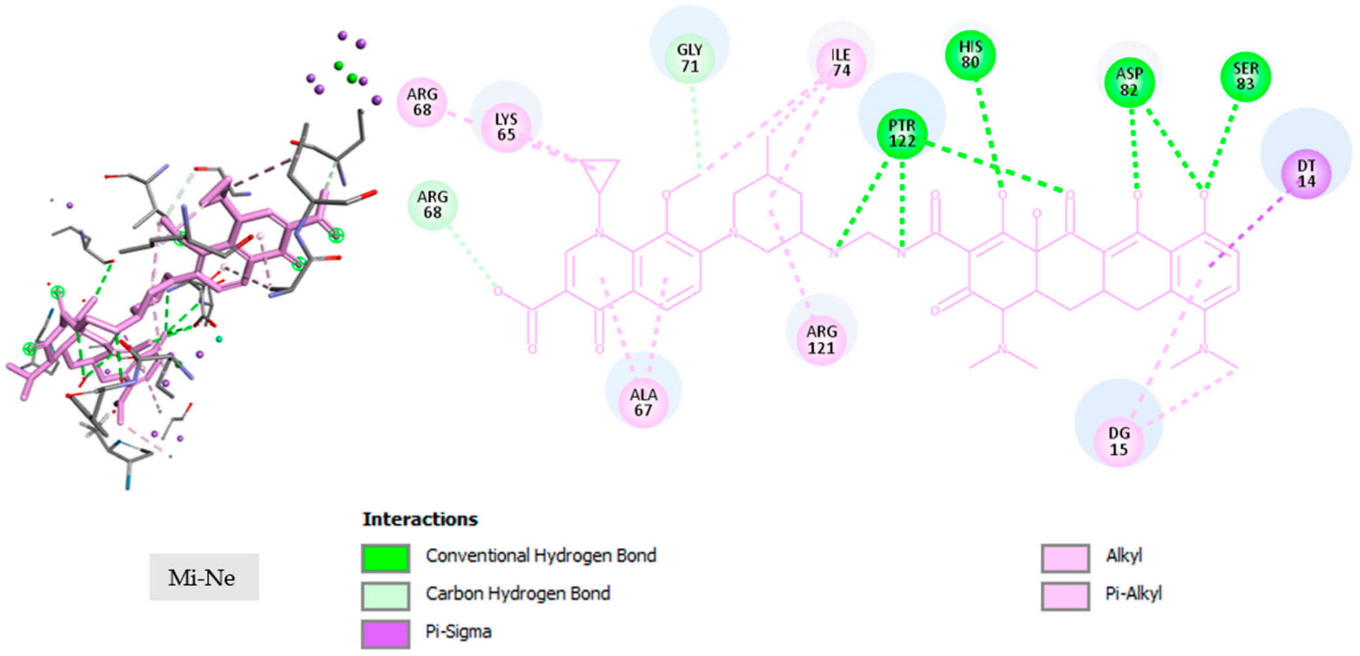

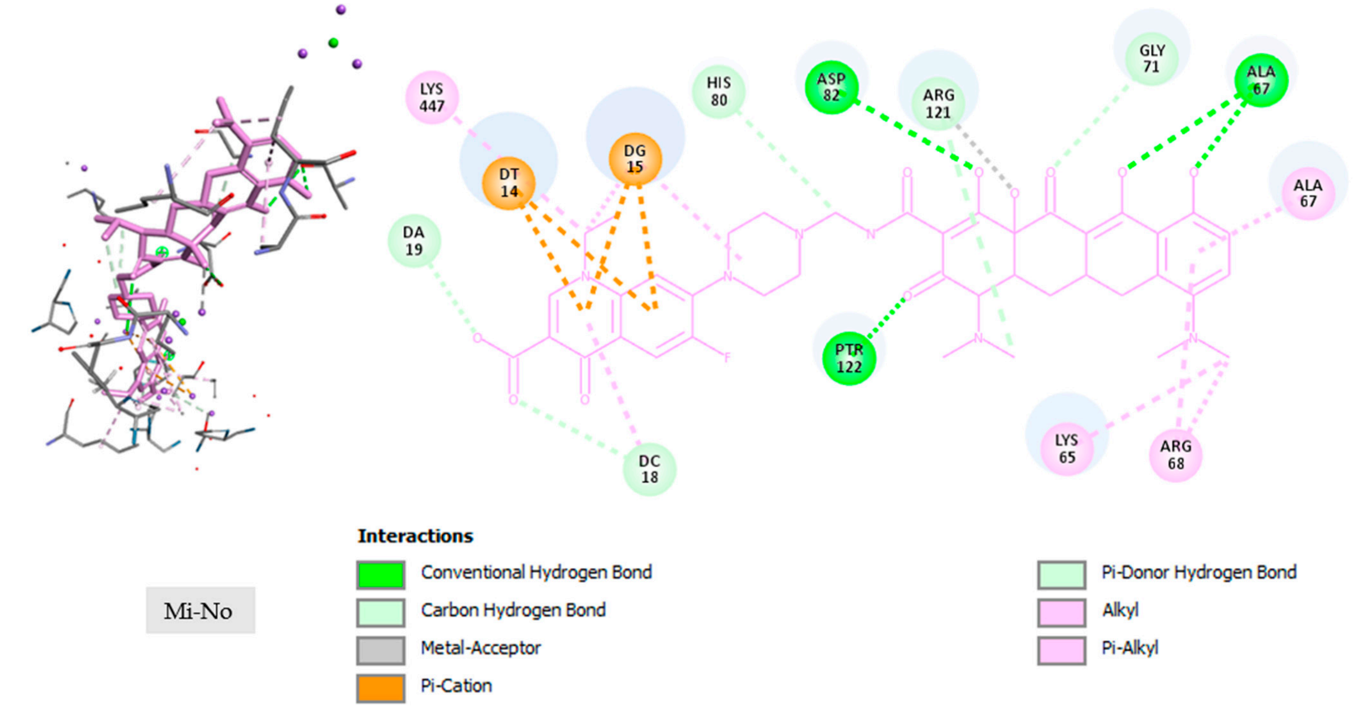

Figure S2. (part 9 of 20). Interactions of the hybrids with the binding pocket of *E. coli* gyrase holo-complex with 217 bp DNA obtained in the self-docking phase for albicidin (in 3D (left) and 2D (right)).

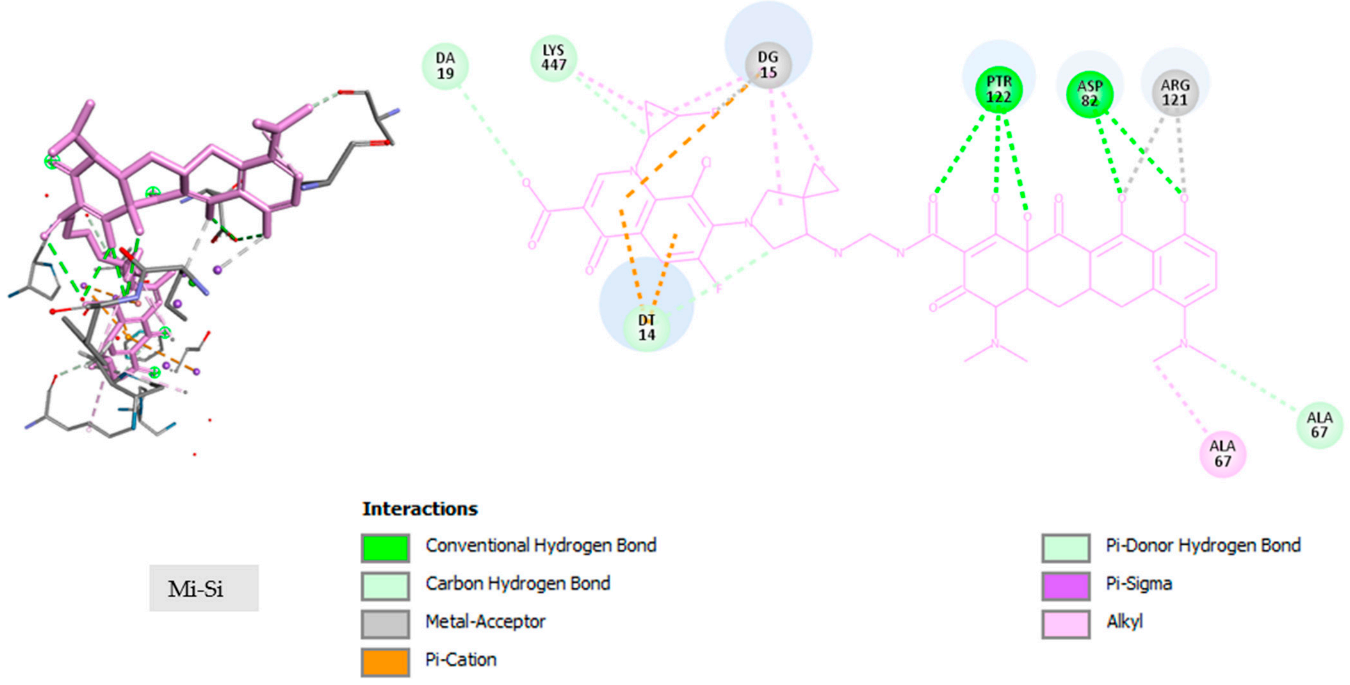

Mi-Si

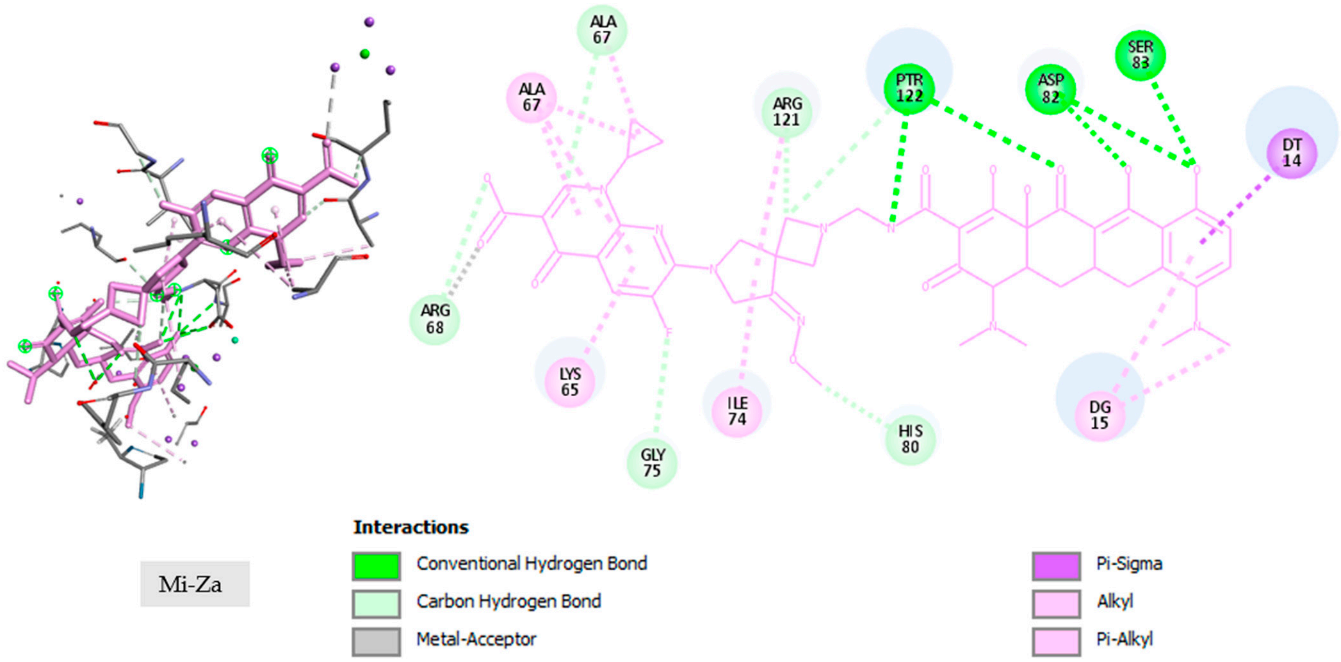

Mi-Za

Figure S2. (part 10 of 20). Interactions of the hybrids with the binding pocket of *E. coli* gyrase holocomplex with 217 bp DNA obtained in the self-docking phase for albicidin (in 3D (left) and 2D (right)).

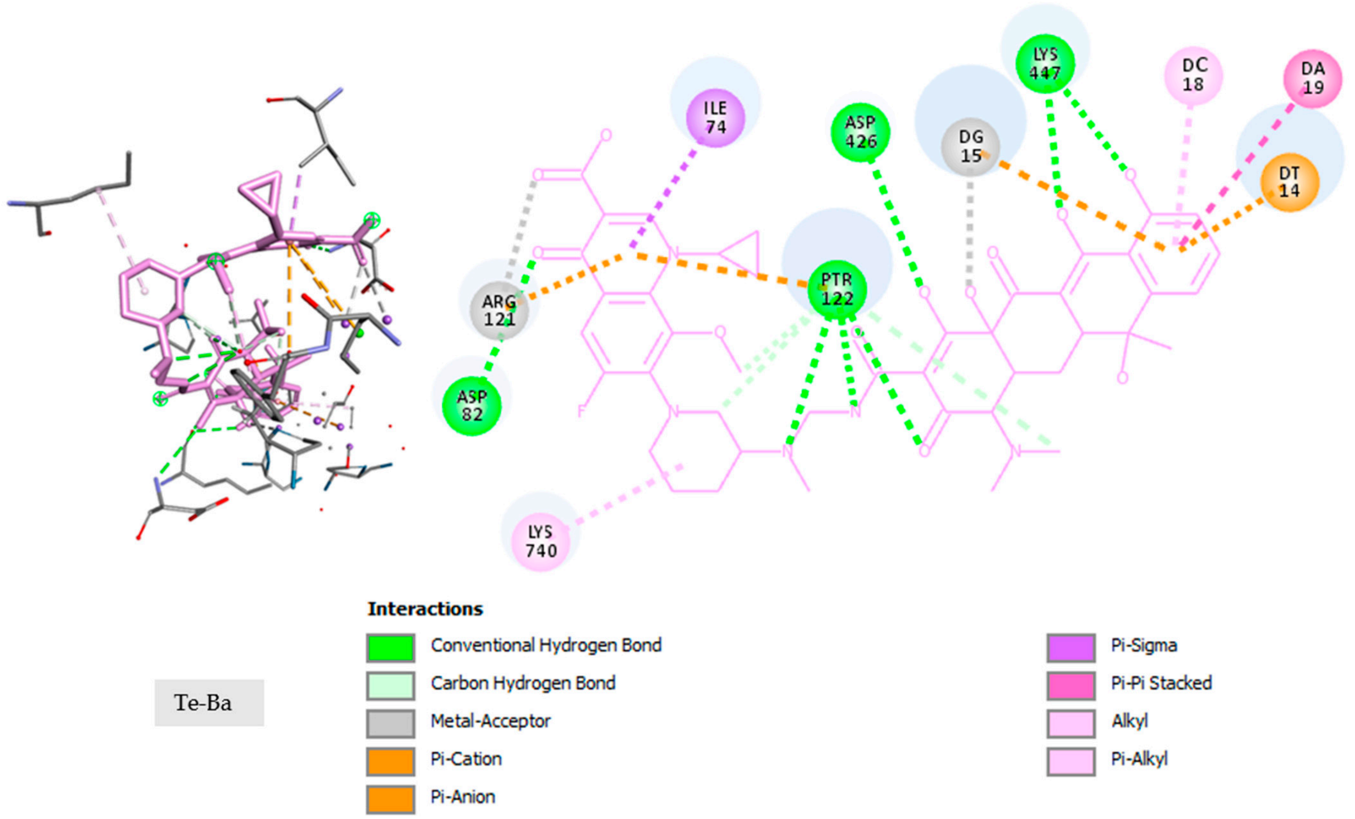

Te-Ba

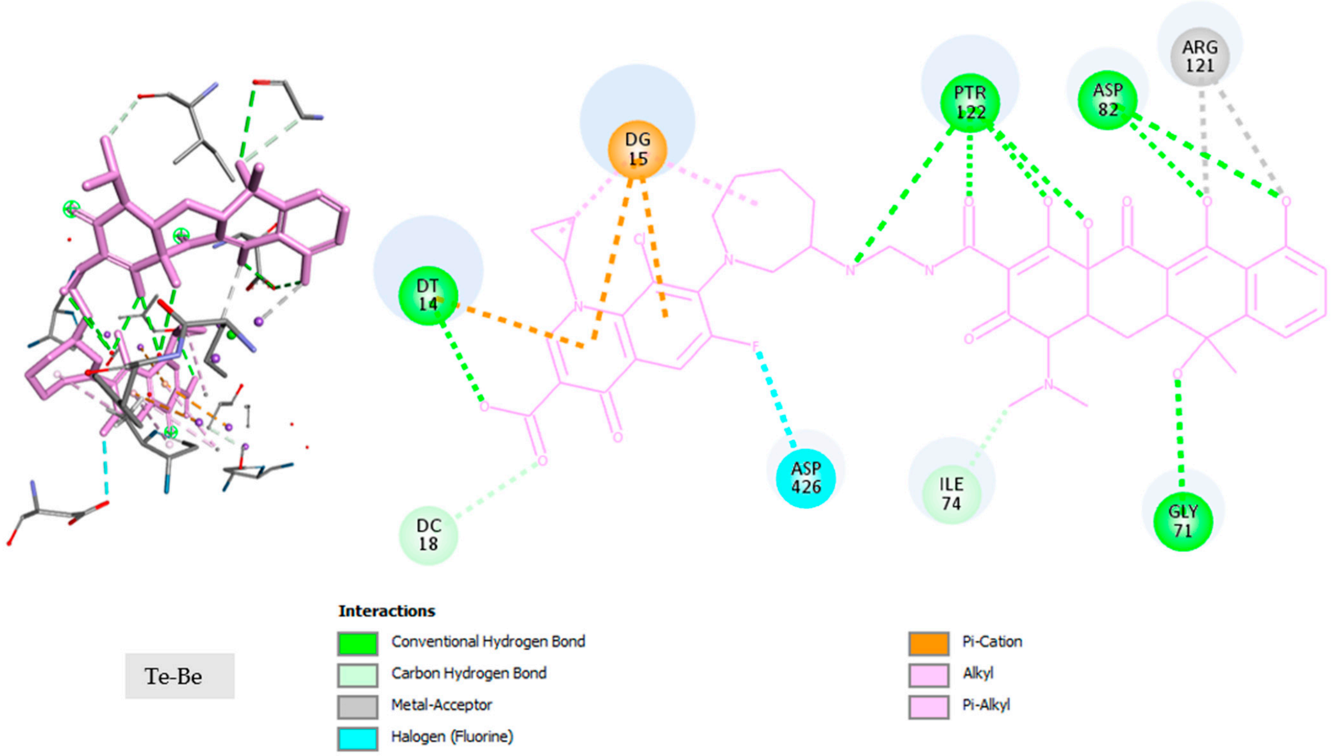

Te-Be

Figure S2. (part 11 of 20). Interactions of the hybrids with the binding pocket of *E. coli* gyrase holocomplex with 217 bp DNA obtained in the self-docking phase for albicidin (in 3D (left) and 2D (right)).

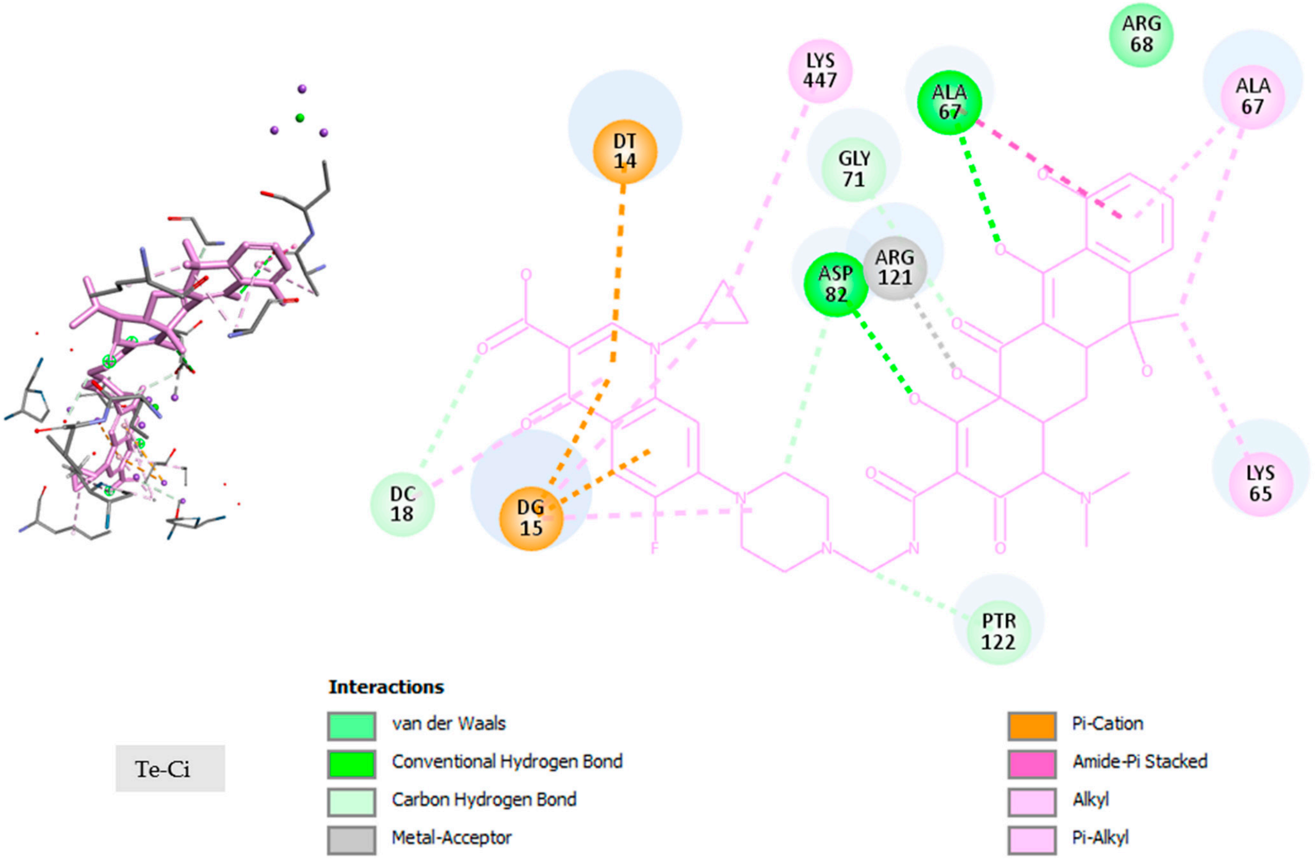

Te-Ci

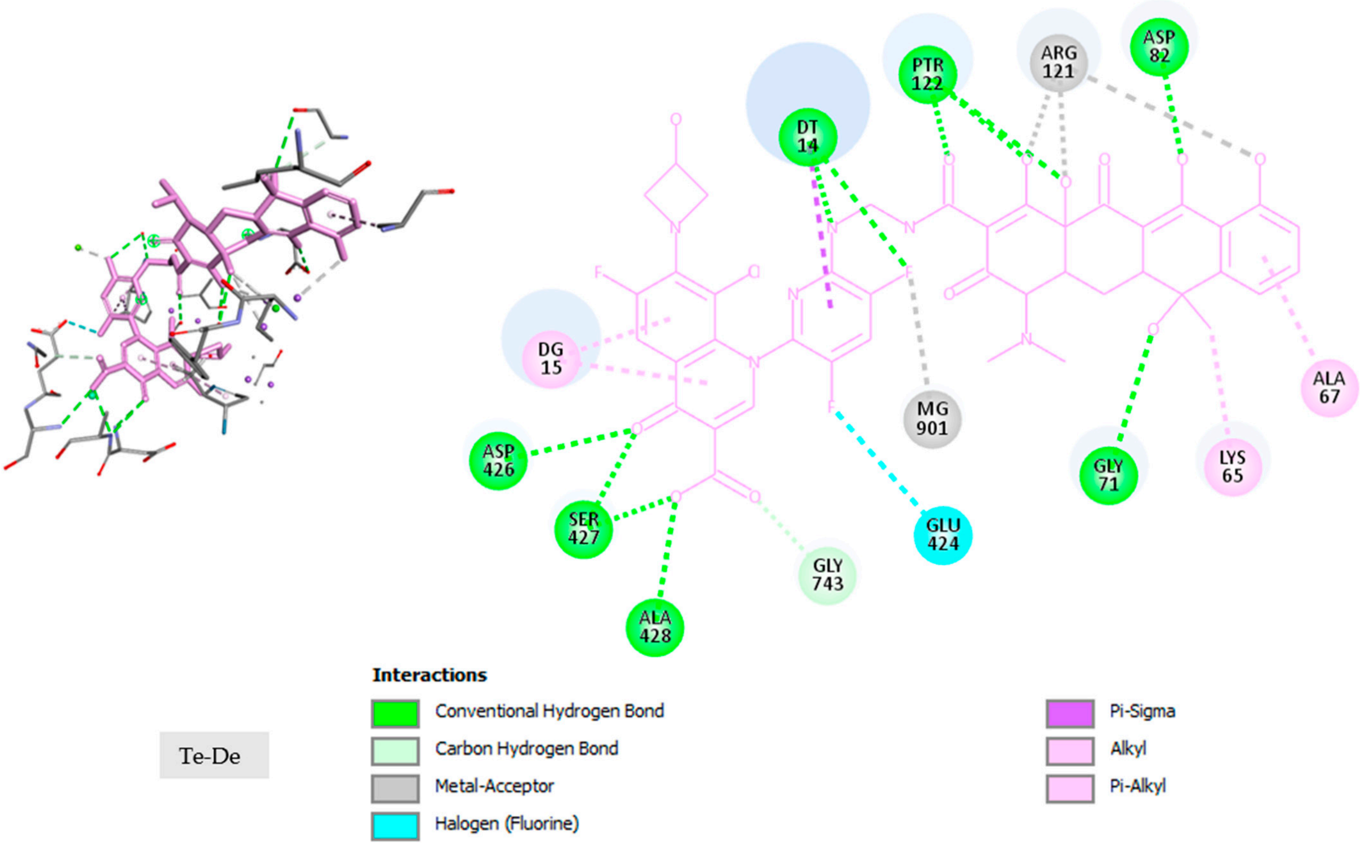

Te-De

Figure S2. (part 12 of 20). Interactions of the hybrids with the binding pocket of *E. coli* gyrase holo-complex with 217 bp DNA obtained in the self-docking phase for albicidin (in 3D (left) and 2D (right)).

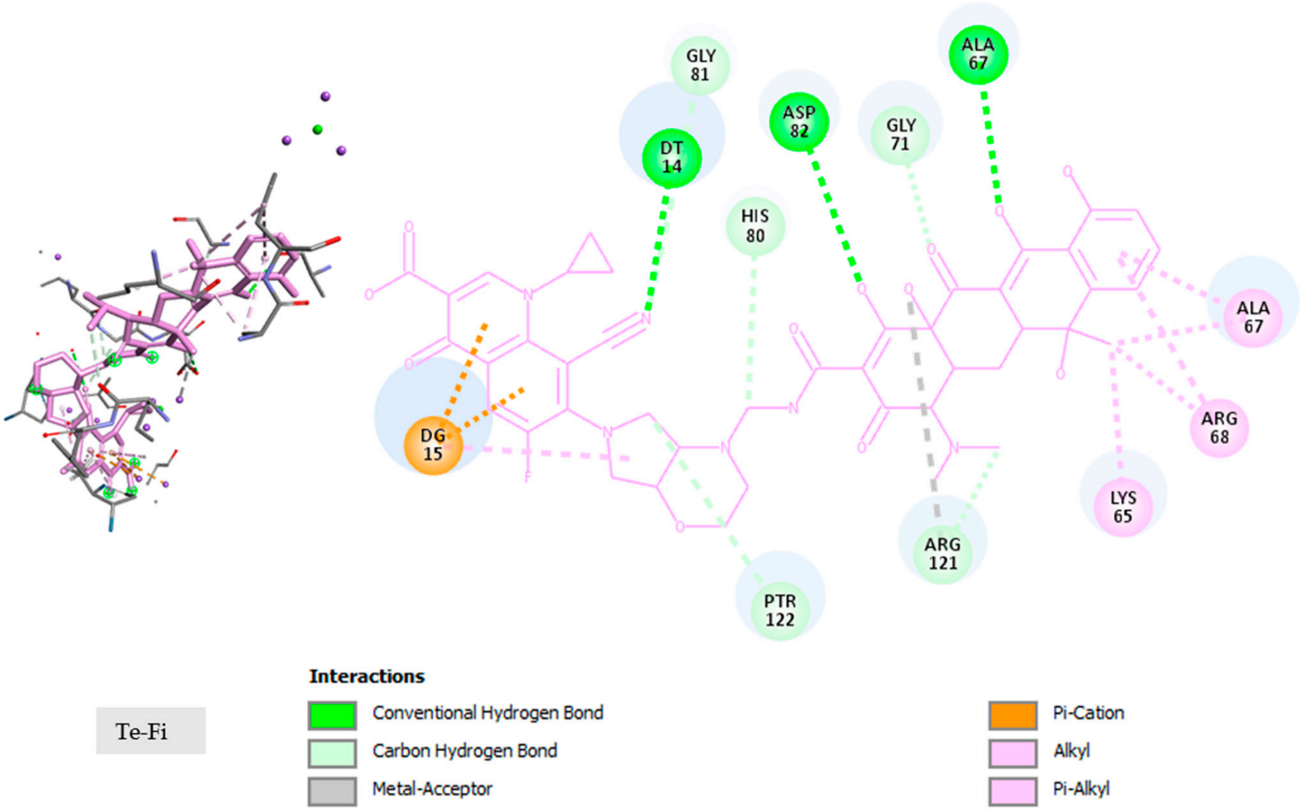

Te-Fi

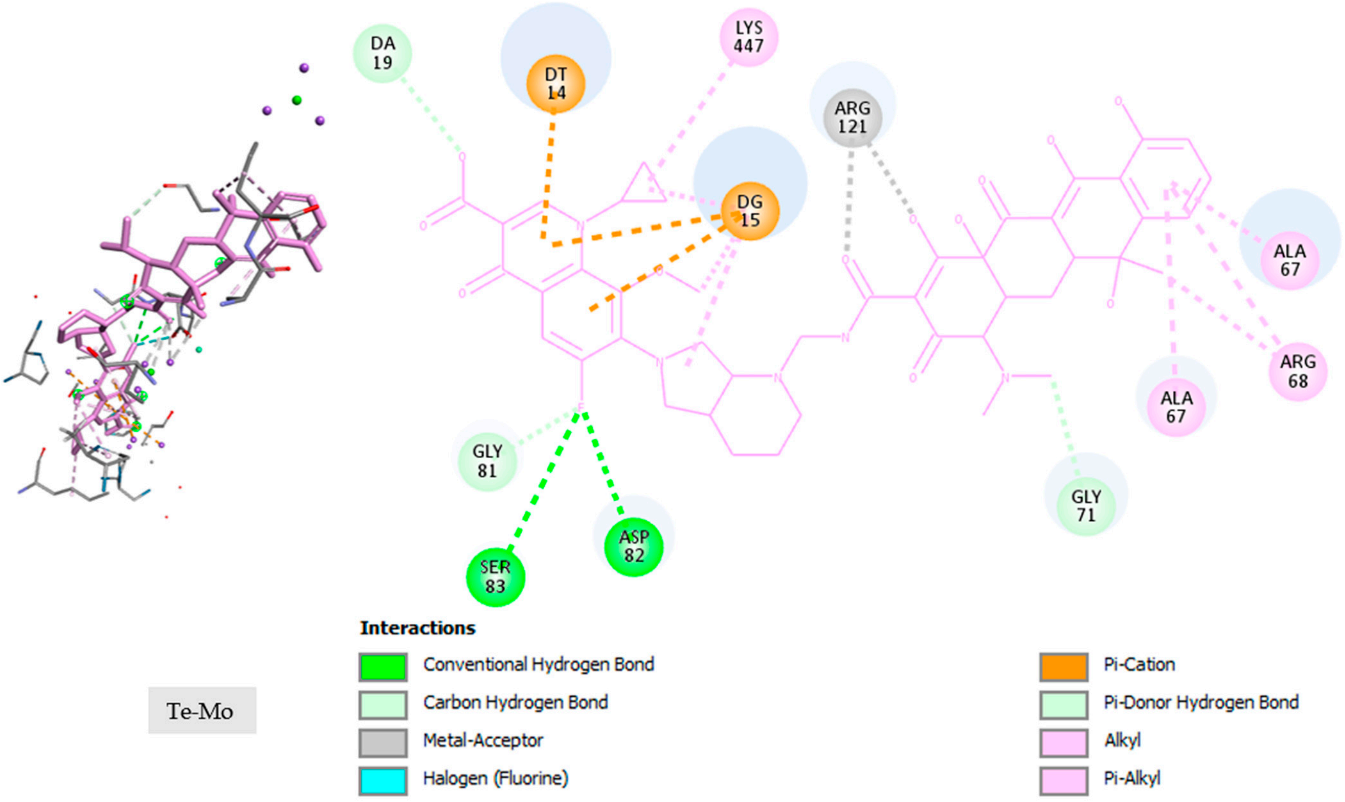

Te-Mo

Figure S2. (part 13 of 20). Interactions of the hybrids with the binding pocket of *E. coli* gyrase holo-complex with 217 bp DNA obtained in the self-docking phase for albicidin (in 3D (left) and 2D (right)).

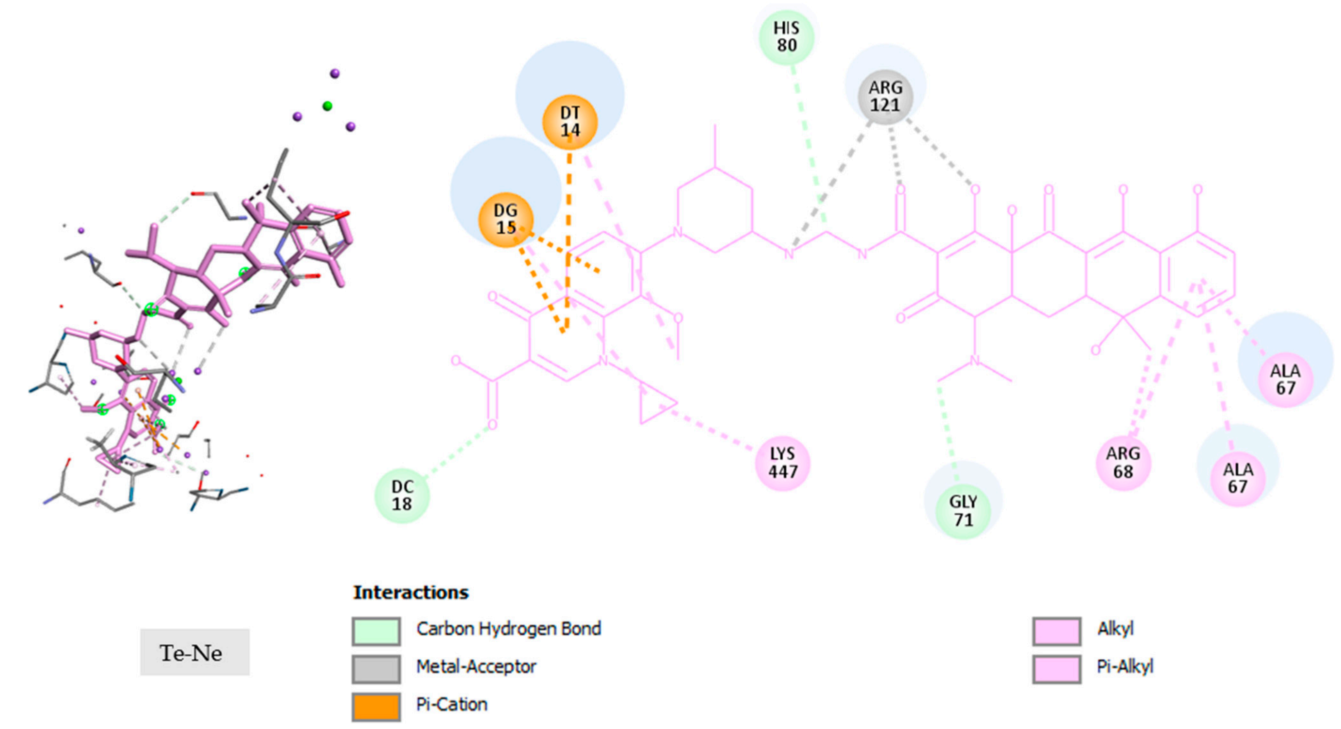

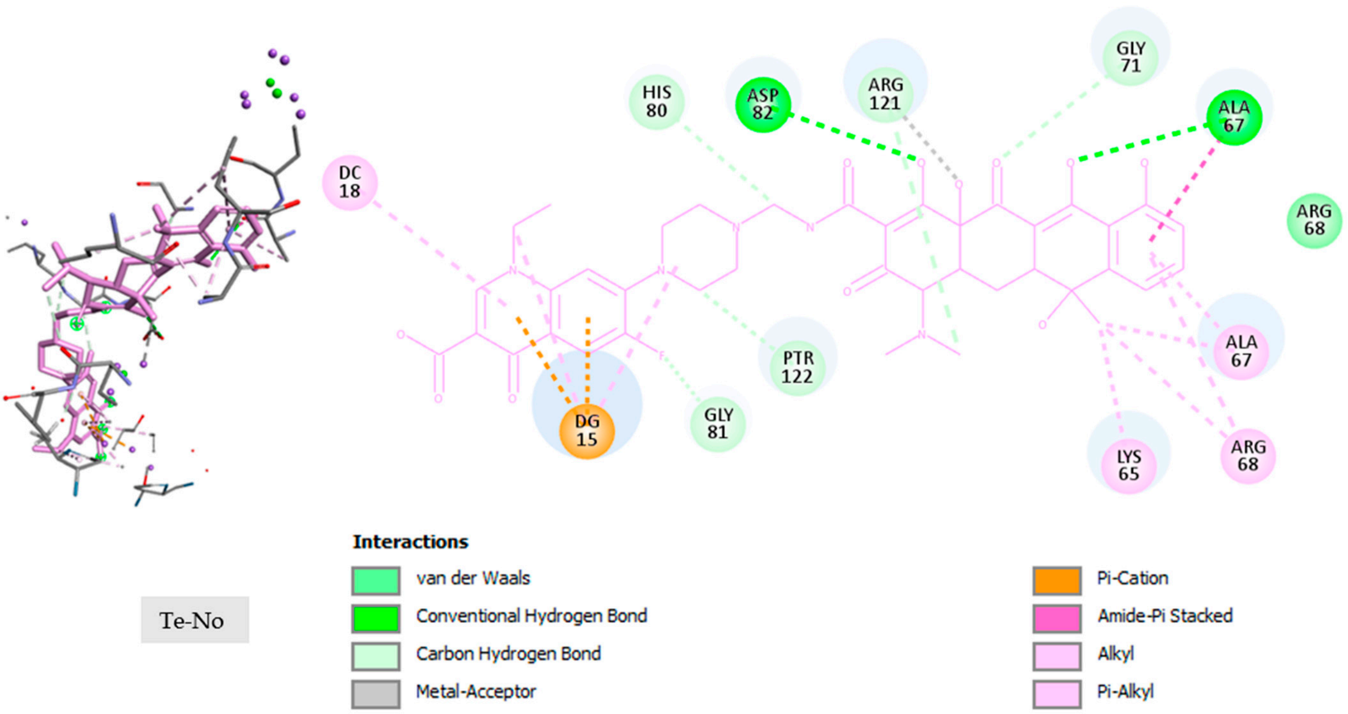

Figure S2. (part 14 of 20). Interactions of the hybrids with the binding pocket of *E. coli* gyrase holo-complex with 217 bp DNA obtained in the self-docking phase for albicidin (in 3D (left) and 2D (right)).

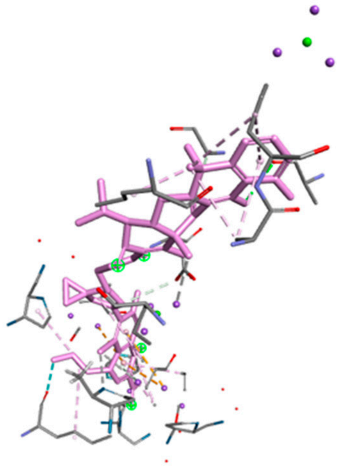

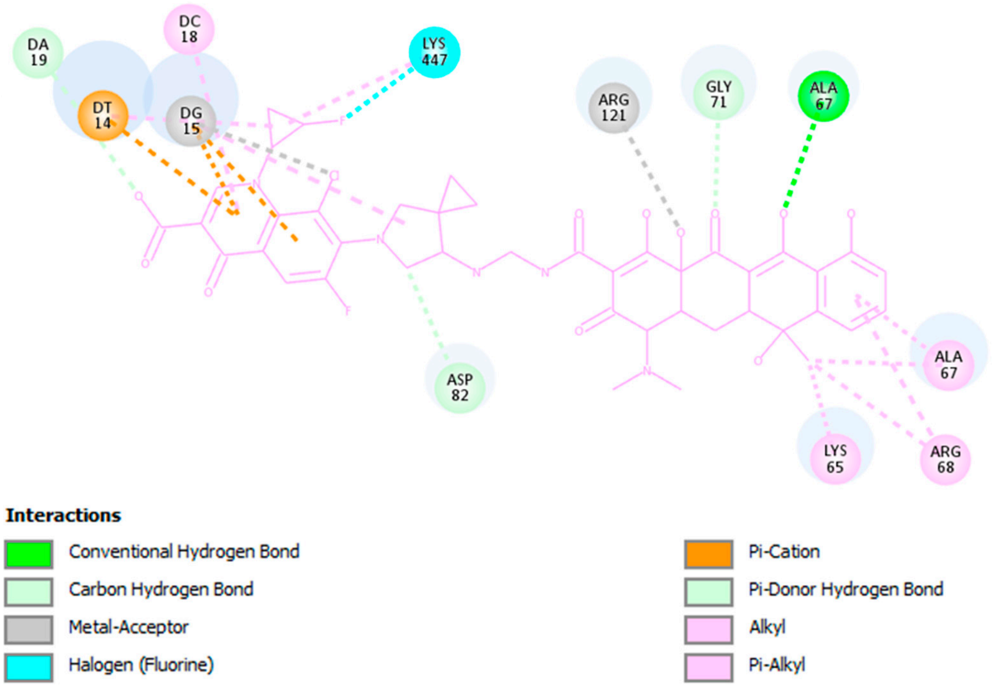

Te-Si

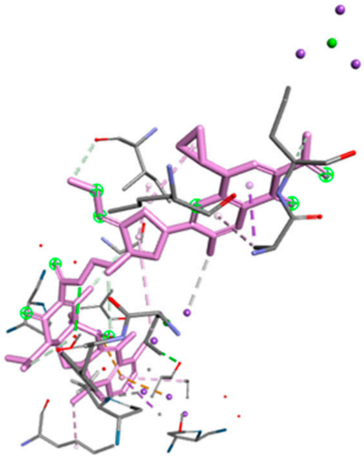

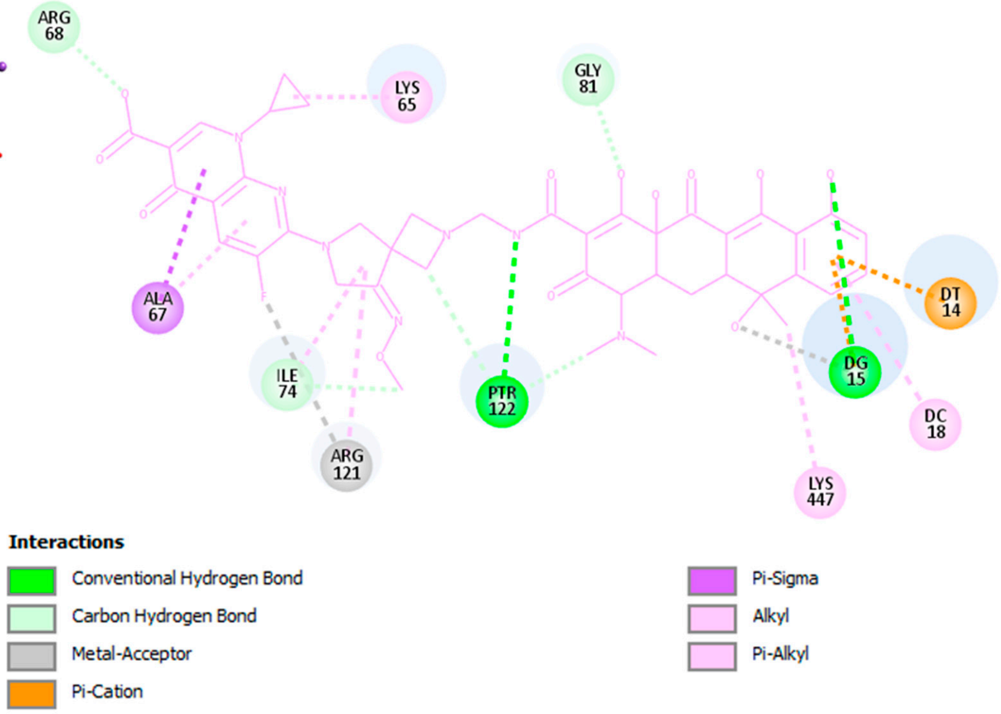

Te-Za

Figure S2. (part 15 of 20). Interactions of the hybrids with the binding pocket of *E. coli* gyrase holo-complex with 217 bp DNA obtained in the self-docking phase for albicidin (in 3D (left) and 2D (right)).

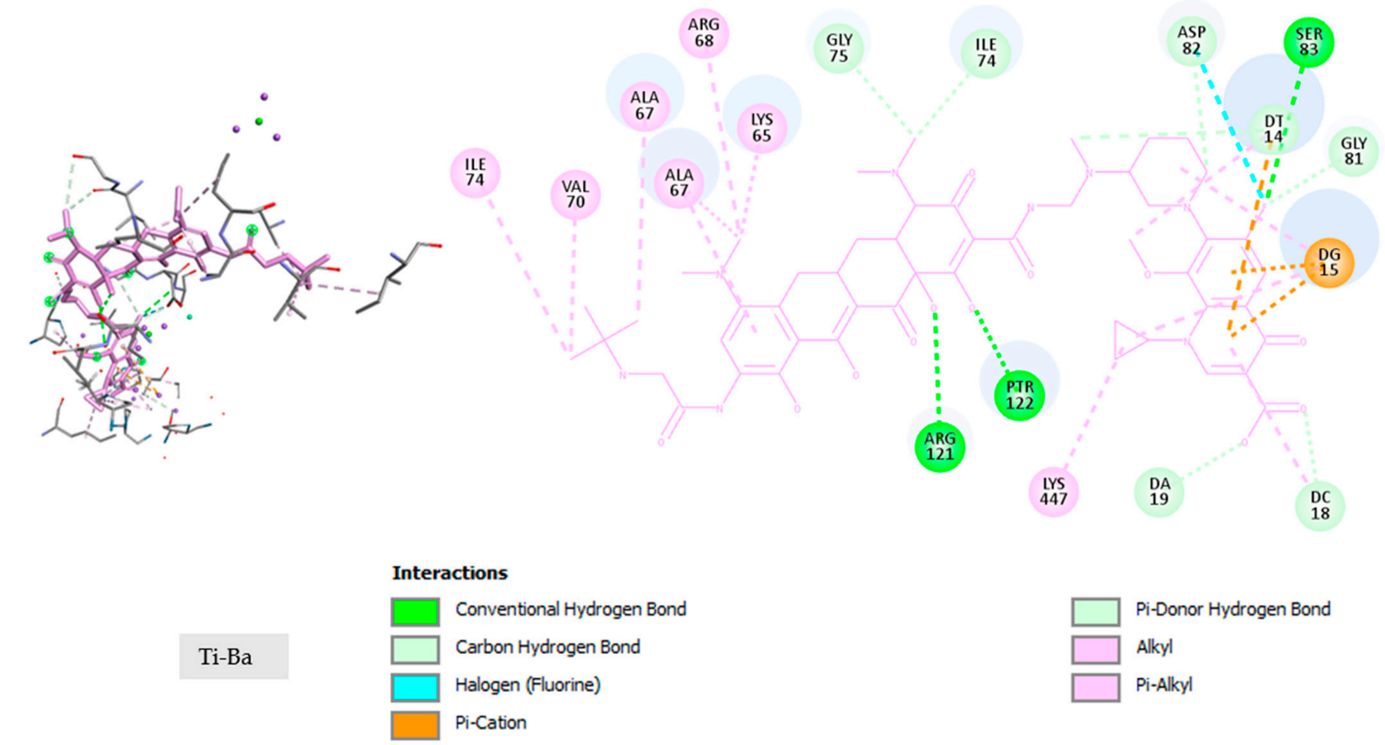

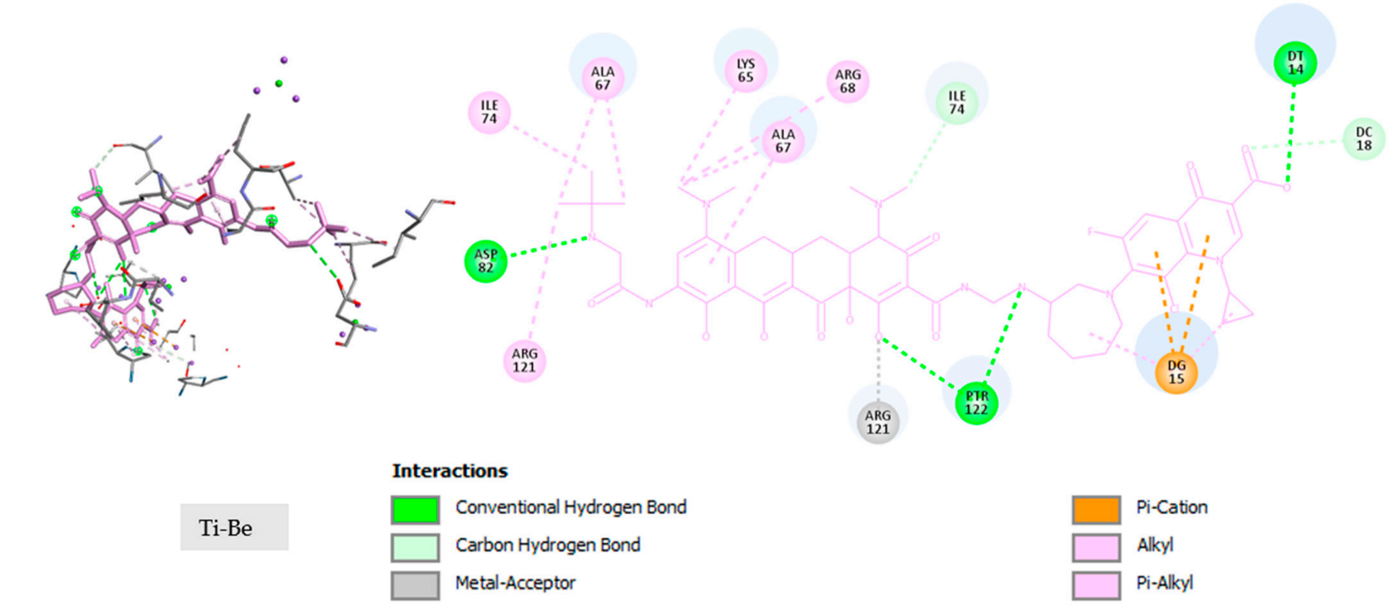

Figure S2. (part 16 of 20). Interactions of the hybrids with the binding pocket of *E. coli* gyrase holocomplex with 217 bp DNA obtained in the self-docking phase for albicidin (in 3D (left) and 2D (right)).

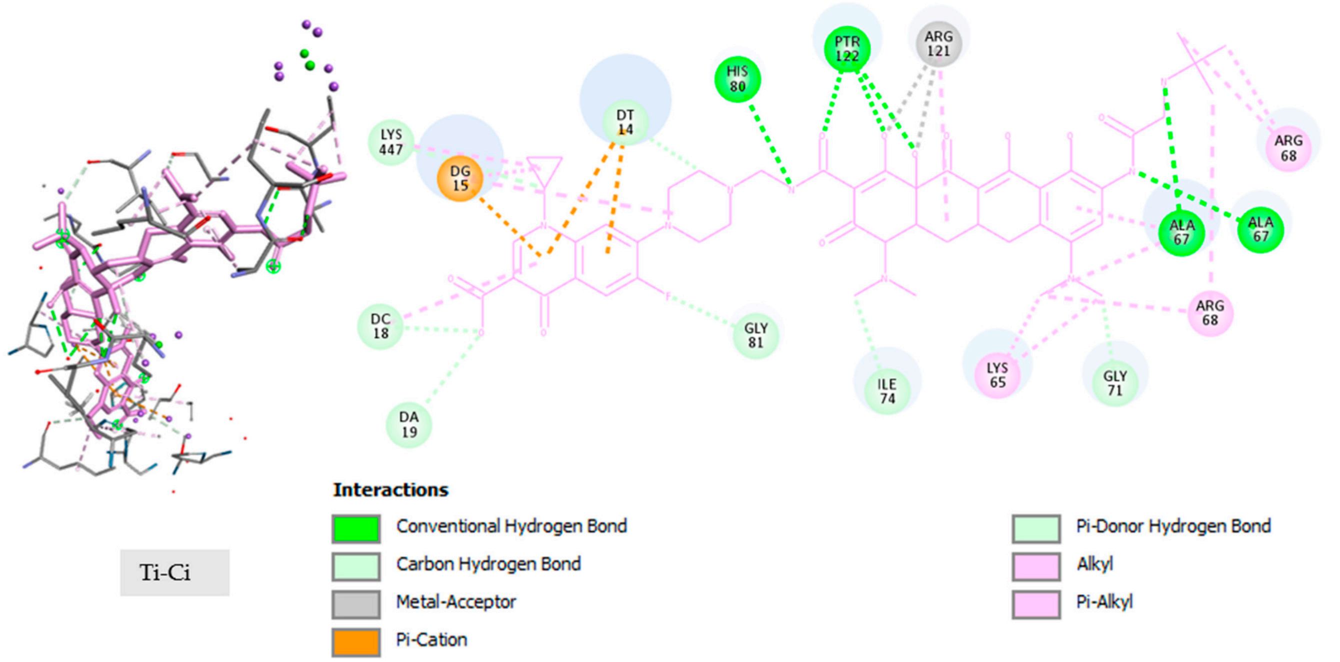

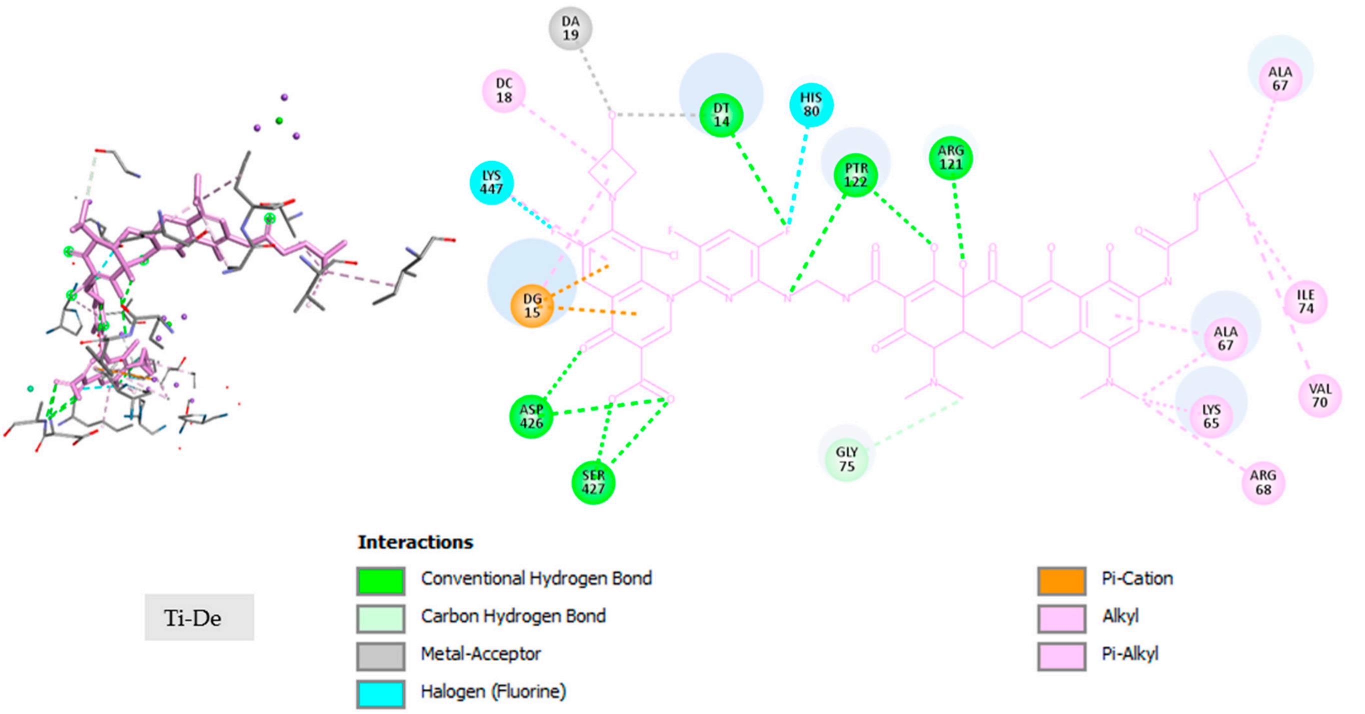

Figure S2. **(part 17 of 20)**. Interactions of the hybrids with the binding pocket of *E. coli* gyrase holo-complex with 217 bp DNA obtained in the self-docking phase for albicidin (in 3D (left) and 2D (right)).

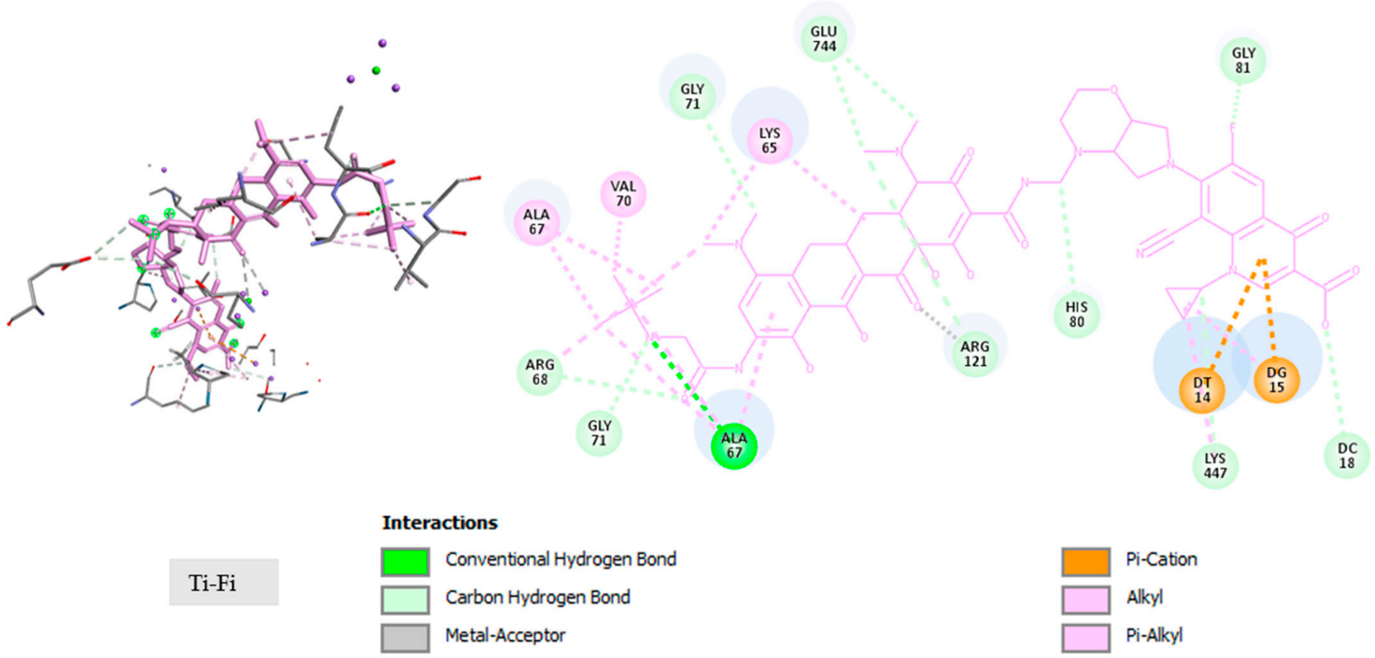

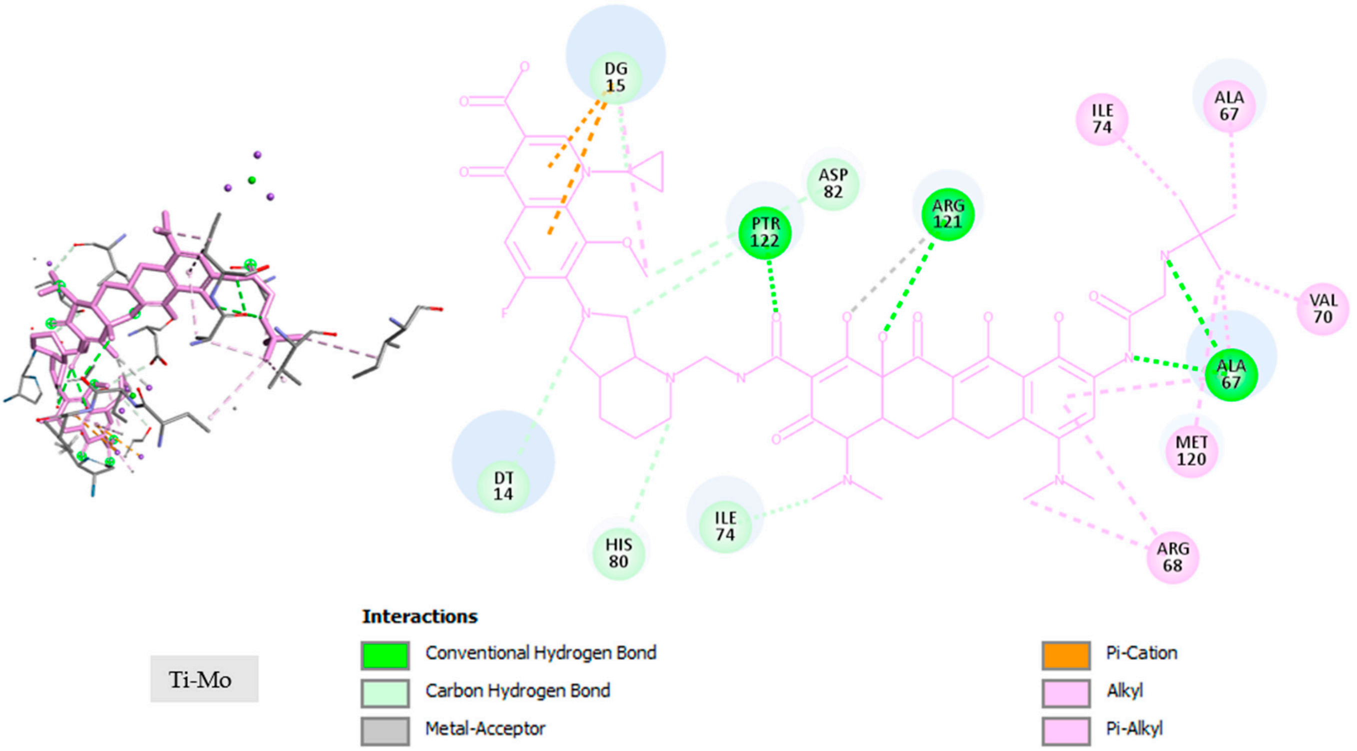

Figure S2. (part 18 of 20). Interactions of the hybrids with the binding pocket of *E. coli* gyrase holo-complex with 217 bp DNA obtained in the self-docking phase for albicidin (in 3D (left) and 2D (right)).

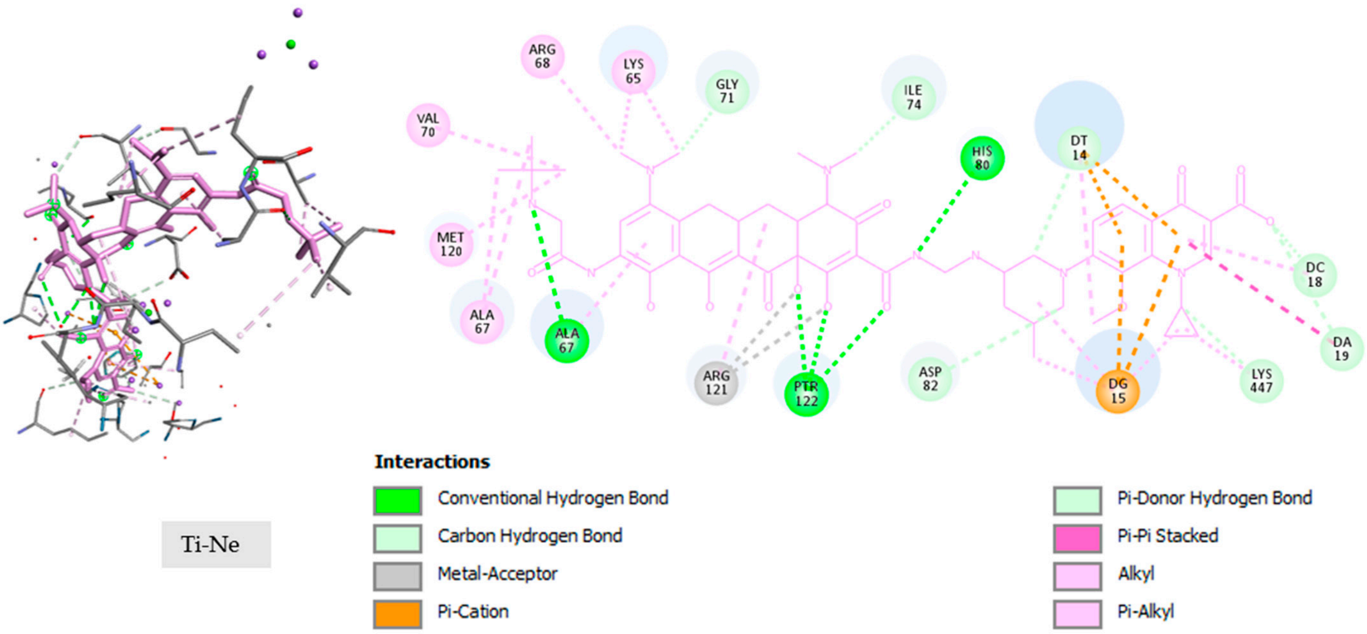

Ti-Ne

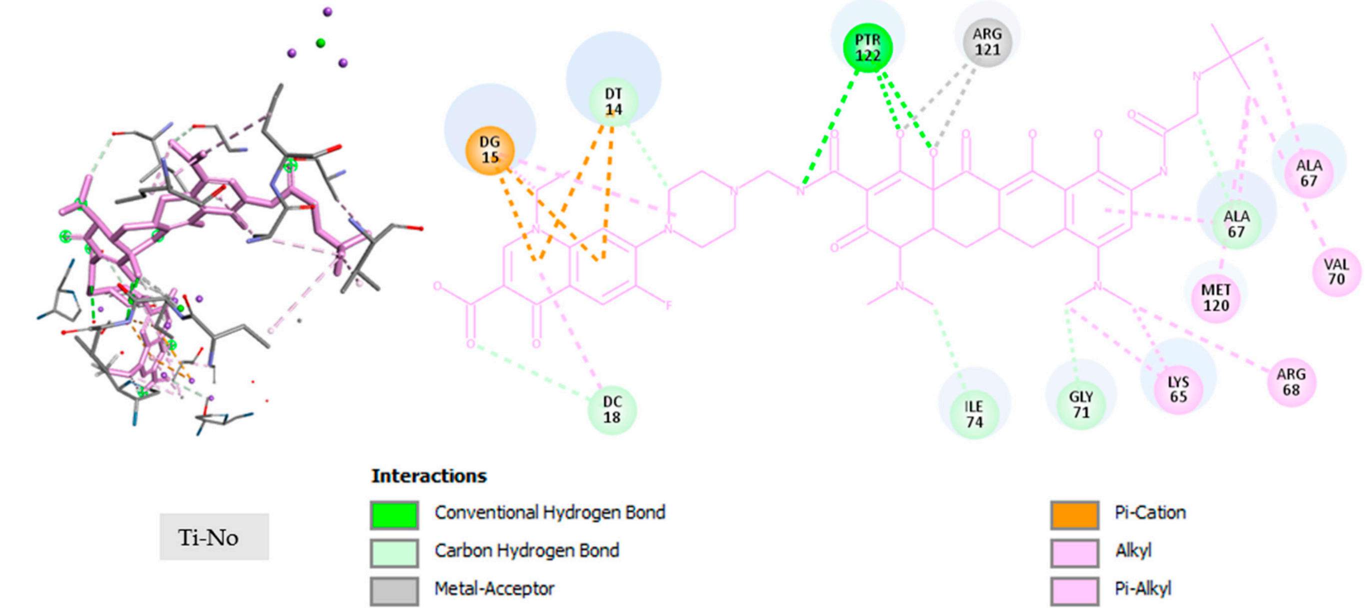

Ti-No

Figure S2. (part 19 of 20). Interactions of the hybrids with the binding pocket of *E. coli* gyrase holocomplex with 217 bp DNA obtained in the self-docking phase for albicidin (in 3D (left) and 2D (right)).

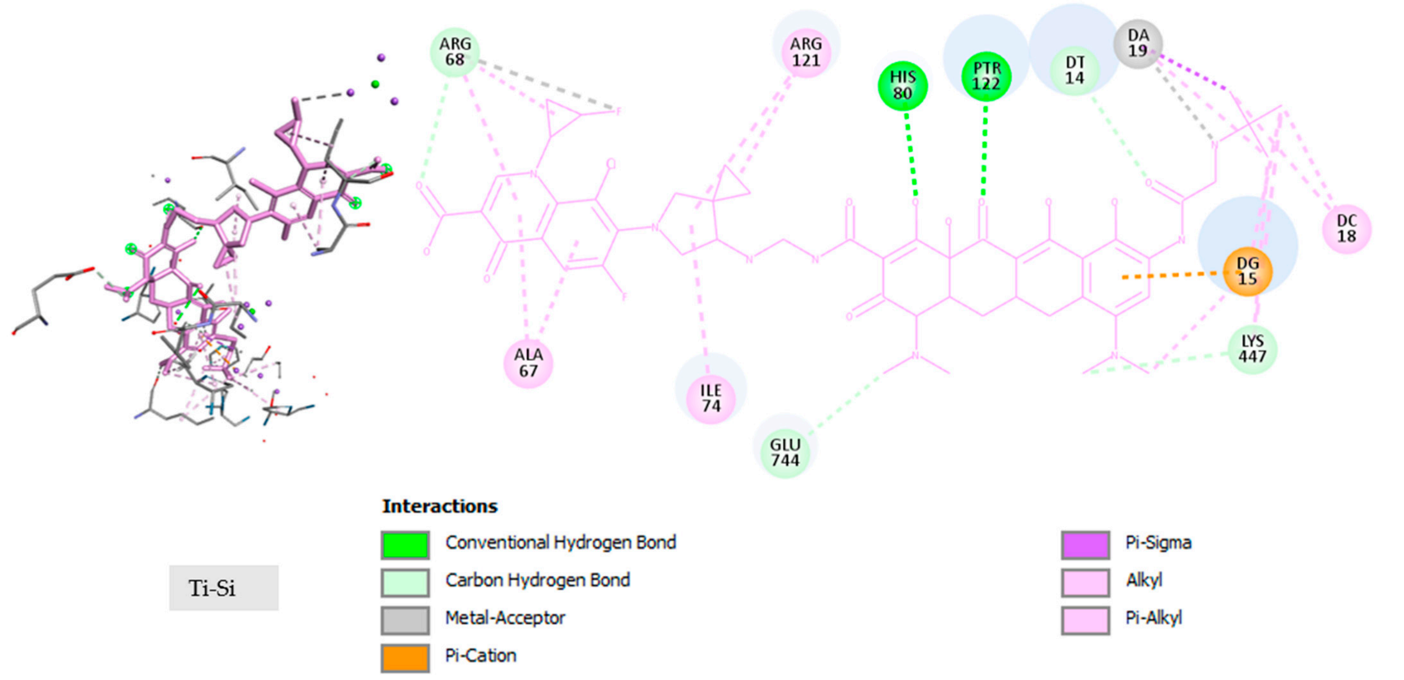

Ti-Si

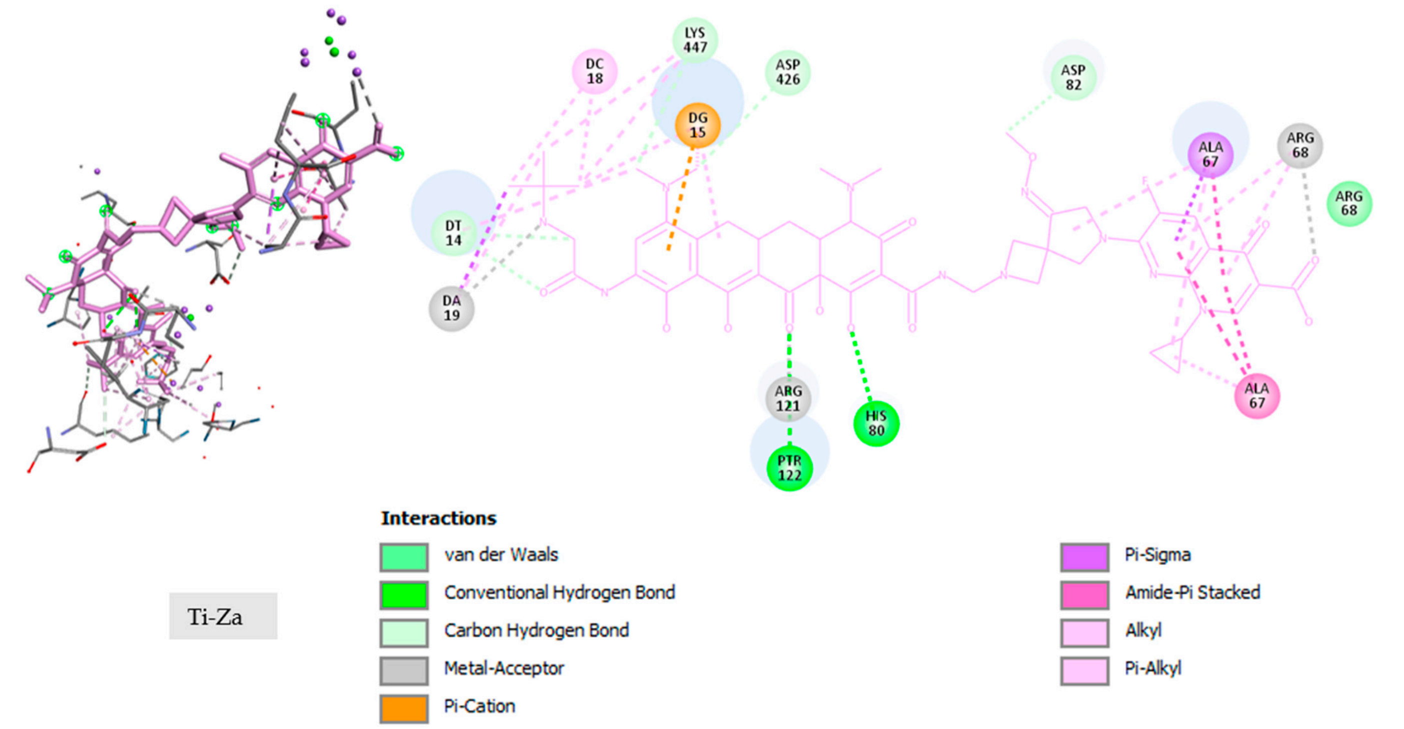

Ti-Za

Figure S2. (part 20 of 20). Interactions of the hybrids with the binding pocket of *E. coli* gyrase holo-complex with 217 bp DNA obtained in the self-docking phase for albicidin (in 3D (left) and 2D (right)).
